# Supplementary material for: Prevalence and factors associated with antenatal care service access among Indigenous women in the Chittagong Hill Tracts, Bangladesh: A cross-sectional study
Source: PLoS One. 2020 Dec 29;15(12):e0244640. doi: 10.1371/journal.pone.0244640 (PMC7771700; doi:10.1371/journal.pone.0244640)
Supplement: S1 Table — (PDF) [file pone.0244640.s001.pdf]

Supplementary table 1: All data related to ANC service access for the manuscript "Prevalence and factors associated with antenatal care service access among Indigenous women in the Chittagong Hill Tracts, Bangladesh: A cross-sectional study" (Akter et al.)

record_id	village clu	subdistrict	age_cat_new	ethnic identit	religion	pregnancy	health pro	problem	anc know	anc benef	last anc a	payment	anc info	language	bangla an	age_categorise	age_pregnancy_new	know any	HH income Status	last_anc_visit_decision_full	new_occupation_participant	trimesters anc ch	any media
1	1	Matiranga	=>30	Tripura	Sonatoni	No	No	No	Yes	Yes	No					Adult (30 & above v	became preg <19	Yes	4000-9000		Involve in income generation		No
2	1	Matiranga	25-29	Tripura	Sonatoni	Yes	Yes	Yes	No							Adult (30 & above v	became pregnant 20+ yrs	No	4000-9000		Involve in income generation		No
3	1	Matiranga	25-29	Tripura	Sonatoni	No	No	No	No							Young (15-29 yrs)	became pregnant 20+ yrs	Yes	4000-9000		Complete housewife		No
4	1	Matiranga	=>30	Tripura	Sonatoni	No	Yes	Yes	No							Adult (30 & above v	became pregnant 20+ yrs	No	4000-9000		Complete housewife		No
5	1	Matiranga	16-24	Tripura	Sonatoni	No	No	No	No							Young (15-29 yrs)	became pregnant 20+ yrs	Yes	4000-9000		Complete housewife		No
6	1	Matiranga	16-24	Tripura	Sonatoni	No	Yes	No	Yes	Yes	No					Young (15-29 yrs)	became preg <19	Yes	4000-9000		Complete housewife		No
7	2	Matiranga	16-24	Tripura	Sonatoni	No	No	No	No						No	Adult (30 & above v	became pregnant 20+ yrs	Yes	4000-9000		Complete housewife		No
8	2	Matiranga	25-29	Tripura	Sonatoni	Yes	No	Yes	Yes	Yes	Yes	No	Yes	Your own language		Young (15-29 yrs)	became preg <19	Yes	4000-9000	HW came home	Complete housewife	2nd trimesters	Yes
9	3	Matiranga	16-24	Tripura	Sonatoni	No	Yes	No	Yes	Yes	No					Young (15-29 yrs)	became pregnant 20+ yrs	Yes	20000 and above		Complete housewife		Yes
10	3	Matiranga	16-24	Tripura	Sonatoni	No	No	No	No							Young (15-29 yrs)	became preg <19	Yes	10000-19000		Involve in income generation		No
11	3	Matiranga	16-24	Tripura	Sonatoni	No	No	No	No							Young (15-29 yrs)	became preg <19	Yes	4000-9000		Complete housewife		No
12	3	Matiranga	16-24	Tripura	Sonatoni	No	No	Yes	No							Young (15-29 yrs)	became pregnant 20+ yrs	Yes	4000-9000		Complete housewife		No
13	3	Matiranga	16-24	Tripura	Sonatoni	No	Yes	Yes	No							Adult (30 & above v	became preg <19	No	4000-9000		Complete housewife		No
14	3	Matiranga	25-29	Tripura	Sonatoni	Yes	No	No	No							Young (15-29 yrs)	became preg <19	No	4000-9000		Involve in income generation		No
15	4	Matiranga	=>30	Chakma	Buddhist	No	No	Yes	No							Adult (30 & above v	became preg <19	Yes	20000 and above		Involve in income generation		No
16	4	Matiranga	16-24	Chakma	Buddhist	No	No	Yes	No							Young (15-29 yrs)	became preg <19	Yes	4000-9000		Complete housewife		No
17	4	Matiranga	16-24	Chakma	Buddhist	No	No	No	No							Young (15-29 yrs)	became pregnant 20+ yrs	Yes	4000-9000		Complete housewife		No
18	4	Matiranga	=>30	Chakma	Buddhist	No	No	Yes	No							Adult (30 & above v	became preg <19	Yes	10000-19000		Involve in income generation		No
19	5	Matiranga	=>30	Tripura	Sonatoni	Yes	No	Yes	Yes	Yes	No				No	Adult (30 & above v	became preg <19	Yes	10000-19000		Involve in income generation		No
20	5	Matiranga	=>30	Tripura	Sonatoni	Yes	No	Yes	Yes	Yes	No					Adult (30 & above v	became preg <19	Yes	4000-9000		Involve in income generation		No
21	5	Matiranga	25-29	Tripura	Sonatoni	No	Yes	No	Yes	Yes	No					Young (15-29 yrs)	became preg <19	Yes	4000-9000		Involve in income generation		No
22	5	Matiranga	=>30	Tripura	Sonatoni	Yes	No	Yes	Yes	Yes	No					Adult (30 & above v	became preg <19	Yes	4000-9000		Involve in income generation		No
23	5	Matiranga	=>30	Tripura	Sonatoni	Yes	Yes	Yes	Yes	Yes	Yes	Yes	No	Bangla	No	Adult (30 & above v	became preg <19	Yes	4000-9000	other	Involve in income generation	1st trimesters	No
24	5	Matiranga	25-29	Tripura	Sonatoni	No	No	Yes	Yes	Don't kno	Yes	No	No	Bangla	No	Young (15-29 yrs)	became preg <19	No	4000-9000	Perceived appro. time	Involve in income generation	2nd trimesters	No
25	5	Matiranga	=>30	Tripura	Sonatoni	Yes	Yes	No	Yes	Yes	No					Adult (30 & above v	became preg <19	Yes	4000-9000		Involve in income generation		No
26	6	Matiranga	16-24	Chakma	Buddhist	No	Yes	Yes	Yes	Yes	Yes	Yes	No	Bangla	Yes	Young (15-29 yrs)	became pregnant 20+ yrs	Yes	10000-19000	Illness/complications	Complete housewife	2nd trimesters	No
27	6	Matiranga	16-24	Chakma	Buddhist	No	Yes	No	Yes	Yes	No					Young (15-29 yrs)	became pregnant 20+ yrs	Yes	4000-9000		Complete housewife		No
28	6	Matiranga	16-24	Chakma	Buddhist	No	Yes	No	Yes	Yes	Yes	Yes	No	Bangla	Yes	Young (15-29 yrs)	became pregnant 20+ yrs	Yes	4000-9000	Perceived appro. time	Complete housewife	2nd trimesters	No
29	6	Matiranga	16-24	Chakma	Buddhist	No	Yes	No	Yes	Yes	No					Young (15-29 yrs)	became pregnant 20+ yrs	Yes	4000-9000		Complete housewife		No
30	6	Matiranga	16-24	Chakma	Buddhist	No	No	No	Yes	Don't kno	Yes	Yes	No	Bangla	No	Young (15-29 yrs)	became preg <19	Yes	4000-9000	Illness/complications	Complete housewife	2nd trimesters	No
31	6	Matiranga	16-24	Chakma	Buddhist	Yes	No	No	Yes	Yes	Yes	Yes	No	Bangla	No	Young (15-29 yrs)	became preg <19	Yes	4000-9000	HW came home	Complete housewife	2nd trimesters	No
32	7	Matiranga	16-24	Tripura	Sonatoni	No	No	No	Yes	Yes	Yes	Yes	No	Bangla	No	Young (15-29 yrs)	became preg <19	Yes	4000-9000	Illness/complications	Complete housewife	2nd trimesters	No
33	7	Matiranga	16-24	Tripura	Sonatoni	No	No	No	No							Young (15-29 yrs)	became preg <19	Yes	4000-9000		Complete housewife		No
34	7	Matiranga	16-24	Tripura	Sonatoni	Yes	No	No	Yes	Don't kno	No					Young (15-29 yrs)	became preg <19	No	10000-19000		Involve in income generation		No
35	7	Matiranga	16-24	Tripura	Sonatoni	Yes	No	No	No							Young (15-29 yrs)	became preg <19	Yes	4000-9000		Involve in income generation		No
36	7	Matiranga	16-24	Tripura	Sonatoni	No	Yes	No	No							Young (15-29 yrs)	became preg <19	Yes	4000-9000		Involve in income generation		No
37	7	Matiranga	16-24	Tripura	Sonatoni	No	No	No	No							Young (15-29 yrs)	became preg <19	Yes	4000-9000		Complete housewife		No
38	7	Matiranga	16-24	Tripura	Sonatoni	No	No	No	Yes	Don't kno	No					Young (15-29 yrs)	became preg <19	Yes	4000-9000		Complete housewife		No
39	7	Matiranga	16-24	Tripura	Sonatoni	Yes	Yes	No	No							Young (15-29 yrs)	became preg <19	Yes	4000-9000		Complete housewife		No
40	7	Matiranga	25-29	Tripura	Sonatoni	No	Yes	No	No							Young (15-29 yrs)	became preg <19	Yes	4000-9000		Complete housewife		No
41	7	Matiranga	16-24	Tripura	Sonatoni	No	No	No	No							Young (15-29 yrs)	became preg <19	Yes	4000-9000		Complete housewife		No
42	7	Matiranga	16-24	Tripura	Sonatoni	Yes	Yes	Yes	Yes	Don't kno	No					Young (15-29 yrs)	became preg <19	Yes	4000-9000		Complete housewife		No
43	7	Matiranga	16-24	Tripura	Sonatoni	No	Yes	Yes	Yes	Yes	Yes	Yes	No	Bangla	No	Young (15-29 yrs)	became preg <19	Yes	4000-9000	Confirm pregnancy	Complete housewife	1st trimesters	No
44	7	Matiranga	=>30	Tripura	Sonatoni	Yes	No	No	No							Adult (30 & above v	became preg <19	Yes	4000-9000		Involve in income generation		No
45	8	Matiranga	16-24	Tripura	Sonatoni	No	No	No	Yes	Don't kno	No					Young (15-29 yrs)	became preg <19	Yes	10000-19000		Complete housewife		No
46	8	Matiranga	25-29	Tripura	Sonatoni	No	No	Yes	Yes	Yes	Yes	No	No	Bangla	No	Young (15-29 yrs)	became preg <19	Yes	4000-9000	HW came home	Involve in income generation	2nd trimesters	No
47	8	Matiranga	16-24	Tripura	Sonatoni	Yes	No	Yes	Yes	Don't kno	No					Young (15-29 yrs)	became preg <19	Yes	4000-9000		Complete housewife		No
48	8	Matiranga	=>30	Tripura	Sonatoni	No	No	No	Yes	Yes	Yes	Yes	No	Bangla	Yes	Adult (30 & above v	became pregnant 20+ yrs	Yes	4000-9000	Know foetus condition	Complete housewife	2nd trimesters	No
49	6	Matiranga	16-24	Tripura	Sonatoni	Yes	Yes	No	Yes	Yes	Yes	Yes	No	Bangla	Yes	Adult (30 & above v	became pregnant 20+ yrs	Yes	20000 and above	Know foetus condition	Involve in income generation	2nd trimesters	Yes
50	9	Matiranga	=>30	Tripura	Sonatoni	No	Yes	No	Yes	Yes	Yes	Yes	No	Bangla	Yes	Adult (30 & above v	became pregnant 20+ yrs	Yes	20000 and above	Confirm pregnancy	Involve in income generation	1st trimesters	Yes
51	9	Matiranga	16-24	Chakma	Buddhist	No	Yes	Yes	No							Young (15-29 yrs)	became pregnant 20+ yrs	Yes	20000 and above		Complete housewife		Yes
52	9	Matiranga	=>30	Chakma	Buddhist	No	Yes	Yes	No							Adult (30 & above v	became pregnant 20+ yrs	Yes	20000 and above		Involve in income generation		Yes
53	9	Matiranga	=>30	Tripura	Sonatoni	Yes	Yes	Yes	Yes	Yes	Yes	No	Yes	Bangla	Yes	Adult (30 & above v	became pregnant 20+ yrs	Yes	20000 and above	Confirm pregnancy	Involve in income generation	1st trimesters	Yes
54	9	Matiranga	=>30	Tripura	Sonatoni	No	No	Yes	Yes	Yes	Yes	Yes	No	Bangla	Yes	Adult (30 & above v	became pregnant 20+ yrs	Yes	20000 and above	Confirm pregnancy	Involve in income generation	1st trimesters	Yes
55	10	Matiranga	25-29	Chakma	Buddhist	No	Yes	No	Yes	Yes	Yes	No	No	Bangla	Yes	Young (15-29 yrs)	became preg <19	Yes	10000-19000	Illness/complications	Involve in income generation	2nd trimesters	Yes
56	10	Matiranga	16-24	Chakma	Buddhist	No	Yes	No	Yes	Yes	Yes	Yes	No	Bangla	Yes	Young (15-29 yrs)	became preg <19	Yes	4000-9000	Confirm pregnancy	Complete housewife	1st trimesters	Yes
57	10	Matiranga	25-29	Chakma	Buddhist	No	Yes	No	Yes	Yes	Yes	No	No	Bangla	Yes	Young (15-29 yrs)	became pregnant 20+ yrs	Yes	4000-9000	Illness/complications	Complete housewife	1st trimesters	No
58	10	Matiranga	25-29	Chakma	Buddhist	No	Yes	No	Yes	Yes	Yes	Yes	No	Bangla	No	Young (15-29 yrs)	became pregnant 20+ yrs	Yes	10000-19000	Foetus sex	Complete housewife	2nd trimesters	No
59	10	Matiranga	25-29	Chakma	Buddhist	No	No	No	Yes	Yes	Yes	Yes	No	Bangla	No	Young (15-29 yrs)	became pregnant 20+ yrs	Yes	4000-9000	Know foetus condition	Complete housewife	2nd trimesters	No
60	10	Matiranga	=>30	Chakma	Buddhist	No	No	No	Yes	Yes	Yes	No	Yes	Your own	Yes	Adult (30 & above v	became pregnant 20+ yrs	Yes	4000-9000	HW came home	Complete housewife	2nd trimesters	No
61	10	Matiranga	25-29	Chakma	Buddhist	Yes	No	No	No							Young (15-29 yrs)	became pregnant 20+ yrs	Yes	4000-9000		Involve in income generation		No
62	10	Matiranga	=>30	Chakma	Buddhist	No	No	Yes	Yes	Yes	Yes	Yes	No	Bangla	No	Adult (30 & above v	became preg <19	Yes	20000 and above	Illness/complications	Complete housewife	2nd trimesters	No
63	11	Matiranga	25-29	Marma	Buddhist	No	No	No	No							Young (15-29 yrs)	became pregnant 20+ yrs	Yes	4000-9000		Complete housewife		No
64	11	Matiranga	=>30	Marma	Buddhist	Yes	No	Yes	Yes	Don't kno	Yes	No	No	Bangla	No	Adult (30 & above v	became preg <19	Yes	4000-9000	Confirm pregnancy	Complete housewife	1st trimesters	No
65	11	Matiranga	25-29	Marma	Buddhist	No	Yes	Yes	Yes	Don't kno	Yes	No	No	Bangla	No	Young (15-29 yrs)	became preg <19	Yes	4000-9000	HW came home	Involve in income generation	2nd trimesters	No
66	11	Matiranga	16-24	Marma	Buddhist	No	No	No	No							Young (15-29 yrs)	became preg <19	Yes	4000-9000		Complete housewife		No
67	11	Matiranga	16-24	Marma	Buddhist	No	No	No	Yes	Don't kno	No					Young (15-29 yrs)	became preg <19	Yes	4000-9000		Complete housewife		No
68	11	Matiranga	16-24	Marma	Buddhist	No	No	No	No							Young (15-29 yrs)	became pregnant 20+ yrs	Yes	4000-9000		Complete housewife		No
69	11	Matiranga	16-24	Marma	Buddhist	Yes	No	No	No							Young (15-29 yrs)	became preg <19	Yes	4000-9000		Complete housewife		No
70	11	Matiranga	=>30	Marma	Buddhist	Yes	No	No	No							Adult (30 & above v	became pregnant 20+ yrs	Yes	4000-9000		Complete housewife		No
71	11	Matiranga	25-29	Marma	Buddhist	No	No	No	Yes	Yes	Yes	No	No	Bangla	Yes	Young (15-29 yrs)	became pregnant 20+ yrs	Yes	4000-9000	HW came home	Complete housewife	2nd trimesters	No
72	12	Matiranga	16-24	Tripura	Sonatoni	No	No	No	Yes	Don't kno	No					Young (15-29 yrs)	became preg <19	Yes	4000-9000		Complete housewife		No
73	12	Matiranga	16-24	Tripura	Sonatoni	No	No	No	No							Young (15-29 yrs)	became preg <19	No	4000-9000		Complete housewife		No
74	12	Matiranga	16-24	Tripura	Sonatoni	No	No	No	No							Young (15-29 yrs)	became preg <19	Yes	4000-9000		Complete housewife		No
75	12	Matiranga	=>30	Tripura	Sonatoni	No	No	No	No							Adult (30 & above v	became pregnant 20+ yrs	Yes	4000-9000		Complete housewife		No
76	12	Matiranga	16-24	Tripura	Sonatoni	No	No	Yes	Yes	Yes	Yes	No	No	Bangla	No	Young (15-29 yrs)	became preg <19	Yes	4000-9000	HW came home	Complete housewife	1st trimesters	No
77	13	Matiranga	25-29	Tripura	Sonatoni	Yes	Yes	Yes	Yes	Yes	Yes	Yes	No	Bangla	Yes	Young (15-29 yrs)	became preg <19	Yes	4000-9000	Know foetus condition	Complete housewife	2nd trimesters	No
78	13	Matiranga	25-29	Tripura	Sonatoni	No	No	No	Yes	Yes	Yes	Yes	No	Bangla	No	Young (15-29 yrs)	became preg <19	Yes	4000-9000	Confirm pregnancy	Involve in income generation	1st trimesters	No
79	13	Matiranga	16-24	Tripura	Sonatoni	Yes	No	No	No							Young (15-29 yrs)	became preg <19	Yes	20000 and above		Involve in income generation		No
80	14	Matiranga	25-29	Marma	Buddhist	No	No	No	No							Young (15-29 yrs)	became preg <19	Yes	4000-9000		Complete housewife		No
81	14	Matiranga	16-24	Marma	Buddhist	No	No	No	Yes	Don't kno	No					Young (15-29 yrs)	became preg <19	Yes	20000 and above		Complete housewife		Yes
82	14	Matiranga	16-24	Marma	Buddhist	No	No	No	No							Young (15-29 yrs)	became preg <19	Yes	4000-9000		Complete housewife		Yes
83	14	Matiranga	16-24	Marma	Buddhist	No	No	No	No							Young (15-29 yrs)	became preg <19	Yes	4000-9000		Complete housewife		No
84	14	Matiranga	16-24	Marma	Buddhist	No	No	No	No							Young (15-29 yrs)	became preg <19	Yes	4000-9000		Complete housewife		No
85	15	Matiranga	25-29	Marma	Buddhist	No	No	No	Yes	Yes	Yes	Yes	No	Bangla	Yes	Young (15-29 yrs)	became pregnant 20+ yrs	Yes	20000 and above	HW came home	Complete housewife	2nd trimesters	Yes
86	15	Matiranga	25-29	Marma	Buddhist	No	Yes	Yes	Yes	Yes	Yes	Yes	No	Bangla	Yes	Young (15-29 yrs)	became pregnant 20+ yrs	Yes	20000 and above	Know foetus condition	Complete housewife	2nd trimesters	Yes
87	15	Matiranga	16-24	Marma	Buddhist	No	No	No	Yes	Yes	Yes	No	No	Bangla	No	Young (15-29 yrs)	became preg <19	Yes	20000 and above	Know foetus condition	Involve in income generation	2nd trimesters	No
88	16	Matiranga	25-29	Tripura	Sonatoni	No	No	No	Yes	Yes	Yes	Yes	No	Bangla	Yes	Young (15-29 yrs)	became preg <19	Yes	10000-19000	Illness/complications	Complete housewife	1st trimesters	Yes
89	16	Matiranga	16-24	Tripura	Sonatoni	Yes	No	No	No							Young (15-29 yrs)	became preg <19	Yes	4000-9000		Complete housewife		No
90	16	Matiranga	16-24	Tripura	Sonatoni	No	Yes	Yes	Yes	Don't kno	Yes	Yes	No	Bangla	No	Young (15-29 yrs)	became preg <19	Yes	10000-19000	Foetus sex	Complete housewife	2nd trimesters	No
91	16	Matiranga	25-29	Tripura	Sonatoni	No	No	No	Yes	Yes	No					Young (15-29 yrs)	became pregnant 20+ yrs	Yes	10000-19000		Complete housewife		Yes
92	16	Matiranga	16-24	Tripura	Sonatoni	No	No	Yes	Yes	Don't kno	Yes	Yes	No	Bangla	No	Young (15-29 yrs)	became preg <19	Yes	10000-19000	HW came home	Complete housewife	2nd trimesters	No
93	16	Matiranga	16-24	Tripura	Sonatoni	No	No	No	No							Young (15-29 yrs)	became preg <19	Yes	4000-9000		Complete housewife		No
94	16	Matiranga	16-24	Tripura	Sonatoni	No	No	No	Yes	Yes	Yes	No	No	Bangla	No	Young (15-29 yrs)	became preg <19	Yes	4000-9000	HW came home	Complete housewife	2nd trimesters	No
95	16	Matiranga	16-24	Tripura	Sonatoni	No	No	No	No							Young (15-29 yrs)	became preg <19	Yes	4000-9000		Complete housewife		No
96	16	Matiranga	25-29	Tripura	Sonatoni	No	No	Yes	No							Young (15-29 yrs)	became preg <19	Yes	20000 and above		Complete housewife		Yes
97	16	Matiranga	16-24	Tripura	Sonatoni	No	Yes	Yes	Yes	Yes	Yes	No	No	Bangla	Yes	Young (15-29 yrs)	became preg <19	Yes	4000-9000	Illness/complications	Involve in income generation	2nd trimesters	No
98	16	Matiranga	16-24	Tripura	Sonatoni	No	No	Yes	No							Young (15-29 yrs)	became preg <19	Yes	4000-9000		Complete housewife		No
99	17	Matiranga	16-24	Tripura	Sonatoni	No	No	No	Yes	Yes	Yes	No	No	Bangla	No	Young (15-29 yrs)	became preg <19	Yes	4000-9000	Illness/complications	Involve in income generation	2nd trimesters	No
100	17	Matiranga	25-29	Tripura	Sonatoni	No	No	No	Yes	Yes	Yes	No	No	Bangla	No	Young (15-29 yrs)	became preg <19	Yes	4000-9000	Confirm pregnancy	Involve in income generation	1st trimesters	No
101	17	Matiranga	16-24	Tripura	Sonatoni	No	No	No	Yes	Yes	Yes	Yes	Yes	Your own language		Young (15-29 yrs)	became preg <19	Yes	20000 and above	Foetus sex	Complete housewife	2nd trimesters	No
102	17	Matiranga	=>30	Tripura	Sonatoni	Yes	Yes	No	Yes	Yes	Yes	Yes	Yes	Bangla	Yes	Adult (30 & above v	became pregnant 20+ yrs	Yes	20000 and above	Confirm pregnancy	Involve in income generation	1st trimesters	Yes
103	17	Matiranga	16-24	Tripura	Sonatoni	Yes	No	Yes	Yes	Yes	Yes	No	No	Bangla	Yes	Young (15-29 yrs)	became preg <19	Yes	10000-19000	Know foetus condition	Complete housewife	2nd trimesters	No
104	18	Matiranga	25-29	Tripura	Sonatoni	No	Yes	Yes	Yes	Yes	Yes	Yes	No	Bangla	No	Young (15-29 yrs)	became preg <19	Yes	10000-19000	Confirm pregnancy	Involve in income generation	1st trimesters	No
105	18	Matiranga	16-24	Tripura	Sonatoni	Yes	Yes	Yes	Yes	Yes	Yes	Yes	No	Bangla		Young (15-29 yrs)	became preg <19	Yes	4000-9000	Confirm pregnancy	Involve in income generation	1st trimesters	No
106	18	Matiranga	=>30	Tripura	Sonatoni	Yes	Yes	Yes	Yes	Don't kno	No					Adult (30 & above v	became preg <19	Yes	4000-9000		Involve in income generation		No
107	18	Matiranga	16-24	Tripura	Sonatoni	Yes	No	No	No							Young (15-29 yrs)	became preg <19	Yes	4000-9000		Involve in income generation		No
108	18	Matiranga	25-29	Tripura	Sonatoni	No	No	No	No							Young (15-29 yrs)	became preg <19	Yes	10000-19000		Involve in income generation		No
109	19	Matiranga	16-24	Tripura	Sonatoni	No	No	Yes	Yes	Yes	No					Young (15-29 yrs)	became preg <19	Yes	4000-9000		Involve in income generation		No
110	19	Matiranga	16-24	Tripura	Sonatoni	No	No	No	No							Young (15-29 yrs)	became preg <19	Yes	10000-19000		Complete housewife		No
111	19	Matiranga	25-29	Tripura	Sonatoni	Yes	Yes	Yes	No							Young (15-29 yrs)	became preg <19	Yes	4000-9000		Complete housewife		No
112	19	Matiranga	16-24	Tripura	Sonatoni	Yes	No	Yes	Yes	Don't kno	No					Young (15-29 yrs)	became preg <19	Yes	10000-19000		Complete housewife		No
113	19	Matiranga	16-24	Tripura	Sonatoni	No	No	No	No							Young (15-29 yrs)	became preg <19	Yes	4000-9000		Complete housewife		No
114	19	Matiranga	25-29	Tripura	Sonatoni	Yes	No	Yes	No							Young (15-29 yrs)	became preg <19	No	4000-9000		Involve in income generation		No
115	19	Matiranga	16-24	Tripura	Sonatoni	No	No	Yes	No							Young (15-29 yrs)	became preg <19	Yes	10000-19000		Complete housewife		No
116	13	Matiranga	16-24	Tripura	Sonatoni	No	Yes	Yes	Yes	Yes	Yes	No	No	Bangla	No	Young (15-29 yrs)	became preg <19	Yes	4000-9000	Illness/complications	Involve in income generation	2nd trimesters	No
117	13	Matiranga	16-24	Tripura	Sonatoni	Yes	Yes	Yes	Yes	Don't kno	No					Young (15-29 yrs)	became preg <19	Yes	4000-9000		Complete housewife		No
118	13	Matiranga	16-24	Tripura	Sonatoni	Yes	Yes	Yes	Yes	Don't kno	No					Young (15-29 yrs)	became preg <19	Yes	10000-19000		Complete housewife		No
119	13	Matiranga	16-24	Tripura	Sonatoni	Yes	Yes	Yes	Yes	Don't kno	No					Young (15-29 yrs)	became preg <19	Yes	4000-9000		Complete housewife		No
120	20	Matiranga	16-24	Tripura	Sonatoni	No	No	No	Yes	Yes	Yes	No	No	Bangla	Yes	Young (15-29 yrs)	became preg <19	Yes	10000-19000	Perceived appro. time	Complete housewife	2nd trimesters	No
121	20	Matiranga	25-29	Tripura	Sonatoni	No	Yes	Yes	Yes	Yes	Yes	No	Yes	Your own language		Young (15-29 yrs)	became pregnant 20+ yrs	Yes	10000-19000	Perceived appro. time	Complete housewife	2nd trimesters	No
122	20	Matiranga	16-24	Tripura	Sonatoni	No	No	No	No							Young (15-29 yrs)	became pregnant 20+ yrs	Yes	10000-19000		Complete housewife		No
123	20	Matiranga	16-24	Tripura	Sonatoni	No	No	No	No							Young (15-29 yrs)	became preg <19	Yes	4000-9000		Complete housewife		No
124	20	Matiranga	16-24	Tripura	Sonatoni	No	Yes	Yes	Yes	Yes	Yes	No	Yes	Your own language		Young (15-29 yrs)	became preg <19	Yes	4000-9000	Perceived appro. time	Complete housewife	2nd trimesters	No
125	20	Matiranga	16-24	Tripura	Sonatoni	No	No	No	Yes	Yes	Yes	Yes	No	Bangla	Yes	Young (15-29 yrs)	became preg <19	Yes	10000-19000	Foetus sex	Involve in income generation	3rd trimesters	No
126	20	Matiranga	16-24	Tripura	Sonatoni	Yes	No	Yes	Yes	Yes	Yes	Yes	No	Bangla	No	Young (15-29 yrs)	became preg <19	Yes	4000-9000	Know foetus condition	Involve in income generation	2nd trimesters	No
127	21	Matiranga	16-24	Tripura	Sonatoni	No	No	No	No							Young (15-29 yrs)	became preg <19	Yes	4000-9000		Involve in income generation		No
128	21	Matiranga	25-29	Tripura	Sonatoni	No	No	No	No							Young (15-29 yrs)	became preg <19	Yes	4000-9000		Involve in income generation		No
129	21	Matiranga	16-24	Tripura	Sonatoni	Yes	No	No	No							Young (15-29 yrs)	became preg <19	No	4000-9000		Involve in income generation		No
130	21	Matiranga	16-24	Tripura	Sonatoni	No	No	No	Yes	Don't kno	No					Young (15-29 yrs)	became preg <19	Yes	4000-9000		Complete housewife		No
131	21	Matiranga	16-24	Tripura	Sonatoni	No	No	Yes	No							Young (15-29 yrs)	became preg <19	Yes	4000-9000		Involve in income generation		No
132	21	Matiranga	16-24	Tripura	Sonatoni	No	Yes	Yes	Yes	Yes	Yes	No	No	Bangla	Yes	Young (15-29 yrs)	became pregnant 20+ yrs	Yes	4000-9000	Illness/complications	Complete housewife	2nd trimesters	No
133	21	Matiranga	16-24	Tripura	Sonatoni	No	Yes	Yes	Yes	Don't kno	Yes	No	No	Bangla	No	Young (15-29 yrs)	became preg <19	Yes	10000-19000	HW came home	Involve in income generation	2nd trimesters	No
134	21	Matiranga	16-24	Tripura	Sonatoni	Yes	No	Yes	Yes	Don't kno	Yes	No	No	Bangla	No	Young (15-29 yrs)	became preg <19	No	4000-9000	Illness/complications	Complete housewife	3rd trimesters	No
135	21	Matiranga	25-29	Tripura	Sonatoni	No	Yes	Yes	Yes	Yes	Yes	No	No	Bangla	Yes	Young (15-29 yrs)	became pregnant 20+ yrs	Yes	10000-19000	Perceived appro. time	Involve in income generation	2nd trimesters	No
136	21	Matiranga	16-24	Tripura	Sonatoni	No	Yes	No	Yes	Yes	Yes	Yes	No	Bangla	No	Young (15-29 yrs)	became preg <19	Yes	10000-19000	Illness/complications	Complete housewife	2nd trimesters	No
137	21	Matiranga	25-29	Tripura	Sonatoni	No	No	Yes	Yes	Yes	Yes	Yes	No	Bangla	Yes	Young (15-29 yrs)	became pregnant 20+ yrs	Yes	20000 and above	Illness/complications	Complete housewife	2nd trimesters	No
138	22	Matiranga	16-24	Marma	Buddhist	No	No	No	Yes	Yes	Yes	Yes	Yes	Bangla	Yes	Adult (30 & above v	became pregnant 20+ yrs	Yes	20000 and above	Confirm pregnancy	Complete housewife	1st trimesters	Yes
139	22	Matiranga	16-24	Marma	Buddhist	No	No	No	Yes	Yes	Yes	Yes	Yes	Bangla	Yes	Adult (30 & above v	became pregnant 20+ yrs	Yes	20000 and above	Confirm pregnancy	Complete housewife	1st trimesters	Yes
140	22	Matiranga	16-24	Marma	Buddhist	No	No	Yes	No							Young (15-29 yrs)	became preg <19	Yes	4000-9000		Complete housewife		No
141	22	Matiranga	16-24	Marma	Buddhist	No	No	Yes	No							Young (15-29 yrs)	became preg <19	Yes	10000-19000		Complete housewife		No
142	22	Matiranga	25-29	Marma	Buddhist	No	Yes	Yes	Yes	Yes	Yes	Yes	No	Bangla	Yes	Young (15-29 yrs)	became pregnant 20+ yrs	Yes	4000-9000	Know foetus condition	Complete housewife	2nd trimesters	No
143	22	Matiranga	16-24	Marma	Buddhist	No	No	No	No							Young (15-29 yrs)	became pregnant 20+ yrs	Yes	10000-19000		Complete housewife		Yes
144	22	Matiranga	=>30	Marma	Buddhist	No	No	Yes	Yes	Yes	Yes	Yes	No	Bangla	Yes	Adult (30 & above v	became pregnant 20+ yrs	Yes	10000-19000	Confirm pregnancy	Complete housewife	1st trimesters	No
145	22	Matiranga	25-29	Marma	Buddhist	No	Yes	No	Yes	Yes	Yes	Yes	No	Bangla	Yes	Young (15-29 yrs)	became pregnant 20+ yrs	Yes	10000-19000	Confirm pregnancy	Complete housewife	1st trimesters	Yes
146	22	Matiranga	25-29	Marma	Buddhist	No	No	Yes	Yes	Yes	Yes	Yes	Yes	Bangla	Yes	Young (15-29 yrs)	became pregnant 20+ yrs	Yes	10000-19000	Illness/complications	Complete housewife	1st trimesters	No
147	22	Matiranga	25-29	Marma	Buddhist	No	Yes	Yes	Yes	Yes	Yes	Yes	No	Bangla	No	Young (15-29 yrs)	became pregnant 20+ yrs	Yes	10000-19000	Confirm pregnancy	Complete housewife	1st trimesters	Yes
148	22	Matiranga	25-29	Marma	Buddhist	No	No	Yes	Yes	Yes	Yes	Yes	No	Bangla	No	Young (15-29 yrs)	became preg <19	Yes	10000-19000	Foetus sex	Complete housewife	2nd trimesters	No
149	22	Matiranga	=>30	Marma	Buddhist	Yes	No	No	Yes	Don't kno	Yes	Yes	No	Bangla	No	Adult (30 & above v	became pregnant 20+ yrs	Yes	4000-9000	Foetus sex	Complete housewife	3rd trimesters	No
150	22	Matiranga	16-24	Marma	Buddhist	No	No	No	Yes	Don't kno	Yes	Yes	No	Bangla	No	Young (15-29 yrs)	became preg <19	Yes	4000-9000	Confirm pregnancy	Complete housewife	1st trimesters	No
151	22	Matiranga	25-29	Marma	Buddhist	No	No	Yes	Yes	Yes	Yes	No	Yes	Bangla	Yes	Young (15-29 yrs)	became preg <19	Yes	20000 and above	Illness/complications	Complete housewife	2nd trimesters	Yes
152	23	Matiranga	25-29	Marma	Buddhist	No	Yes	Yes	Yes	Yes	Yes	Yes	Yes	Bangla	Yes	Young (15-29 yrs)	became pregnant 20+ yrs	Yes	20000 and above	Foetus sex	Involve in income generation	2nd trimesters	Yes
153	23	Matiranga	=>30	Marma	Buddhist	No	No	Yes	Yes	Yes	Yes	Yes	Yes	Bangla	Yes	Adult (30 & above v	became pregnant 20+ yrs	Yes	10000-19000	Confirm pregnancy	Complete housewife	1st trimesters	Yes
154	23	Matiranga	=>30	Marma	Buddhist	Yes	Yes	Yes	Yes	Yes	Yes	Yes	No	Bangla	No	Adult (30 & above v	became preg <19	Yes	20000 and above	Foetus sex	Complete housewife	2nd trimesters	Yes
155	23	Matiranga	=>30	Marma	Buddhist	No	No	Yes	Yes	Don't kno	No					Adult (30 & above v	became pregnant 20+ yrs	Yes	20000 and above		Involve in income generation		No
156	24	Matiranga	=>30	Marma	Buddhist	Yes	No	No	No							Adult (30 & above v	became preg <19	Yes	4000-9000		Involve in income generation		No
157	24	Matiranga	25-29	Marma	Buddhist	Yes	No	Yes	No							Young (15-29 yrs)	became pregnant 20+ yrs	No	4000-9000		Complete housewife		No
158	24	Matiranga	25-29	Marma	Buddhist	No	Yes	Yes	Yes	Yes	Yes	Yes	Yes	Bangla	Yes	Young (15-29 yrs)	became pregnant 20+ yrs	Yes	20000 and above	Foetus sex	Complete housewife	2nd trimesters	Yes
159	24	Matiranga	25-29	Marma	Buddhist	Yes	No	Yes	Yes	Yes	No					Adult (30 & above v	became pregnant 20+ yrs	Yes	20000 and above		Complete housewife		Yes
160	24	Matiranga	=>30	Marma	Buddhist	Yes	No	Yes	Yes	Yes	Yes	No	No	Bangla	Yes	Adult (30 & above v	became preg <19	Yes	10000-19000	Confirm pregnancy	Complete housewife	1st trimesters	Yes
161	1	Matiranga	16-24	Tripura	Sonatoni	No	No	Yes	Yes	Yes	Yes	No	No	Bangla	Yes	Young (15-29 yrs)	became preg <19	Yes	10000-19000	Know foetus condition	Complete housewife	2nd trimesters	No
162	22	Matiranga	25-29	Marma	Buddhist	No	Yes	Yes	Yes	Yes	Yes	Yes	Yes	Bangla	Yes	Adult (30 & above v	became pregnant 20+ yrs	Yes	10000-19000	Foetus sex	Involve in income generation	2nd trimesters	No
163	25	Khagrachhari s	=>30	Marma	Buddhist	No	No	Yes	Yes	Yes	Yes	Yes	Yes	Bangla	Yes	Adult (30 & above v	became pregnant 20+ yrs	Yes	20000 and above	Know foetus condition	Complete housewife	2nd trimesters	Yes
164	25	Khagrachhari s	16-24	Marma	Buddhist	No	Yes	No	Yes	Yes	Yes	No	No	Bangla	Yes	Young (15-29 yrs)	became preg <19	Yes	20000 and above	Confirm pregnancy	Complete housewife	1st trimesters	Yes
165	25	Khagrachhari s	=>30	Marma	Buddhist	No	No	No	Yes	Yes	Yes	Yes	No	Bangla	No	Adult (30 & above v	became preg <19	Yes	20000 and above	Foetus sex	Complete housewife	2nd trimesters	Yes
166	25	Khagrachhari s	16-24	Marma	Buddhist	No	No	No	Yes	Don't kno	Yes	Yes	No	Bangla	No	Young (15-29 yrs)	became preg <19	Yes	10000-19000	Foetus sex	Complete housewife	2nd trimesters	No
167	25	Khagrachhari s	25-29	Tripura	Sonatoni	No	Yes	No	Yes	Yes	Yes	No	Yes	Bangla	Yes	Young (15-29 yrs)	became pregnant 20+ yrs	Yes	20000 and above	Know foetus condition	Complete housewife	2nd trimesters	Yes
168	25	Khagrachhari s	=>30	Marma	Buddhist	No	No	Yes	Yes	Yes	Yes	Yes	No	Bangla	Yes	Adult (30 & above v	became pregnant 20+ yrs	Yes	20000 and above	Foetus sex	Involve in income generation	2nd trimesters	Yes
169	25	Khagrachhari s	25-29	Marma	Buddhist	No	Yes	No	Yes	Yes	Yes	No	No	Bangla	Yes	Young (15-29 yrs)	became pregnant 20+ yrs	Yes	20000 and above	Confirm pregnancy	Involve in income generation	1st trimesters	Yes
170	25	Khagrachhari s	=>30	Marma	Buddhist	No	Yes	Yes	Yes	Yes	Yes	No	Yes	Bangla	Yes	Adult (30 & above v	became pregnant 20+ yrs	Yes	20000 and above	Confirm pregnancy	Involve in income generation	1st trimesters	Yes
171	25	Khagrachhari s	=>30	Marma	Buddhist	Yes	Yes	Yes	Yes	Yes	Yes	Yes	Yes	Bangla	Yes	Adult (30 & above v	became pregnant 20+ yrs	Yes	20000 and above	Confirm pregnancy	Involve in income generation	1st trimesters	Yes
172	25	Khagrachhari s	25-29	Marma	Buddhist	No	Yes	Yes	Yes	Yes	Yes	Yes	No	Bangla		Young (15-29 yrs)	became pregnant 20+ yrs	Yes	10000-19000	Perceived appro. time	Complete housewife	2nd trimesters	Yes
173	25	Khagrachhari s	16-24	Marma	Buddhist	No	Yes	Yes	Yes	Yes	Yes	No	Yes	Bangla	Yes	Young (15-29 yrs)	became pregnant 20+ yrs	Yes	10000-19000	Know foetus condition	Complete housewife	2nd trimesters	Yes
174	25	Khagrachhari s	25-29	Marma	Buddhist	No	Yes	Yes	Yes	Yes	Yes	Yes	No	Bangla	Yes	Young (15-29 yrs)	became pregnant 20+ yrs	Yes	20000 and above	Illness/complications	Complete housewife	2nd trimesters	Yes
175	25	Khagrachhari s	16-24	Tripura	Sonatoni	No	Yes	Yes	Yes	Yes	Yes	Yes	Yes	Your own language		Young (15-29 yrs)	became pregnant 20+ yrs	Yes	20000 and above	Know foetus condition	Complete housewife	3rd trimesters	Yes

record_id	anc service use new	partner o	distance k	school_attend_new3cat	school attnd partner new3ca	anc info	anc info	anc info	anc info	total_preg_2cat	school_attend_new2cat	school attend partner ne	know_nearest_facility_merged4cat	know any	last_preg_prob_severity	ANC_source_info	occupation partner 2ca	ANC_4visit_3cat	anc_pay_3catNew
1		Service hc	6-10km	Didn't attend - primary	Didn't attend - Primary	No	Yes	No	No	3 & above pregnancies	didn't-junior school	didn't-junior school	Community_based centres	Yes	No problem reported	Health complex & providers	Employed/Business	Did not access	
2		Service hc	6-10km	Didn't attend - primary	Didn't attend - Primary	No	No	No	No	3 & above pregnancies	didn't-junior school	didn't-junior school	Do not know	No	Mild		Employed/Business	Did not access	
3		daily lab/f	6-10km	Didn't attend - primary	Secondary school	No	No	No	No	1-2 pregnancies	didn't-junior school	didn't-junior school	Community_based centres	Yes	No problem reported		Daily labor/Farmer	Did not access	
4		Service hc	6-10km	Didn't attend - primary	Didn't attend - Primary	No	No	No	No	3 & above pregnancies	didn't-junior school	didn't-junior school	Do not know	No	Moderate (debiliated daily life)		Employed/Business	Did not access	
5		daily lab/f	6-10km	Didn't attend - primary	Didn't attend - Primary	No	No	No	No	1-2 pregnancies	didn't-junior school	didn't-junior school	Community_based centres	Yes	No problem reported		Daily labor/Farmer	Did not access	
6		daily lab/f	6-10km	Secondary school	Didn't attend - Primary	Yes	Yes	No	No	1-2 pregnancies	didn't-junior school	didn't-junior school	Private clinic	Yes	No problem reported	Health complex & providers	Daily labor/Farmer	Did not access	
7		Service hc	6-10km	Didn't attend - primary	Secondary school	No	No	No	No	3 & above pregnancies	didn't-junior school	secondary & above	Secondary hospitals	Yes	No problem reported		Employed/Business	Did not access	
8	Community health w	daily lab/f	6-10km	Didn't attend - primary	Secondary school	No	No	Yes	No	3 & above pregnancies	didn't-junior school	didn't-junior school	Community_based centres	Yes	Severe (need medical attention)	Relatives & other	Daily labor/Farmer	=>4 ANC	Did not pay
9		Business	>10 km	Secondary school	Higher secondary & above	No	No	No	Yes	1-2 pregnancies	Secondary & above	secondary & above	Community_based centres	Yes	No problem reported	Relatives & other	Employed/Business	Did not access	
10		daily lab/f	>10 km	Didn't attend - primary	Didn't attend - Primary	No	No	No	No	1-2 pregnancies	didn't-junior school	didn't-junior school	Secondary hospitals	Yes	No problem reported		Daily labor/Farmer	Did not access	
11		Service hc	>10 km	Didn't attend - primary	Didn't attend - Primary	No	No	No	No	1-2 pregnancies	didn't-junior school	didn't-junior school	Private clinic	Yes	No problem reported		Employed/Business	Did not access	
12		Service hc	>10 km	Secondary school	Secondary school	No	No	No	No	1-2 pregnancies	Secondary & above	didn't-junior school	Community_based centres	Yes	Severe (need medical attention)		Employed/Business	Did not access	
13		daily lab/f	>10 km	Didn't attend - primary	Didn't attend - Primary	No	No	No	No	3 & above pregnancies	didn't-junior school	didn't-junior school	Do not know	No	Severe (need medical attention)		Daily labor/Farmer	Did not access	
14		daily lab/f	>10 km	Didn't attend - primary	Didn't attend - Primary	No	No	No	No	1-2 pregnancies	didn't-junior school	didn't-junior school	Do not know	No	No problem reported		Daily labor/Farmer	Did not access	
15		Service hc	>10 km	Didn't attend - primary	Secondary school	No	No	No	No	3 & above pregnancies	didn't-junior school	didn't-junior school	Community_based centres	Yes	Severe (need medical attention)		Employed/Business	Did not access	
16		Service hc	>10 km	Secondary school	Didn't attend - Primary	No	No	No	No	1-2 pregnancies	didn't-junior school	didn't-junior school	Community_based centres	Yes	Mild		Employed/Business	Did not access	
17		daily lab/f	>10 km	Didn't attend - primary	Didn't attend - Primary	No	No	No	No	1-2 pregnancies	didn't-junior school	didn't-junior school	Community_based centres	Yes	No problem reported		Daily labor/Farmer	Did not access	
18		Service hc	>10 km	Didn't attend - primary	Didn't attend - Primary	No	No	No	No	3 & above pregnancies	didn't-junior school	didn't-junior school	Secondary hospitals	Yes	Mild		Employed/Business	Did not access	
19		daily lab/f	>10 km	Didn't attend - primary	Didn't attend - Primary	No	Yes	No	No	3 & above pregnancies	didn't-junior school	didn't-junior school	Secondary hospitals	Yes	Severe (need medical attention)	Health complex & providers	Daily labor/Farmer	Did not access	
20		daily lab/f	>10 km	Didn't attend - primary	Didn't attend - Primary	No	Yes	No	No	3 & above pregnancies	didn't-junior school	didn't-junior school	Secondary hospitals	Yes	Severe (need medical attention)	Health complex & providers	Daily labor/Farmer	Did not access	
21		daily lab/f	>10 km	Didn't attend - primary	Didn't attend - Primary	No	Yes	No	No	3 & above pregnancies	didn't-junior school	didn't-junior school	Community_based centres	Yes	No problem reported	Health complex & providers	Daily labor/Farmer	Did not access	
22		daily lab/f	>10 km	Didn't attend - primary	Didn't attend - Primary	No	Yes	No	No	3 & above pregnancies	didn't-junior school	didn't-junior school	Secondary hospitals	Yes	Severe (need medical attention)	Health complex & providers	Daily labor/Farmer	Did not access	
23	Govt Health Complex	daily lab/f	>10 km	Didn't attend - primary	Didn't attend - Primary	No	Yes	No	No	3 & above pregnancies	didn't-junior school	didn't-junior school	Secondary hospitals	Yes	Severe (need medical attention)	Health complex & providers	Daily labor/Farmer	<4 ANC	>300 tk
24	Home visit by HW/w	daily lab/f	>10 km	Didn't attend - primary	Didn't attend - Primary	No	Yes	No	No	3 & above pregnancies	didn't-junior school	didn't-junior school	Do not know	No	Severe (need medical attention)	Health complex & providers	Daily labor/Farmer	=>4 ANC	Did not pay
25		daily lab/f	>10 km	Didn't attend - primary	Didn't attend - Primary	No	Yes	No	No	3 & above pregnancies	didn't-junior school	didn't-junior school	Secondary hospitals	Yes	No problem reported	Health complex & providers	Daily labor/Farmer	Did not access	
26	Private clinic/doctor	Business	6-10km	Higher secondary & above	Secondary school	No	Yes	No	No	1-2 pregnancies	Secondary & above	secondary & above	Community_based centres	Yes	Moderate (debiliated daily life)	Health complex & providers	Employed/Business	<4 ANC	>300 tk
27		Service hc	6-10km	Secondary school	Secondary school	No	No	Yes	No	1-2 pregnancies	didn't-junior school	secondary & above	Secondary hospitals	Yes	No problem reported	Relatives & other	Employed/Business	Did not access	
28	Govt Health Complex	Service hc	6-10km	Higher secondary & above	Didn't attend - Primary	No	Yes	No	No	1-2 pregnancies	Secondary & above	didn't-junior school	Community_based centres	Yes	No problem reported	Health complex & providers	Employed/Business	<4 ANC	<=300 tk
29		Service hc	6-10km	Didn't attend - primary	Secondary school	No	No	Yes	No	1-2 pregnancies	didn't-junior school	didn't-junior school	Community_based centres	Yes	No problem reported	Relatives & other	Employed/Business	Did not access	
30	Govt Health Complex	Service hc	6-10km	Didn't attend - primary	Didn't attend - Primary	No	No	Yes	No	1-2 pregnancies	didn't-junior school	didn't-junior school	Secondary hospitals	Yes	No problem reported	Relatives & other	Employed/Business	<4 ANC	<=300 tk
31	UHC or other priman	Service hc	6-10km	Didn't attend - primary	Didn't attend - Primary	No	No	Yes	No	1-2 pregnancies	didn't-junior school	didn't-junior school	Community_based centres	Yes	No problem reported	Relatives & other	Employed/Business	<4 ANC	>300 tk
32	Private clinic/doctor	Service hc	<5km	Secondary school	Secondary school	No	Yes	No	No	1-2 pregnancies	didn't-junior school	didn't-junior school	Secondary hospitals	Yes	No problem reported	Health complex & providers	Employed/Business	<4 ANC	<=300 tk
33		Service hc	<5km	Secondary school	Didn't attend - Primary	No	No	No	No	1-2 pregnancies	didn't-junior school	didn't-junior school	Community_based centres	Yes	No problem reported		Employed/Business	Did not access	
34		daily lab/f	<5km	Didn't attend - primary	Didn't attend - Primary	No	Yes	No	No	3 & above pregnancies	didn't-junior school	didn't-junior school	Do not know	No	No problem reported	Health complex & providers	Daily labor/Farmer	Did not access	
35		daily lab/f	<5km	Didn't attend - primary	Didn't attend - Primary	No	No	No	No	1-2 pregnancies	didn't-junior school	didn't-junior school	Community_based centres	Yes	No problem reported		Daily labor/Farmer	Did not access	
36		daily lab/f	<5km	Didn't attend - primary	Secondary school	No	No	No	No	1-2 pregnancies	didn't-junior school	didn't-junior school	Community_based centres	Yes	No problem reported		Daily labor/Farmer	Did not access	
37		Service hc	<5km	Secondary school	Secondary school	No	No	No	No	1-2 pregnancies	Secondary & above	secondary & above	Community_based centres	Yes	No problem reported		Employed/Business	Did not access	
38		daily lab/f	<5km	Secondary school	Secondary school	No	Yes	No	No	1-2 pregnancies	Secondary & above	secondary & above	Community_based centres	Yes	No problem reported	Health complex & providers	Daily labor/Farmer	Did not access	
39		daily lab/f	<5km	Secondary school	Secondary school	No	No	No	No	1-2 pregnancies	didn't-junior school	didn't-junior school	Community_based centres	Yes	No problem reported		Daily labor/Farmer	Did not access	
40		Business	<5km	Didn't attend - primary	Secondary school	No	No	No	No	1-2 pregnancies	didn't-junior school	didn't-junior school	Community_based centres	Yes	No problem reported		Employed/Business	Did not access	
41		daily lab/f	<5km	Didn't attend - primary	Didn't attend - Primary	No	No	No	No	1-2 pregnancies	didn't-junior school	didn't-junior school	Community_based centres	Yes	No problem reported		Daily labor/Farmer	Did not access	
42		daily lab/f	<5km	Didn't attend - primary	Didn't attend - Primary	No	No	Yes	No	1-2 pregnancies	didn't-junior school	didn't-junior school	Community_based centres	Yes	Severe (need medical attention)	Relatives & other	Daily labor/Farmer	Did not access	
43	Private clinic/doctor	Service hc	<5km	Didn't attend - primary	Secondary school	No	Yes	Yes	No	1-2 pregnancies	didn't-junior school	secondary & above	Community_based centres	Yes	Moderate (debiliated daily life)	Relatives & other	Employed/Business	<4 ANC	<=300 tk
44		daily lab/f	<5km	Didn't attend - primary	Didn't attend - Primary	No	No	No	No	3 & above pregnancies	didn't-junior school	didn't-junior school	Community_based centres	Yes	No problem reported		Daily labor/Farmer	Did not access	
45		Business	6-10km	Didn't attend - primary	Secondary school	Yes	Yes	No	No	1-2 pregnancies	didn't-junior school	didn't-junior school	Community_based centres	Yes	No problem reported	Health complex & providers	Employed/Business	Did not access	
46	Private clinic/doctor	daily lab/f	6-10km	Didn't attend - primary	Didn't attend - Primary	No	Yes	No	No	3 & above pregnancies	didn't-junior school	didn't-junior school	Community_based centres	Yes	Severe (need medical attention)	Health complex & providers	Daily labor/Farmer	<4 ANC	Did not pay
47		Service hc	6-10km	Secondary school	Secondary school	No	Yes	No	No	3 & above pregnancies	didn't-junior school	didn't-junior school	Community_based centres	Yes	Mild	Health complex & providers	Employed/Business	Did not access	
48	Private clinic/doctor	daily lab/f	6-10km	Secondary school	Didn't attend - Primary	No	No	No	Yes	1-2 pregnancies	Secondary & above	didn't-junior school	Community_based centres	Yes	No problem reported	Relatives & other	Daily labor/Farmer	<4 ANC	>300 tk
49	Private clinic/doctor	Service hc	6-10km	Higher secondary & above	Higher secondary & above	Yes	Yes	No	No	3 & above pregnancies	Secondary & above	secondary & above	Secondary hospitals	Yes	No problem reported	Relatives & other	Employed/Business	=>4 ANC	>300 tk
50	Community health w	daily lab/f	<5km	Higher secondary & above	Higher secondary & above	No	No	Yes	No	1-2 pregnancies	Secondary & above	secondary & above	Community_based centres	Yes	No problem reported	Relatives & other	Daily labor/Farmer	=>4 ANC	>300 tk
51		Service hc	<5km	Higher secondary & above	Higher secondary & above	No	No	No	No	1-2 pregnancies	Secondary & above	secondary & above	Community_based centres	Yes	Mild		Employed/Business	Did not access	
52		daily lab/f	<5km	Higher secondary & above	Higher secondary & above	No	No	No	No	1-2 pregnancies	Secondary & above	secondary & above	Community_based centres	Yes	Mild		Daily labor/Farmer	Did not access	
53	Private clinic/doctor	Business	<5km	Higher secondary & above	Higher secondary & above	No	Yes	No	No	3 & above pregnancies	Secondary & above	secondary & above	Secondary hospitals	Yes	Mild	Health complex & providers	Employed/Business	=>4 ANC	Did not pay
54	Private clinic/doctor	daily lab/f	<5km	Higher secondary & above	Higher secondary & above	No	Yes	Yes	No	1-2 pregnancies	Secondary & above	secondary & above	Secondary hospitals	Yes	Moderate (debiliated daily life)	Relatives & other	Daily labor/Farmer	<4 ANC	>300 tk
55	Govt Health Complex	Business	<5km	Secondary school	Didn't attend - Primary	Yes	Yes	No	No	1-2 pregnancies	didn't-junior school	didn't-junior school	Community_based centres	Yes	No problem reported	Health complex & providers	Employed/Business	=>4 ANC	Did not pay
56	UHC or other priman	Business	<5km	Secondary school	Secondary school	No	No	No	Yes	1-2 pregnancies	Secondary & above	secondary & above	Community_based centres	Yes	No problem reported	Relatives & other	Employed/Business	=>4 ANC	>300 tk
57	Private clinic/doctor	Service hc	<5km	Secondary school	Secondary school	No	Yes	No	No	1-2 pregnancies	Secondary & above	didn't-junior school	Community_based centres	Yes	No problem reported	Health complex & providers	Employed/Business	=>4 ANC	Did not pay
58	UHC or other priman	Service hc	<5km	Higher secondary & above	Higher secondary & above	No	No	Yes	No	1-2 pregnancies	Secondary & above	secondary & above	Community_based centres	Yes	No problem reported	Relatives & other	Employed/Business	<4 ANC	>300 tk
59	UHC or other priman	daily lab/f	<5km	Didn't attend - primary	Secondary school	No	Yes	No	No	1-2 pregnancies	didn't-junior school	didn't-junior school	Community_based centres	Yes	No problem reported	Health complex & providers	Daily labor/Farmer	<4 ANC	<=300 tk
60	Community health w	daily lab/f	<5km	Secondary school	Didn't attend - Primary	No	Yes	No	No	1-2 pregnancies	didn't-junior school	didn't-junior school	Community_based centres	Yes	No problem reported	Health complex & providers	Daily labor/Farmer	<4 ANC	Did not pay
61		daily lab/f	<5km	Didn't attend - primary	Didn't attend - Primary	No	No	No	No	3 & above pregnancies	didn't-junior school	didn't-junior school	Community_based centres	Yes	No problem reported		Daily labor/Farmer	Did not access	
62	Private clinic/doctor	Business	<5km	Didn't attend - primary	Didn't attend - Primary	No	Yes	No	No	3 & above pregnancies	didn't-junior school	didn't-junior school	Community_based centres	Yes	Severe (need medical attention)	Health complex & providers	Employed/Business	<4 ANC	<=300 tk
63		daily lab/f	6-10km	Didn't attend - primary	Didn't attend - Primary	No	No	No	No	3 & above pregnancies	didn't-junior school	didn't-junior school	Community_based centres	Yes	No problem reported		Daily labor/Farmer	Did not access	
64	Private clinic/doctor	daily lab/f	6-10km	Didn't attend - primary	Didn't attend - Primary	No	Yes	No	No	3 & above pregnancies	didn't-junior school	didn't-junior school	Community_based centres	Yes	Severe (need medical attention)	Health complex & providers	Daily labor/Farmer	<4 ANC	Did not pay
65	UHC or other priman	daily lab/f	6-10km	Didn't attend - primary	Didn't attend - Primary	No	Yes	No	No	3 & above pregnancies	didn't-junior school	didn't-junior school	Community_based centres	Yes	Severe (need medical attention)	Health complex & providers	Daily labor/Farmer	<4 ANC	Did not pay
66		daily lab/f	6-10km	Secondary school	Secondary school	No	No	No	No	1-2 pregnancies	didn't-junior school	didn't-junior school	Community_based centres	Yes	No problem reported		Daily labor/Farmer	Did not access	
67		daily lab/f	6-10km	Secondary school	Higher secondary & above	No	No	Yes	No	1-2 pregnancies	didn't-junior school	secondary & above	Community_based centres	Yes	No problem reported	Relatives & other	Daily labor/Farmer	Did not access	
68		daily lab/f	6-10km	Secondary school	Didn't attend - Primary	No	No	No	No	1-2 pregnancies	Secondary & above	didn't-junior school	Community_based centres	Yes	No problem reported		Daily labor/Farmer	Did not access	
69		daily lab/f	6-10km	Secondary school	Didn't attend - Primary	No	No	No	No	3 & above pregnancies	didn't-junior school	didn't-junior school	Community_based centres	Yes	No problem reported		Daily labor/Farmer	Did not access	
70		daily lab/f	6-10km	Didn't attend - primary	Didn't attend - Primary	No	No	No	No	3 & above pregnancies	didn't-junior school	didn't-junior school	Community_based centres	Yes	No problem reported		Daily labor/Farmer	Did not access	
71	Private clinic/doctor	Business	6-10km	Didn't attend - primary	Secondary school	No	Yes	No	No	1-2 pregnancies	didn't-junior school	didn't-junior school	Community_based centres	Yes	No problem reported	Health complex & providers	Employed/Business	<4 ANC	Did not pay
72		daily lab/f	6-10km	Didn't attend - primary	Secondary school	No	Yes	No	No	1-2 pregnancies	didn't-junior school	didn't-junior school	Community_based centres	Yes	No problem reported	Health complex & providers	Daily labor/Farmer	Did not access	
73		daily lab/f	6-10km	Secondary school	Didn't attend - Primary	No	No	No	No	1-2 pregnancies	didn't-junior school	didn't-junior school	Do not know	No	No problem reported		Daily labor/Farmer	Did not access	
74		Service hc	6-10km	Didn't attend - primary	Secondary school	No	No	No	No	1-2 pregnancies	didn't-junior school	didn't-junior school	Community_based centres	Yes	No problem reported		Employed/Business	Did not access	
75		daily lab/f	6-10km	Didn't attend - primary	Secondary school	No	No	No	No	3 & above pregnancies	didn't-junior school	didn't-junior school	Community_based centres	Yes	No problem reported		Daily labor/Farmer	Did not access	
76	UHC or other priman	daily lab/f	6-10km	Secondary school	Secondary school	No	Yes	No	No	1-2 pregnancies	Secondary & above	secondary & above	Secondary hospitals	Yes	Mild	Health complex & providers	Daily labor/Farmer	=>4 ANC	Did not pay
77	Private clinic/doctor	daily lab/f	6-10km	Secondary school	Didn't attend - Primary	No	Yes	No	No	3 & above pregnancies	didn't-junior school	didn't-junior school	Community_based centres	Yes	Severe (need medical attention)	Health complex & providers	Daily labor/Farmer	<4 ANC	<=300 tk
78	Private clinic/doctor	Service hc	6-10km	Secondary school	Didn't attend - Primary	No	Yes	No	No	3 & above pregnancies	didn't-junior school	didn't-junior school	Community_based centres	Yes	No problem reported	Health complex & providers	Daily labor/Farmer	<4 ANC	<=300 tk
79		Service hc	6-10km	Secondary school	Secondary school	No	No	No	No	1-2 pregnancies	didn't-junior school	secondary & above	Community_based centres	Yes	No problem reported		Employed/Business	Did not access	
80		daily lab/f	<5km	Didn't attend - primary	Didn't attend - Primary	No	No	No	No	1-2 pregnancies	didn't-junior school	didn't-junior school	Community_based centres	Yes	No problem reported		Daily labor/Farmer	Did not access	
81		daily lab/f	<5km	Higher secondary & above	Higher secondary & above	Yes	No	No	No	1-2 pregnancies	Secondary & above	secondary & above	Community_based centres	Yes	No problem reported	Health complex & providers	Daily labor/Farmer	Did not access	
82		daily lab/f	<5km	Secondary school	Secondary school	No	No	No	No	1-2 pregnancies	Secondary & above	secondary & above	Community_based centres	Yes	No problem reported		Daily labor/Farmer	Did not access	
83		daily lab/f	<5km	Didn't attend - primary	Didn't attend - Primary	No	No	No	No	1-2 pregnancies	didn't-junior school	didn't-junior school	Community_based centres	Yes	No problem reported		Daily labor/Farmer	Did not access	
84		daily lab/f	<5km	Secondary school	Didn't attend - Primary	No	No	No	No	1-2 pregnancies	didn't-junior school	didn't-junior school	Community_based centres	Yes	No problem reported		Daily labor/Farmer	Did not access	
85	Private clinic/doctor	daily lab/f	<5km	Higher secondary & above	Higher secondary & above	Yes	Yes	No	No	1-2 pregnancies	Secondary & above	secondary & above	Community_based centres	Yes	No problem reported	Health complex & providers	Daily labor/Farmer	<4 ANC	>300 tk
86	Community health w	daily lab/f	<5km	Higher secondary & above	Higher secondary & above	Yes	Yes	No	No	1-2 pregnancies	Secondary & above	secondary & above	Private clinic	Yes	Mild	Health complex & providers	Daily labor/Farmer	<4 ANC	>300 tk
87	UHC or other priman	Business	<5km	Secondary school	Secondary school	No	Yes	No	No	1-2 pregnancies	Secondary & above	secondary & above	Community_based centres	Yes	No problem reported	Relatives & other	Employed/Business	=>4 ANC	Did not pay
88	UHC or other priman	Service hc	6-10km	Secondary school	Higher secondary & above	No	Yes	No	No	1-2 pregnancies	Secondary & above	secondary & above	Private clinic	Yes	No problem reported	Health complex & providers	Employed/Business	=>4 ANC	>300 tk
89		daily lab/f	6-10km	Didn't attend - primary	Didn't attend - Primary	No	No	No	No	1-2 pregnancies	didn't-junior school	didn't-junior school	Community_based centres	Yes	No problem reported		Daily labor/Farmer	Did not access	
90	Private clinic/doctor	Service hc	6-10km	Secondary school	Secondary school	No	Yes	No	No	1-2 pregnancies	didn't-junior school	didn't-junior school	Community_based centres	Yes	Mild	Health complex & providers	Employed/Business	<4 ANC	>300 tk
91		Service hc	6-10km	Secondary school	Secondary school	No	Yes	Yes	No	1-2 pregnancies	didn't-junior school	secondary & above	Community_based centres	Yes	No problem reported	Relatives & other	Employed/Business	Did not access	
92	Community health w	Service hc	6-10km	Secondary school	Secondary school	No	Yes	No	No	1-2 pregnancies	Secondary & above	secondary & above	Community_based centres	Yes	Mild	Health complex & providers	Employed/Business	<4 ANC	<=300 tk
93		Service hc	6-10km	Didn't attend - primary	Secondary school	No	No	No	No	1-2 pregnancies	didn't-junior school	didn't-junior school	Community_based centres	Yes	No problem reported		Employed/Business	Did not access	
94	Community health w	Service hc	6-10km	Secondary school	Didn't attend - Primary	No	Yes	No	No	1-2 pregnancies	didn't-junior school	didn't-junior school	Community_based centres	Yes	No problem reported	Health complex & providers	Employed/Business	<4 ANC	Did not pay
95		daily lab/f	6-10km	Didn't attend - primary	Didn't attend - Primary	No	No	No	No	1-2 pregnancies	didn't-junior school	didn't-junior school	Community_based centres	Yes	No problem reported		Daily labor/Farmer	Did not access	
96		Business	6-10km	Didn't attend - primary	Secondary school	No	No	No	No	3 & above pregnancies	didn't-junior school	didn't-junior school	Community_based centres	Yes	Mild		Employed/Business	Did not access	
97	Private clinic/doctor	daily lab/f	6-10km	Secondary school	Secondary school	No	Yes	No	No	1-2 pregnancies	Secondary & above	didn't-junior school	Secondary hospitals	Yes	Mild	Health complex & providers	Daily labor/Farmer	<4 ANC	Did not pay
98		daily lab/f	6-10km	Didn't attend - primary	Didn't attend - Primary	No	No	No	No	1-2 pregnancies	didn't-junior school	didn't-junior school	Community_based centres	Yes	Mild		Daily labor/Farmer	Did not access	
99	Community health w	daily lab/f	6-10km	Didn't attend - primary	Didn't attend - Primary	No	Yes	No	No	1-2 pregnancies	didn't-junior school	didn't-junior school	Community_based centres	Yes	No problem reported	Health complex & providers	Daily labor/Farmer	=>4 ANC	Did not pay
100	Private clinic/doctor	Service hc	6-10km	Didn't attend - primary	Didn't attend - Primary	No	Yes	No	No	1-2 pregnancies	didn't-junior school	didn't-junior school	Community_based centres	Yes	No problem reported	Health complex & providers	Employed/Business	<4 ANC	Did not pay
101	Private clinic/doctor	daily lab/f	6-10km	Secondary school	Higher secondary & above	No	No	Yes	No	1-2 pregnancies	Secondary & above	secondary & above	Community_based centres	Yes	No problem reported	Relatives & other	Daily labor/Farmer	<4 ANC	>300 tk
102	Community health w	Service hc	6-10km	Higher secondary & above	Higher secondary & above	No	Yes	No	No	1-2 pregnancies	Secondary & above	secondary & above	Community_based centres	Yes	No problem reported	Health complex & providers	Employed/Business	=>4 ANC	>300 tk
103	Community health w	Service hc	6-10km	Secondary school	Didn't attend - Primary	Yes	No	No	No	1-2 pregnancies	didn't-junior school	didn't-junior school	Private clinic	Yes	Moderate (debiliated daily life)	Health complex & providers	Employed/Business	<4 ANC	Did not pay
104	Private clinic/doctor	Service hc	6-10km	Secondary school	Didn't attend - Primary	No	Yes	No	No	3 & above pregnancies	didn't-junior school	didn't-junior school	Community_based centres	Yes	Severe (need medical attention)	Health complex & providers	Employed/Business	<4 ANC	<=300 tk
105	Private clinic/doctor	daily lab/f	6-10km	Didn't attend - primary	Didn't attend - Primary	No	Yes	No	No	1-2 pregnancies	didn't-junior school	didn't-junior school	Community_based centres	Yes	Mild	Health complex & providers	Daily labor/Farmer	<4 ANC	<=300 tk
106		daily lab/f	6-10km	Didn't attend - primary	Didn't attend - Primary	No	Yes	No	No	3 & above pregnancies	didn't-junior school	didn't-junior school	Community_based centres	Yes	Severe (need medical attention)	Health complex & providers	Daily labor/Farmer	Did not access	
107		daily lab/f	6-10km	Didn't attend - primary	Didn't attend - Primary	No	No	No	No	3 & above pregnancies	didn't-junior school	didn't-junior school	Community_based centres	Yes	No problem reported		Daily labor/Farmer	Did not access	
108		daily lab/f	6-10km	Didn't attend - primary	Didn't attend - Primary	No	No	No	No	1-2 pregnancies	didn't-junior school	didn't-junior school	Community_based centres	Yes	No problem reported		Daily labor/Farmer	Did not access	
109		daily lab/f	6-10km	Secondary school	Didn't attend - Primary	No	Yes	No	No	1-2 pregnancies	didn't-junior school	didn't-junior school	Community_based centres	Yes	Mild	Health complex & providers	Daily labor/Farmer	Did not access	
110		Service hc	6-10km	Didn't attend - primary	Didn't attend - Primary	No	No	No	No	1-2 pregnancies	didn't-junior school	didn't-junior school	Secondary hospitals	Yes	No problem reported		Employed/Business	Did not access	
111		daily lab/f	6-10km	Didn't attend - primary	Didn't attend - Primary	No	No	No	No	3 & above pregnancies	didn't-junior school	didn't-junior school	Community_based centres	Yes	Severe (need medical attention)		Daily labor/Farmer	Did not access	
112		Business	6-10km	Didn't attend - primary	Didn't attend - Primary	No	Yes	No	No	1-2 pregnancies	didn't-junior school	didn't-junior school	Community_based centres	Yes	Severe (need medical attention)	Health complex & providers	Employed/Business	Did not access	
113		Service hc	6-10km	Secondary school	Didn't attend - Primary	No	No	No	No	1-2 pregnancies	didn't-junior school	didn't-junior school	Community_based centres	Yes	No problem reported		Employed/Business	Did not access	
114		daily lab/f	6-10km	Didn't attend - primary	Didn't attend - Primary	No	No	No	No	3 & above pregnancies	didn't-junior school	didn't-junior school	Do not know	No	Severe (need medical attention)		Daily labor/Farmer	Did not access	
115		Service hc	6-10km	Secondary school	Didn't attend - Primary	No	No	No	No	1-2 pregnancies	didn't-junior school	didn't-junior school	Community_based centres	Yes	Mild		Employed/Business	Did not access	
116	Private clinic/doctor	daily lab/f	6-10km	Didn't attend - primary	Didn't attend - Primary	No	Yes	No	No	1-2 pregnancies	didn't-junior school	didn't-junior school	Community_based centres	Yes	Mild	Health complex & providers	Daily labor/Farmer	<4 ANC	Did not pay
117		Service hc	6-10km	Secondary school	Didn't attend - Primary	No	No	Yes	No	1-2 pregnancies	didn't-junior school	didn't-junior school	Community_based centres	Yes	Severe (need medical attention)	Relatives & other	Employed/Business	Did not access	
118		daily lab/f	6-10km	Didn't attend - primary	Didn't attend - Primary	No	No	Yes	No	3 & above pregnancies	didn't-junior school	didn't-junior school	Community_based centres	Yes	Mild	Relatives & other	Daily labor/Farmer	Did not access	
119		Service hc	6-10km	Secondary school	Didn't attend - Primary	No	No	Yes	No	1-2 pregnancies	didn't-junior school	didn't-junior school	Community_based centres	Yes	Severe (need medical attention)	Relatives & other	Employed/Business	Did not access	
120	UHC or other priman	daily lab/f	6-10km	Secondary school	Didn't attend - Primary	No	Yes	No	No	1-2 pregnancies	Secondary & above	didn't-junior school	Secondary hospitals	Yes	No problem reported	Health complex & providers	Daily labor/Farmer	<4 ANC	Did not pay
121	UHC or other priman	daily lab/f	6-10km	Higher secondary & above	Higher secondary & above	No	Yes	No	No	1-2 pregnancies	Secondary & above	secondary & above	Private clinic	Yes	Mild	Health complex & providers	Daily labor/Farmer	=>4 ANC	Did not pay
122		Service hc	6-10km	Didn't attend - primary	Secondary school	No	No	No	No	1-2 pregnancies	didn't-junior school	didn't-junior school	Community_based centres	Yes	No problem reported		Employed/Business	Did not access	
123		Service hc	6-10km	Didn't attend - primary	Secondary school	No	No	No	No	1-2 pregnancies	didn't-junior school	secondary & above	Community_based centres	Yes	No problem reported		Employed/Business	Did not access	
124	UHC or other priman	Service hc	6-10km	Didn't attend - primary	Didn't attend - Primary	No	Yes	No	No	1-2 pregnancies	didn't-junior school	didn't-junior school	Community_based centres	Yes	Mild	Health complex & providers	Employed/Business	<4 ANC	Did not pay
125	Private clinic/doctor	Service hc	6-10km	Higher secondary & above	Higher secondary & above	Yes	No	No	No	1-2 pregnancies	Secondary & above	secondary & above	Community_based centres	Yes	No problem reported	Health complex & providers	Employed/Business	<4 ANC	>300 tk
126	Community health w	Service hc	6-10km	Secondary school	Secondary school	No	Yes	No	No	1-2 pregnancies	didn't-junior school	secondary & above	Secondary hospitals	Yes	Severe (need medical attention)	Health complex & providers	Employed/Business	<4 ANC	<=300 tk
127		daily lab/f	6-10km	Didn't attend - primary	Didn't attend - Primary	No	No	No	No	1-2 pregnancies	didn't-junior school	didn't-junior school	Community_based centres	Yes	No problem reported		Daily labor/Farmer	Did not access	
128		Business	6-10km	Didn't attend - primary	Secondary school	No	No	No	No	3 & above pregnancies	didn't-junior school	didn't-junior school	Community_based centres	Yes	No problem reported		Employed/Business	Did not access	
129		daily lab/f	6-10km	Didn't attend - primary	Didn't attend - Primary	No	No	No	No	3 & above pregnancies	didn't-junior school	didn't-junior school	Do not know	No	No problem reported		Daily labor/Farmer	Did not access	
130		Service hc	6-10km	Secondary school	Secondary school	No	Yes	No	No	1-2 pregnancies	Secondary & above	secondary & above	Secondary hospitals	Yes	No problem reported	Health complex & providers	Employed/Business	Did not access	
131		Service hc	6-10km	Didn't attend - primary	Secondary school	No	No	No	No	3 & above pregnancies	didn't-junior school	didn't-junior school	Community_based centres	Yes	Mild		Employed/Business	Did not access	
132	UHC or other priman	Service hc	6-10km	Higher secondary & above	Higher secondary & above	No	Yes	No	No	1-2 pregnancies	Secondary & above	secondary & above	Community_based centres	Yes	Severe (need medical attention)	Health complex & providers	Employed/Business	<4 ANC	Did not pay
133	UHC or other priman	Service hc	6-10km	Secondary school	Secondary school	No	Yes	No	No	1-2 pregnancies	Secondary & above	didn't-junior school	Community_based centres	Yes	Moderate (debiliated daily life)	Health complex & providers	Employed/Business	<4 ANC	Did not pay
134	UHC or other priman	daily lab/f	6-10km	Didn't attend - primary	Didn't attend - Primary	No	Yes	No	No	3 & above pregnancies	didn't-junior school	didn't-junior school	Do not know	No	Severe (need medical attention)	Health complex & providers	Daily labor/Farmer	<4 ANC	Did not pay
135	UHC or other priman	Service hc	6-10km	Secondary school	Secondary school	Yes	No	No	No	1-2 pregnancies	Secondary & above	secondary & above	Private clinic	Yes	Mild	Health complex & providers	Employed/Business	=>4 ANC	Did not pay
136	Private clinic/doctor	Business	6-10km	Secondary school	Secondary school	No	Yes	Yes	No	1-2 pregnancies	didn't-junior school	didn't-junior school	Secondary hospitals	Yes	No problem reported	Relatives & other	Employed/Business	<4 ANC	>300 tk
137	Private clinic/doctor	Service hc	6-10km	Higher secondary & above	Secondary school	No	Yes	No	No	1-2 pregnancies	Secondary & above	secondary & above	Secondary hospitals	Yes	Severe (need medical attention)	Health complex & providers	Employed/Business	<4 ANC	>300 tk
138	Private clinic/doctor	daily lab/f	<5km	Higher secondary & above	Higher secondary & above	No	Yes	No	No	1-2 pregnancies	Secondary & above	secondary & above	Private clinic	Yes	No problem reported	Health complex & providers	Daily labor/Farmer	=>4 ANC	>300 tk
139	Private clinic/doctor	daily lab/f	<5km	Higher secondary & above	Higher secondary & above	No	Yes	No	No	1-2 pregnancies	Secondary & above	secondary & above	Private clinic	Yes	No problem reported	Health complex & providers	Daily labor/Farmer	=>4 ANC	>300 tk
140		daily lab/f	<5km	Didn't attend - primary	Didn't attend - Primary	No	No	No	No	3 & above pregnancies	didn't-junior school	didn't-junior school	Community_based centres	Yes	Mild		Daily labor/Farmer	Did not access	
141		daily lab/f	<5km	Secondary school	Secondary school	No	No	No	No	1-2 pregnancies	Secondary & above	didn't-junior school	Private clinic	Yes	Severe (need medical attention)		Daily labor/Farmer	Did not access	
142	Community health w	daily lab/f	<5km	Higher secondary & above	Secondary school	No	No	Yes	No	1-2 pregnancies	Secondary & above	secondary & above	Community_based centres	Yes	Severe (need medical attention)	Relatives & other	Daily labor/Farmer	<4 ANC	>300 tk
143		Service hc	<5km	Secondary school	Secondary school	No	No	No	No	1-2 pregnancies	didn't-junior school	secondary & above	Community_based centres	Yes	No problem reported		Employed/Business	Did not access	
144	Private clinic/doctor	Service hc	<5km	Secondary school	Higher secondary & above	No	No	Yes	No	1-2 pregnancies	Secondary & above	secondary & above	Private clinic	Yes	Severe (need medical attention)	Relatives & other	Employed/Business	=>4 ANC	>300 tk
145	Private clinic/doctor	daily lab/f	<5km	Higher secondary & above	Secondary school	Yes	Yes	No	No	1-2 pregnancies	Secondary & above	didn't-junior school	Private clinic	Yes	No problem reported	Health complex & providers	Daily labor/Farmer	=>4 ANC	>300 tk
146	Private clinic/doctor	daily lab/f	<5km	Higher secondary & above	Higher secondary & above	No	Yes	No	No	1-2 pregnancies	Secondary & above	secondary & above	Private clinic	Yes	Mild	Health complex & providers	Daily labor/Farmer	<4 ANC	>300 tk
147	Private clinic/doctor	Service hc	<5km	Secondary school	Secondary school	Yes	No	No	No	1-2 pregnancies	didn't-junior school	secondary & above	Private clinic	Yes	Severe (need medical attention)	Health complex & providers	Employed/Business	=>4 ANC	>300 tk
148	Private clinic/doctor	daily lab/f	<5km	Didn't attend - primary	Didn't attend - Primary	Yes	No	No	No	3 & above pregnancies	didn't-junior school	didn't-junior school	Private clinic	Yes	Mild	Health complex & providers	Daily labor/Farmer	<4 ANC	>300 tk
149	Community health w	daily lab/f	<5km	Secondary school	Secondary school	No	Yes	No	No	3 & above pregnancies	didn't-junior school	didn't-junior school	Community_based centres	Yes	No problem reported	Health complex & providers	Daily labor/Farmer	<4 ANC	>300 tk
150	Private clinic/doctor	daily lab/f	<5km	Didn't attend - primary	Secondary school	Yes	Yes	No	No	3 & above pregnancies	didn't-junior school	didn't-junior school	Private clinic	Yes	No problem reported	Health complex & providers	Daily labor/Farmer	<4 ANC	>300 tk
151	Private clinic/doctor	Service hc	<5km	Secondary school	Secondary school	Yes	Yes	No	No	1-2 pregnancies	Secondary & above	secondary & above	Private clinic	Yes	Mild	Health complex & providers	Employed/Business	<4 ANC	Did not pay
152	Private clinic/doctor	Business	<5km	Higher secondary & above	Secondary school	Yes	No	Yes	No	1-2 pregnancies	Secondary & above	secondary & above	Private clinic	Yes	Mild	Relatives & other	Employed/Business	<4 ANC	<=300 tk
153	Private clinic/doctor	Service hc	<5km	Secondary school	Higher secondary & above	No	Yes	No	No	1-2 pregnancies	Secondary & above	secondary & above	Private clinic	Yes	Severe (need medical attention)	Relatives & other	Employed/Business	<4 ANC	>300 tk
154	Private clinic/doctor	daily lab/f	<5km	Secondary school	Higher secondary & above	No	No	Yes	No	3 & above pregnancies	Secondary & above	secondary & above	Private clinic	Yes	Mild	Relatives & other	Daily labor/Farmer	<4 ANC	>300 tk
155		Service hc	<5km	Secondary school	Secondary school	No	No	Yes	No	1-2 pregnancies	Secondary & above	secondary & above	Private clinic	Yes	Mild	Relatives & other	Employed/Business	Did not access	
156		daily lab/f	6-10km	Didn't attend - primary	Secondary school	No	No	No	No	3 & above pregnancies	didn't-junior school	didn't-junior school	Secondary hospitals	Yes	No problem reported		Daily labor/Farmer	Did not access	
157		daily lab/f	6-10km	Didn't attend - primary	Didn't attend - Primary	No	No	No	No	3 & above pregnancies	didn't-junior school	didn't-junior school	Do not know	No	Mild		Daily labor/Farmer	Did not access	
158	UHC or other priman	Service hc	6-10km	Higher secondary & above	Higher secondary & above	Yes	Yes	No	No	1-2 pregnancies	Secondary & above	secondary & above	Private clinic	Yes	Mild	Health complex & providers	Employed/Business	<4 ANC	>300 tk
159		Service hc	6-10km	Secondary school	Secondary school	No	Yes	No	No	3 & above pregnancies	Secondary & above	didn't-junior school	Private clinic	Yes	Severe (need medical attention)	Health complex & providers	Employed/Business	Did not access	
160	Govt Health Complex	daily lab/f	6-10km	Secondary school	Secondary school	Yes	Yes	No	No	3 & above pregnancies	Secondary & above	secondary & above	Private clinic	Yes	Mild	Relatives & other	Daily labor/Farmer	<4 ANC	Did not pay
161	Home visit by HW/w	daily lab/f	6-10km	Secondary school	Secondary school	No	Yes	Yes	No	1-2 pregnancies	Secondary & above	didn't-junior school	Community_based centres	Yes	Mild	Relatives & other	Daily labor/Farmer	<4 ANC	Did not pay
162	Private clinic/doctor	Business	<5km	Higher secondary & above	Didn't attend - Primary	Yes	Yes	No	No	1-2 pregnancies	Secondary & above	didn't-junior school	Private clinic	Yes	Severe (need medical attention)	Health complex & providers	Employed/Business	<4 ANC	>300 tk
163	Private clinic/doctor	Business	<5km	Secondary school	Secondary school	Yes	Yes	No	No	1-2 pregnancies	Secondary & above	secondary & above	Private clinic	Yes	Mild	Health complex & providers	Employed/Business	<4 ANC	>300 tk
164	Community health w	daily lab/f	6-10km	Higher secondary & above	Higher secondary & above	Yes	No	Yes	No	1-2 pregnancies	Secondary & above	secondary & above	Private clinic	Yes	No problem reported	Relatives & other	Daily labor/Farmer	=>4 ANC	Did not pay
165	Private clinic/doctor	Service hc	6-10km	Secondary school	Higher secondary & above	No	Yes	No	No	3 & above pregnancies	Secondary & above	secondary & above	Private clinic	Yes	No problem reported	Health complex & providers	Employed/Business	<4 ANC	>300 tk
166	Private clinic/doctor	daily lab/f	6-10km	Didn't attend - primary	Didn't attend - Primary	No	No	Yes	No	1-2 pregnancies	didn't-junior school	didn't-junior school	Private clinic	Yes	No problem reported	Relatives & other	Daily labor/Farmer	<4 ANC	>300 tk
167	Govt Health Complex	daily lab/f	6-10km	Secondary school	Secondary school	No	Yes	No	No	1-2 pregnancies	didn't-junior school	secondary & above	Private clinic	Yes	No problem reported	Health complex & providers	Daily labor/Farmer	<4 ANC	Did not pay
168	Private clinic/doctor	daily lab/f	6-10km	Higher secondary & above	Higher secondary & above	No	Yes	No	No	1-2 pregnancies	Secondary & above	secondary & above	Private clinic	Yes	Severe (need medical attention)	Health complex & providers	Daily labor/Farmer	<4 ANC	>300 tk
169	Private clinic/doctor	Business	6-10km	Higher secondary & above	Higher secondary & above	No	No	Yes	No	1-2 pregnancies	Secondary & above	secondary & above	Private clinic	Yes	No problem reported	Relatives & other	Employed/Business	=>4 ANC	Did not pay
170	Private clinic/doctor	daily lab/f	6-10km	Higher secondary & above	Higher secondary & above	No	Yes	No	No	1-2 pregnancies	Secondary & above	secondary & above	Private clinic	Yes	Mild	Health complex & providers	Daily labor/Farmer	=>4 ANC	Did not pay
171	Private clinic/doctor	Service hc	6-10km	Higher secondary & above	Higher secondary & above	Yes	No	No	No	3 & above pregnancies	Secondary & above	secondary & above	Private clinic	Yes	Severe (need medical attention)	Relatives & other	Employed/Business	=>4 ANC	>300 tk
172	Private clinic/doctor	Service hc	<5km	Higher secondary & above	Higher secondary & above	No	No	Yes	No	1-2 pregnancies	Secondary & above	secondary & above	Private clinic	Yes	Mild	Relatives & other	Employed/Business	=>4 ANC	>300 tk
173	Govt Health Complex	Service hc	<5km	Higher secondary & above	Secondary school	No	No	Yes	No	1-2 pregnancies	Secondary & above	secondary & above	Private clinic	Yes	Mild	Relatives & other	Employed/Business	<4 ANC	Did not pay
174	Private clinic/doctor	daily lab/f	6-10km	Secondary school	Higher secondary & above	No	Yes	No	No	1-2 pregnancies	Secondary & above	secondary & above	Private clinic	Yes	Mild	Health complex & providers	Daily labor/Farmer	=>4 ANC	>300 tk
175	Private clinic/doctor	Service hc	6-10km	Higher secondary & above	Higher secondary & above	No	No	Yes	No	1-2 pregnancies	Secondary & above	secondary & above	Private clinic	Yes	Mild	Relatives & other	Employed/Business	<4 ANC	>300 tk

176	25	Khagrachhari s	=>30	Marma	Buddhist	Yes	No	Yes	Yes	Yes	Yes	No	Yes	Your own language		Adult (30 & above v	became pregnant 20+ yrs	Yes	4000-9000	Illness/complications	Complete housewife	2nd trimesters	No	Govt Health Comple	daily lab/f	6-10km	Secondary school	Secondary school	No	Yes	Yes	No	3 & above pregnancies	Secondary & above	didn't-junior school	Private clinic	Yes	Mild	Relatives & other	Daily labor/Farmer	<4 ANC	Did not pay
177	25	Khagrachhari s	25-29	Marma	Buddhist	No	No	Yes	Yes	Yes	Yes	No	No	Bangla	Yes	Young (15-29 yrs)	became pregnant 20+ yrs	Yes	20000 and above	Confirm pregnancy	Complete housewife	1st trimesters	Yes	Govt Health Comple	daily lab/f	6-10km	Higher secondary & above	Higher secondary & above	Yes	No	Yes	No	1-2 pregnancies	Secondary & above	secondary & above	Private clinic	Yes	Mild	Relatives & other	Daily labor/Farmer	=>4 ANC	Did not pay
178	25	Khagrachhari s	=>30	Marma	Buddhist	No	Yes	No	Yes	Yes	Yes	No	Yes	Bangla	Yes	Adult (30 & above v	became pregnant 20+ yrs	Yes	20000 and above	Foetus sex	Complete housewife	2nd trimesters	Yes	Govt Health Comple	daily lab/f	6-10km	Higher secondary & above	Higher secondary & above	No	Yes	No	No	1-2 pregnancies	Secondary & above	secondary & above	Private clinic	Yes	No problem reported	Health complex & providers	Daily labor/Farmer	=>4 ANC	Did not pay
179	25	Khagrachhari s	=>30	Marma	Buddhist	No	No	No	Yes	Yes	Yes	No	No	Bangla	Yes	Adult (30 & above v	became pregnant 20+ yrs	Yes	20000 and above	Confirm pregnancy	Involve in income generation	1st trimesters	Yes	Private clinic/doctor	daily lab/f	6-10km	Higher secondary & above	Higher secondary & above	Yes	Yes	No	No	1-2 pregnancies	Secondary & above	secondary & above	Private clinic	Yes	No problem reported	Health complex & providers	Daily labor/Farmer	=>4 ANC	Did not pay
180	25	Khagrachhari s	=>30	Marma	Buddhist	No	No	Yes	Yes	Yes	Yes	Yes	No	Bangla	Yes	Adult (30 & above v	became pregnant 20+ yrs	Yes	20000 and above	Confirm pregnancy	Involve in income generation	1st trimesters	Yes	Private clinic/doctor	daily lab/f	6-10km	Higher secondary & above	Higher secondary & above	Yes	Yes	No	No	1-2 pregnancies	Secondary & above	secondary & above	Private clinic	Yes	Mild	Relatives & other	Daily labor/Farmer	=>4 ANC	>300 tk
181	26	Khagrachhari s	=>30	Chakma	Buddhist	No	Yes	Yes	Yes	Yes	Yes	Yes	Yes	Your own language		Adult (30 & above v	became pregnant 20+ yrs	Yes	20000 and above	Confirm pregnancy	Complete housewife	1st trimesters	Yes	Private clinic/doctor	Business	<5km	Higher secondary & above	Higher secondary & above	Yes	Yes	No	No	1-2 pregnancies	Secondary & above	secondary & above	Private clinic	Yes	Mild	Health complex & providers	Employed/Business	<4 ANC	>300 tk
182	26	Khagrachhari s	16-24	Tripura	Sonatoni	No	No	Yes	Yes	Don't kno	Yes	Yes	No	Bangla	No	Young (15-29 yrs)	became preg <19	Yes	4000-9000	Foetus sex	Complete housewife	3rd trimesters	No	Private clinic/doctor	daily lab/f	<5km	Secondary school	Didn't attend - Primary	Yes	No	No	No	1-2 pregnancies	didn't-junior school	didn't-junior school	Community_based centres	Yes	Severe (need medical attention)	Health complex & providers	Daily labor/Farmer	<4 ANC	>300 tk
183	26	Khagrachhari s	16-24	Chakma	Buddhist	No	No	Yes	Yes	Don't kno	No					Young (15-29 yrs)	became preg <19	Yes	4000-9000		Involve in income generation		No		daily lab/f	<5km	Secondary school	Didn't attend - Primary	No	Yes	No	No	1-2 pregnancies	Secondary & above	didn't-junior school	Private clinic	Yes	Mild	Health complex & providers	Daily labor/Farmer	Did not access	
184	26	Khagrachhari s	25-29	Tripura	Sonatoni	No	No	No	No							Young (15-29 yrs)	became preg <19	No	10000-19000		Involve in income generation		No		daily lab/f	<5km	Didn't attend - primary	Didn't attend - Primary	No	No	No	No	1-2 pregnancies	didn't-junior school	didn't-junior school	Do not know	No	No problem reported		Daily labor/Farmer	Did not access	
185	26	Khagrachhari s	16-24	Tripura	Sonatoni	No	No	Yes	No							Young (15-29 yrs)	became preg <19	Yes	4000-9000		Involve in income generation		No		daily lab/f	<5km	Didn't attend - primary	Didn't attend - Primary	No	No	No	No	1-2 pregnancies	didn't-junior school	didn't-junior school	Community_based centres	Yes	Severe (need medical attention)		Daily labor/Farmer	Did not access	
186	26	Khagrachhari s	=>30	Chakma	Buddhist	No	No	No	Yes	Don't kno	Yes	No	No	Bangla	No	Adult (30 & above v	became preg <19	Yes	10000-19000	other	Involve in income generation	2nd trimesters	No	Private clinic/doctor	daily lab/f	<5km	Didn't attend - primary	Didn't attend - Primary	No	Yes	No	No	3 & above pregnancies	didn't-junior school	didn't-junior school	Private clinic	Yes	No problem reported	Health complex & providers	Daily labor/Farmer	<4 ANC	Did not pay
187	26	Khagrachhari s	16-24	Chakma	Buddhist	No	No	No	Yes	Yes	Yes	Yes	No	Bangla	No	Young (15-29 yrs)	became preg <19	Yes	20000 and above	Confirm pregnancy	Involve in income generation	1st trimesters	No	Private clinic/doctor	Service hc	<5km	Didn't attend - primary	Secondary school	No	Yes	No	No	1-2 pregnancies	didn't-junior school	didn't-junior school	Private clinic	Yes	No problem reported	Health complex & providers	Employed/Business	=>4 ANC	>300 tk
188	26	Khagrachhari s	16-24	Tripura	Sonatoni	No	No	Yes	No							Young (15-29 yrs)	became preg <19	Yes	10000-19000		Involve in income generation		No		daily lab/f	<5km	Didn't attend - primary	Didn't attend - Primary	No	No	No	No	3 & above pregnancies	didn't-junior school	didn't-junior school	Community_based centres	Yes	Severe (need medical attention)		Daily labor/Farmer	Did not access	
189	26	Khagrachhari s	16-24	Chakma	Buddhist	No	No	No	No							Young (15-29 yrs)	became preg <19	Yes	10000-19000		Involve in income generation		No		daily lab/f	<5km	Didn't attend - primary	Didn't attend - Primary	No	No	No	No	1-2 pregnancies	didn't-junior school	didn't-junior school	Community_based centres	Yes	No problem reported		Daily labor/Farmer	Did not access	
190	27	Khagrachhari s	16-24	Chakma	Buddhist	No	No	No	Yes	Yes	Yes	Yes	No	Bangla	No	Young (15-29 yrs)	became pregnant 20+ yrs	Yes	20000 and above	Foetus sex	Complete housewife	2nd trimesters	No	Private clinic/doctor	Business	6-10km	Didn't attend - primary	Secondary school	No	Yes	No	No	1-2 pregnancies	didn't-junior school	secondary & above	Private clinic	Yes	No problem reported	Health complex & providers	Employed/Business	<4 ANC	>300 tk
191	27	Khagrachhari s	=>30	Chakma	Buddhist	No	No	Yes	Yes	Yes	Yes	Yes	Yes	Your own language		Adult (30 & above v	became pregnant 20+ yrs	Yes	20000 and above	Perceived appro. time	Involve in income generation	2nd trimesters	Yes	Private clinic/doctor	daily lab/f	6-10km	Higher secondary & above	Higher secondary & above	Yes	Yes	No	No	1-2 pregnancies	Secondary & above	secondary & above	Private clinic	Yes	Mild	Health complex & providers	Daily labor/Farmer	=>4 ANC	>300 tk
192	27	Khagrachhari s	=>30	Chakma	Buddhist	No	No	Yes	Yes	Yes	Yes	Yes	No	Bangla	No	Adult (30 & above v	became pregnant 20+ yrs	Yes	20000 and above	Know foetus condition	Complete housewife	1st trimesters	Yes	Private clinic/doctor	Business	6-10km	Secondary school	Secondary school	Yes	Yes	Yes	No	1-2 pregnancies	Secondary & above	secondary & above	Private clinic	Yes	Mild	Relatives & other	Employed/Business	<4 ANC	>300 tk
193	27	Khagrachhari s	=>30	Chakma	Buddhist	No	Yes	Yes	Yes	Yes	Yes	Yes	No	Bangla	Yes	Adult (30 & above v	became pregnant 20+ yrs	Yes	20000 and above	Illness/complications	Complete housewife	1st trimesters	Yes	Govt Health Comple	daily lab/f	6-10km	Higher secondary & above	Higher secondary & above	No	Yes	Yes	No	1-2 pregnancies	Secondary & above	secondary & above	Private clinic	Yes	Mild	Relatives & other	Daily labor/Farmer	=>4 ANC	>300 tk
194	27	Khagrachhari s	25-29	Chakma	Buddhist	Yes	No	No	Yes	Yes	Yes	No	No	Bangla	No	Young (15-29 yrs)	became pregnant 20+ yrs	Yes	10000-19000	Illness/complications	Complete housewife	1st trimesters	No	Community health w	daily lab/f	6-10km	Secondary school	Secondary school	No	Yes	No	No	1-2 pregnancies	Secondary & above	secondary & above	Private clinic	Yes	No problem reported	Health complex & providers	Daily labor/Farmer	<4 ANC	Did not pay
195	27	Khagrachhari s	25-29	Marma	Buddhist	No	No	Yes	Yes	Yes	Yes	Yes	No	Bangla	Yes	Young (15-29 yrs)	became pregnant 20+ yrs	Yes	20000 and above	Illness/complications	Complete housewife	2nd trimesters	Yes	Private clinic/doctor	daily lab/f	6-10km	Higher secondary & above	Higher secondary & above	No	Yes	No	No	1-2 pregnancies	Secondary & above	secondary & above	Private clinic	Yes	Severe (need medical attention)	Health complex & providers	Daily labor/Farmer	=>4 ANC	>300 tk
196	27	Khagrachhari s	=>30	Chakma	Buddhist	Yes	Yes	No	Yes	Yes	Yes	No	Yes	Your own language		Adult (30 & above v	became pregnant 20+ yrs	Yes	20000 and above	Confirm pregnancy	Complete housewife	1st trimesters	Yes	Private clinic/doctor	daily lab/f	6-10km	Higher secondary & above	Higher secondary & above	No	Yes	No	No	1-2 pregnancies	Secondary & above	secondary & above	Private clinic	Yes	No problem reported	Health complex & providers	Daily labor/Farmer	=>4 ANC	Did not pay
197	27	Khagrachhari s	16-24	Chakma	Buddhist	No	No	Yes	Yes	Yes	Yes	No	No	Bangla	No	Young (15-29 yrs)	became preg <19	Yes	20000 and above	Illness/complications	Complete housewife	1st trimesters	Yes	Private clinic/doctor	Service hc	6-10km	Secondary school	Higher secondary & above	No	Yes	No	No	1-2 pregnancies	Secondary & above	secondary & above	Private clinic	Yes	Severe (need medical attention)	Health complex & providers	Employed/Business	=>4 ANC	Did not pay
198	27	Khagrachhari s	16-24	Marma	Buddhist	No	No	No	Yes	Yes	Yes	Yes	No	Bangla	Yes	Young (15-29 yrs)	became pregnant 20+ yrs	Yes	20000 and above	Foetus sex	Complete housewife	2nd trimesters	Yes	Private clinic/doctor	Business	6-10km	Higher secondary & above	Higher secondary & above	No	Yes	No	No	1-2 pregnancies	Secondary & above	secondary & above	Private clinic	Yes	No problem reported	Health complex & providers	Employed/Business	<4 ANC	>300 tk
199	27	Khagrachhari s	=>30	Chakma	Buddhist	Yes	No	Yes	Yes	Yes	Yes	Yes	No	Bangla	Yes	Adult (30 & above v	became pregnant 20+ yrs	Yes	20000 and above	Foetus sex	Complete housewife	1st trimesters	Yes	Private clinic/doctor	Business	6-10km	Secondary school	Secondary school	No	Yes	No	No	3 & above pregnancies	Secondary & above	secondary & above	Private clinic	Yes	Severe (need medical attention)	Relatives & other	Employed/Business	<4 ANC	>300 tk
200	27	Khagrachhari s	=>30	Chakma	Buddhist	No	Yes	Yes	Yes	Yes	Yes	No	No	Bangla		Adult (30 & above v	became pregnant 20+ yrs	Yes	20000 and above	Confirm pregnancy	Complete housewife	1st trimesters	No	Private clinic/doctor	daily lab/f	6-10km	Higher secondary & above	Higher secondary & above	No	Yes	No	No	1-2 pregnancies	Secondary & above	secondary & above	Private clinic	Yes	Mild	Health complex & providers	Daily labor/Farmer	=>4 ANC	Did not pay
201	27	Khagrachhari s	=>30	Chakma	Buddhist	Yes	Yes	No	Yes	Yes	Yes	No	Yes	Bangla	Yes	Adult (30 & above v	became pregnant 20+ yrs	Yes	20000 and above	Perceived appro. time	Involve in income generation	1st trimesters	Yes	Community health w	Business	6-10km	Secondary school	Higher secondary & above	No	No	No	Yes	1-2 pregnancies	Secondary & above	secondary & above	Private clinic	Yes	No problem reported	Relatives & other	Employed/Business	=>4 ANC	Did not pay
202	27	Khagrachhari s	=>30	Chakma	Buddhist	No	No	Yes	Yes	Yes	Yes	Yes	No	Bangla	No	Adult (30 & above v	became pregnant 20+ yrs	Yes	4000-9000	Illness/complications	Complete housewife	1st trimesters	No	Private clinic/doctor	daily lab/f	6-10km	Didn't attend - primary	Secondary school	No	Yes	No	No	1-2 pregnancies	didn't-junior school	didn't-junior school	Private clinic	Yes	Mild	Health complex & providers	Daily labor/Farmer	=>4 ANC	>300 tk
203	27	Khagrachhari s	16-24	Chakma	Buddhist	No	Yes	Yes	Yes	Yes	Yes	Yes	No	Bangla	No	Young (15-29 yrs)	became preg <19	Yes	20000 and above	Confirm pregnancy	Complete housewife	1st trimesters	Yes	Private clinic/doctor	Business	6-10km	Secondary school	Higher secondary & above	No	Yes	Yes	No	1-2 pregnancies	Secondary & above	secondary & above	Private clinic	Yes	Mild	Relatives & other	Employed/Business	=>4 ANC	>300 tk
204	27	Khagrachhari s	25-29	Chakma	Buddhist	No	Yes	No	Yes	Yes	Yes	Yes	No	Bangla	Yes	Young (15-29 yrs)	became pregnant 20+ yrs	Yes	20000 and above	Illness/complications	Complete housewife	1st trimesters	No	Private clinic/doctor	Business	6-10km	Higher secondary & above	Higher secondary & above	No	Yes	Yes	No	1-2 pregnancies	Secondary & above	secondary & above	Private clinic	Yes	No problem reported	Relatives & other	Employed/Business	=>4 ANC	>300 tk
205	27	Khagrachhari s	25-29	Chakma	Buddhist	No	No	No	Yes	Yes	Yes	Yes	No	Bangla	No	Young (15-29 yrs)	became pregnant 20+ yrs	Yes	20000 and above	Perceived appro. time	Complete housewife	2nd trimesters	Yes	Private clinic/doctor	Business	6-10km	Secondary school	Higher secondary & above	No	No	Yes	No	1-2 pregnancies	Secondary & above	secondary & above	Private clinic	Yes	No problem reported	Relatives & other	Employed/Business	=>4 ANC	>300 tk
206	27	Khagrachhari s	=>30	Chakma	Buddhist	No	Yes	Yes	Yes	Yes	Yes	Yes	No	Bangla	Yes	Adult (30 & above v	became preg <19	Yes	20000 and above	Confirm pregnancy	Complete housewife	1st trimesters	Yes	Private clinic/doctor	Service hc	6-10km	Higher secondary & above	Higher secondary & above	Yes	Yes	No	No	1-2 pregnancies	Secondary & above	secondary & above	Private clinic	Yes	Severe (need medical attention)	Health complex & providers	Employed/Business	=>4 ANC	>300 tk
207	27	Khagrachhari s	=>30	Chakma	Buddhist	No	No	No	Yes	Yes	Yes	No	No	Bangla	Yes	Adult (30 & above v	became preg <19	Yes	20000 and above	Confirm pregnancy	Complete housewife	1st trimesters	Yes	Govt Health Comple	Service hc	6-10km	Higher secondary & above	Higher secondary & above	No	Yes	Yes	No	1-2 pregnancies	Secondary & above	secondary & above	Private clinic	Yes	No problem reported	Relatives & other	Employed/Business	=>4 ANC	Did not pay
208	27	Khagrachhari s	=>30	Chakma	Buddhist	No	No	No	Yes	Yes	Yes	Yes	No	Bangla	Yes	Adult (30 & above v	became pregnant 20+ yrs	Yes	20000 and above	Confirm pregnancy	Complete housewife	1st trimesters	Yes	Private clinic/doctor	daily lab/f	6-10km	Secondary school	Higher secondary & above	No	Yes	No	No	1-2 pregnancies	Secondary & above	secondary & above	Private clinic	Yes	No problem reported	Health complex & providers	Daily labor/Farmer	<4 ANC	>300 tk
209	27	Khagrachhari s	25-29	Chakma	Buddhist	No	Yes	No	Yes	Yes	Yes	Yes	Yes	Bangla	Yes	Young (15-29 yrs)	became pregnant 20+ yrs	Yes	20000 and above	Illness/complications	Involve in income generation	1st trimesters	Yes	Private clinic/doctor	Service hc	6-10km	Higher secondary & above	Higher secondary & above	Yes	Yes	No	No	1-2 pregnancies	Secondary & above	secondary & above	Private clinic	Yes	No problem reported	Health complex & providers	Employed/Business	=>4 ANC	<=300 tk
210	27	Khagrachhari s	25-29	Chakma	Buddhist	No	Yes	Yes	Yes	Yes	Yes	Yes	No	Bangla	Yes	Young (15-29 yrs)	became pregnant 20+ yrs	Yes	20000 and above	Illness/complications	Involve in income generation	1st trimesters	Yes	Private clinic/doctor	Service hc	6-10km	Higher secondary & above	Higher secondary & above	No	Yes	No	No	1-2 pregnancies	Secondary & above	secondary & above	Private clinic	Yes	Moderate (debiliated daily life)	Health complex & providers	Employed/Business	=>4 ANC	>300 tk
211	27	Khagrachhari s	25-29	Chakma	Buddhist	No	No	No	Yes	Yes	Yes	Yes	No	Bangla	Yes	Adult (30 & above v	became pregnant 20+ yrs	Yes	20000 and above	Know foetus condition	Complete housewife	1st trimesters	Yes	Private clinic/doctor	Service hc	6-10km	Higher secondary & above	Higher secondary & above	No	Yes	No	No	1-2 pregnancies	Secondary & above	secondary & above	Private clinic	Yes	No problem reported	Health complex & providers	Employed/Business	=>4 ANC	>300 tk
212	27	Khagrachhari s	=>30	Chakma	Buddhist	No	No	Yes	Yes	Yes	Yes	No	No	Bangla	No	Adult (30 & above v	became pregnant 20+ yrs	Yes	20000 and above	Illness/complications	Complete housewife	1st trimesters	Yes	Community health w	Business	6-10km	Secondary school	Higher secondary & above	Yes	Yes	No	No	1-2 pregnancies	Secondary & above	secondary & above	Private clinic	Yes	Severe (need medical attention)	Health complex & providers	Employed/Business	=>4 ANC	Did not pay
213	27	Khagrachhari s	16-24	Chakma	Buddhist	No	Yes	Yes	Yes	Yes	Yes	Yes	No	Bangla	Yes	Young (15-29 yrs)	became preg <19	Yes	20000 and above	Confirm pregnancy	Complete housewife	1st trimesters	Yes	Private clinic/doctor	Service hc	6-10km	Higher secondary & above	Higher secondary & above	No	Yes	No	No	1-2 pregnancies	Secondary & above	secondary & above	Private clinic	Yes	Mild	Health complex & providers	Employed/Business	<4 ANC	>300 tk
214	27	Khagrachhari s	25-29	Chakma	Buddhist	No	No	No	Yes	Yes	Yes	Yes	No	Bangla	No	Young (15-29 yrs)	became pregnant 20+ yrs	Yes	20000 and above	Illness/complications	Complete housewife	1st trimesters	Yes	Private clinic/doctor	daily lab/f	6-10km	Secondary school	Secondary school	No	Yes	No	No	1-2 pregnancies	Secondary & above	secondary & above	Private clinic	Yes	No problem reported	Health complex & providers	Daily labor/Farmer	<4 ANC	>300 tk
215	27	Khagrachhari s	16-24	Chakma	Buddhist	No	No	Yes	No							Young (15-29 yrs)	became pregnant 20+ yrs	Yes	10000-19000		Complete housewife		Yes		Business	6-10km	Secondary school	Secondary school	No	No	No	No	1-2 pregnancies	didn't-junior school	didn't-junior school	Private clinic	Yes	Mild		Employed/Business	Did not access	
216	27	Khagrachhari s	16-24	Chakma	Buddhist	No	No	No	Yes	Don't kno	Yes	No	No	Bangla	No	Young (15-29 yrs)	became preg <19	Yes	4000-9000	Confirm pregnancy	Complete housewife	1st trimesters	No	UHC or other priman	daily lab/f	6-10km	Didn't attend - primary	Didn't attend - Primary	No	No	Yes	No	1-2 pregnancies	didn't-junior school	didn't-junior school	Private clinic	Yes	No problem reported	Relatives & other	Daily labor/Farmer	<4 ANC	Did not pay
217	27	Khagrachhari s	16-24	Chakma	Buddhist	No	No	Yes	Yes	Yes	Yes	Yes	No	Bangla	No	Young (15-29 yrs)	became preg <19	Yes	20000 and above	Confirm pregnancy	Complete housewife	1st trimesters	Yes	Private clinic/doctor	daily lab/f	6-10km	Secondary school	Higher secondary & above	Yes	Yes	No	No	1-2 pregnancies	Secondary & above	secondary & above	Private clinic	Yes	Severe (need medical attention)	Health complex & providers	Daily labor/Farmer	=>4 ANC	>300 tk
218	27	Khagrachhari s	25-29	Chakma	Buddhist	No	No	Yes	Yes	Yes	Yes	No	Yes	Your own language		Young (15-29 yrs)	became preg <19	Yes	20000 and above	Confirm pregnancy	Complete housewife	1st trimesters	Yes	UHC or other priman	daily lab/f	6-10km	Higher secondary & above	Secondary school	No	No	Yes	No	1-2 pregnancies	Secondary & above	secondary & above	Private clinic	Yes	Mild	Relatives & other	Daily labor/Farmer	=>4 ANC	Did not pay
219	28	Khagrachhari s	16-24	Chakma	Buddhist	No	No	Yes	No							Young (15-29 yrs)	became preg <19	Yes	10000-19000		Complete housewife		No		daily lab/f	6-10km	Secondary school	Secondary school	No	No	No	No	1-2 pregnancies	Secondary & above	didn't-junior school	Secondary hospitals	Yes	Mild		Daily labor/Farmer	Did not access	
220	28	Khagrachhari s	16-24	Chakma	Buddhist	No	Yes	Yes	Yes	Yes	Yes	Yes	No	Bangla	No	Young (15-29 yrs)	became preg <19	Yes	10000-19000	Confirm pregnancy	Involve in income generation	1st trimesters	No	Home visit by HW/w	daily lab/f	6-10km	Didn't attend - primary	Didn't attend - Primary	No	Yes	No	No	1-2 pregnancies	didn't-junior school	didn't-junior school	Private clinic	Yes	Mild	Health complex & providers	Daily labor/Farmer	<4 ANC	<=300 tk
221	28	Khagrachhari s	=>30	Chakma	Buddhist	No	No	No	Yes	Yes	Yes	No	No	Bangla	No	Adult (30 & above v	became pregnant 20+ yrs	Yes	10000-19000	Confirm pregnancy	Complete housewife	1st trimesters	No	UHC or other priman	Business	6-10km	Didn't attend - primary	Secondary school	Yes	Yes	Yes	Yes	1-2 pregnancies	didn't-junior school	secondary & above	Private clinic	Yes	No problem reported	Relatives & other	Employed/Business	=>4 ANC	Did not pay
222	28	Khagrachhari s	16-24	Chakma	Buddhist	No	No	No	Yes	Don't kno	Yes	Yes	No	Bangla	No	Young (15-29 yrs)	became pregnant 20+ yrs	Yes	10000-19000	Foetus sex	Involve in income generation	2nd trimesters	No	Private clinic/doctor	daily lab/f	6-10km	Didn't attend - primary	Secondary school	No	Yes	Yes	No	1-2 pregnancies	didn't-junior school	didn't-junior school	Secondary hospitals	Yes	No problem reported	Relatives & other	Daily labor/Farmer	=>4 ANC	Did not pay
223	28	Khagrachhari s	16-24	Chakma	Buddhist	No	No	Yes	Yes	Yes	Yes	Yes	No	Bangla	Yes	Young (15-29 yrs)	became preg <19	Yes	20000 and above	Foetus sex	Complete housewife	3rd trimesters	No	Private clinic/doctor	Business	6-10km	Secondary school	Secondary school	No	No	Yes	No	1-2 pregnancies	Secondary & above	secondary & above	Secondary hospitals	Yes	Mild	Relatives & other	Employed/Business	<4 ANC	>300 tk
224	28	Khagrachhari s	16-24	Chakma	Buddhist	No	No	No	Yes	Yes	Yes	No	No	Bangla	No	Young (15-29 yrs)	became preg <19	Yes	4000-9000	Foetus sex	Involve in income generation	3rd trimesters	No	Private clinic/doctor	daily lab/f	6-10km	Didn't attend - primary	Didn't attend - Primary	No	Yes	Yes	No	1-2 pregnancies	didn't-junior school	didn't-junior school	Secondary hospitals	Yes	No problem reported	Relatives & other	Daily labor/Farmer	<4 ANC	Did not pay
225	28	Khagrachhari s	25-29	Chakma	Buddhist	No	No	No	Yes	Yes	Yes	Yes	No	Bangla	No	Young (15-29 yrs)	became preg <19	Yes	10000-19000	Confirm pregnancy	Complete housewife	1st trimesters	No	Private clinic/doctor	Service hc	6-10km	Didn't attend - primary	Didn't attend - Primary	No	Yes	Yes	No	3 & above pregnancies	didn't-junior school	didn't-junior school	Secondary hospitals	Yes	No problem reported	Relatives & other	Employed/Business	<4 ANC	>300 tk
226	28	Khagrachhari s	=>30	Chakma	Buddhist	No	No	No	Yes	Yes	Yes	No	No	Bangla	No	Adult (30 & above v	became pregnant 20+ yrs	Yes	20000 and above	Know foetus condition	Complete housewife	1st trimesters	No	Community health w	Business	6-10km	Secondary school	Secondary school	No	Yes	No	No	1-2 pregnancies	Secondary & above	secondary & above	Secondary hospitals	Yes	No problem reported	Health complex & providers	Employed/Business	<4 ANC	Did not pay
227	28	Khagrachhari s	25-29	Chakma	Buddhist	No	Yes	Yes	Yes	Yes	Yes	No	No	Bangla	No	Young (15-29 yrs)	became pregnant 20+ yrs	Yes	10000-19000	other	Complete housewife	1st trimesters	No	Govt Health Comple	Business	6-10km	Higher secondary & above	Higher secondary & above	Yes	Yes	Yes	No	1-2 pregnancies	Secondary & above	secondary & above	Secondary hospitals	Yes	Mild	Relatives & other	Employed/Business	=>4 ANC	Did not pay
228	28	Khagrachhari s	16-24	Chakma	Buddhist	No	Yes	Yes	Yes	Yes	Yes	No	No	Bangla	No	Young (15-29 yrs)	became preg <19	Yes	10000-19000	Illness/complications	Complete housewife	1st trimesters	Yes	UHC or other priman	Service hc	6-10km	Secondary school	Higher secondary & above	No	Yes	Yes	No	1-2 pregnancies	Secondary & above	secondary & above	Secondary hospitals	Yes	Mild	Health complex & providers	Employed/Business	=>4 ANC	Did not pay
229	28	Khagrachhari s	=>30	Chakma	Buddhist	Yes	Yes	Yes	Yes	Yes	Yes	No	Yes	Your own language		Adult (30 & above v	became pregnant 20+ yrs	Yes	20000 and above	Perceived appro. time	Involve in income generation	2nd trimesters	Yes	Community health w	Service hc	6-10km	Secondary school	Secondary school	No	No	Yes	No	3 & above pregnancies	Secondary & above	secondary & above	Private clinic	Yes	Mild	Relatives & other	Employed/Business	<4 ANC	Did not pay
230	28	Khagrachhari s	16-24	Chakma	Buddhist	No	No	Yes	Yes	Yes	Yes	Yes	No	Bangla	Yes	Young (15-29 yrs)	became preg <19	Yes	20000 and above	Confirm pregnancy	Complete housewife	1st trimesters	Yes	Home visit by HW/w	Business	6-10km	Secondary school	Secondary school	No	Yes	No	No	1-2 pregnancies	Secondary & above	secondary & above	Private clinic	Yes	Severe (need medical attention)	Health complex & providers	Employed/Business	=>4 ANC	<=300 tk
231	28	Khagrachhari s	25-29	Chakma	Buddhist	No	Yes	Yes	Yes	Yes	Yes	Yes	No	Bangla	No	Young (15-29 yrs)	became preg <19	Yes	20000 and above	Confirm pregnancy	Involve in income generation	1st trimesters	Yes	UHC or other priman	Business	6-10km	Secondary school	Higher secondary & above	No	Yes	Yes	No	1-2 pregnancies	Secondary & above	secondary & above	Private clinic	Yes	Mild	Relatives & other	Employed/Business	=>4 ANC	>300 tk
232	28	Khagrachhari s	=>30	Chakma	Buddhist	No	No	Yes	Yes	Yes	Yes	Yes	No	Bangla	Yes	Adult (30 & above v	became pregnant 20+ yrs	Yes	20000 and above	Confirm pregnancy	Involve in income generation	1st trimesters	No	Home visit by HW/w	Service hc	6-10km	Secondary school	Secondary school	No	Yes	Yes	No	3 & above pregnancies	Secondary & above	secondary & above	Secondary hospitals	Yes	Mild	Relatives & other	Employed/Business	=>4 ANC	<=300 tk
233	28	Khagrachhari s	25-29	Chakma	Buddhist	No	No	Yes	Yes	Yes	Yes	No	Yes	Bangla	Yes	Young (15-29 yrs)	became pregnant 20+ yrs	Yes	20000 and above	Confirm pregnancy	Complete housewife	1st trimesters	Yes	Private clinic/doctor	daily lab/f	6-10km	Secondary school	Secondary school	Yes	Yes	No	No	1-2 pregnancies	Secondary & above	didn't-junior school	Private clinic	Yes	Mild	Health complex & providers	Daily labor/Farmer	=>4 ANC	Did not pay
234	28	Khagrachhari s	16-24	Chakma	Buddhist	Yes	No	No	Yes	Yes	Yes	No	Yes	Bangla	Yes	Young (15-29 yrs)	became preg <19	Yes	4000-9000	Confirm pregnancy	Involve in income generation	1st trimesters	No	Private clinic/doctor	daily lab/f	6-10km	Secondary school	Didn't attend - Primary	No	Yes	Yes	No	1-2 pregnancies	Secondary & above	didn't-junior school	Private clinic	Yes	No problem reported	Relatives & other	Daily labor/Farmer	=>4 ANC	Did not pay
235	28	Khagrachhari s	16-24	Chakma	Buddhist	No	No	No	Yes	Yes	Yes	Yes	No	Bangla	No	Young (15-29 yrs)	became preg <19	Yes	4000-9000	Foetus sex	Complete housewife	3rd trimesters	No	Private clinic/doctor	Service hc	6-10km	Didn't attend - primary	Didn't attend - Primary	No	No	Yes	No	1-2 pregnancies	didn't-junior school	didn't-junior school	Secondary hospitals	Yes	No problem reported	Relatives & other	Employed/Business	=>4 ANC	>300 tk
236	28	Khagrachhari s	25-29	Chakma	Buddhist	No	Yes	Yes	Yes	Yes	Yes	Yes	Yes	Bangla	No	Young (15-29 yrs)	became pregnant 20+ yrs	Yes	20000 and above	Confirm pregnancy	Involve in income generation	2nd trimesters	Yes	Home visit by HW/w	daily lab/f	6-10km	Secondary school	Secondary school	No	Yes	No	No	1-2 pregnancies	Secondary & above	secondary & above	Private clinic	Yes	Mild	Health complex & providers	Daily labor/Farmer	<4 ANC	<=300 tk
237	28	Khagrachhari s	16-24	Chakma	Buddhist	Yes	No	No	No							Young (15-29 yrs)	became preg <19	Yes	20000 and above		Involve in income generation		No		Service hc	6-10km	Didn't attend - primary	Secondary school	No	No	No	No	1-2 pregnancies	didn't-junior school	didn't-junior school	Secondary hospitals	Yes	No problem reported		Employed/Business	Did not access	
238	28	Khagrachhari s	25-29	Chakma	Buddhist	No	No	Yes	Yes	Yes	Yes	No	No	Bangla	Yes	Young (15-29 yrs)	became preg <19	Yes	20000 and above	Confirm pregnancy	Complete housewife	1st trimesters	No	Community health w	daily lab/f	6-10km	Secondary school	Didn't attend - Primary	No	Yes	Yes	No	3 & above pregnancies	Secondary & above	didn't-junior school	Secondary hospitals	Yes	Severe (need medical attention)	Relatives & other	Daily labor/Farmer	<4 ANC	Did not pay
239	28	Khagrachhari s	16-24	Chakma	Buddhist	No	No	No	Yes	Don't kno	Yes	Yes	No	Bangla	No	Young (15-29 yrs)	became preg <19	Yes	10000-19000	Confirm pregnancy	Complete housewife	1st trimesters	No	Home visit by HW/w	daily lab/f	6-10km	Secondary school	Didn't attend - Primary	Yes	Yes	No	No	1-2 pregnancies	didn't-junior school	didn't-junior school	Secondary hospitals	Yes	No problem reported	Relatives & other	Daily labor/Farmer	=>4 ANC	<=300 tk
240	28	Khagrachhari s	16-24	Chakma	Buddhist	No	No	Yes	Yes	Don't kno	Yes	Yes	No	Bangla	No	Young (15-29 yrs)	became preg <19	No	10000-19000	HW came home	Involve in income generation	2nd trimesters	No	Home visit by HW/w	daily lab/f	6-10km	Didn't attend - primary	Didn't attend - Primary	No	Yes	No	No	1-2 pregnancies	didn't-junior school	didn't-junior school	Do not know	No	Severe (need medical attention)	Health complex & providers	Daily labor/Farmer	=>4 ANC	<=300 tk
241	28	Khagrachhari s	16-24	Chakma	Buddhist	No	No	Yes	No							Young (15-29 yrs)	became preg <19	No	10000-19000		Involve in income generation		No		Business	6-10km	Didn't attend - primary	Didn't attend - Primary	No	No	No	No	1-2 pregnancies	didn't-junior school	didn't-junior school	Do not know	No	Severe (need medical attention)		Employed/Business	Did not access	
242	28	Khagrachhari s	=>30	Chakma	Buddhist	No	Yes	Yes	Yes	Don't kno	Yes	Yes	Yes	Bangla	No	Adult (30 & above v	became preg <19	Yes	20000 and above	Confirm pregnancy	Involve in income generation	1st trimesters	No	Home visit by HW/w	Business	6-10km	Secondary school	Secondary school	No	Yes	No	No	3 & above pregnancies	didn't-junior school	didn't-junior school	Private clinic	Yes	Severe (need medical attention)	Health complex & providers	Employed/Business	=>4 ANC	<=300 tk
243	28	Khagrachhari s	=>30	Chakma	Buddhist	Yes	Yes	Yes	Yes	Yes	Yes	No	No	Bangla	No	Adult (30 & above v	became pregnant 20+ yrs	Yes	20000 and above	Confirm pregnancy	Involve in income generation	1st trimesters	No	UHC or other priman	Service hc	6-10km	Secondary school	Higher secondary & above	No	Yes	Yes	No	3 & above pregnancies	Secondary & above	secondary & above	Secondary hospitals	Yes	Mild	Relatives & other	Employed/Business	<4 ANC	Did not pay
244	28	Khagrachhari s	16-24	Chakma	Buddhist	No	No	No	Yes	Yes	Yes	No	No	Bangla	No	Young (15-29 yrs)	became preg <19	Yes	10000-19000	Know foetus condition	Complete housewife	2nd trimesters	No	Home visit by HW/w	daily lab/f	6-10km	Secondary school	Secondary school	No	Yes	No	No	1-2 pregnancies	didn't-junior school	didn't-junior school	Community_based centres	Yes	No problem reported	Health complex & providers	Daily labor/Farmer	<4 ANC	Did not pay
245	28	Khagrachhari s	16-24	Chakma	Buddhist	No	No	No	No							Young (15-29 yrs)	became preg <19	No	20000 and above		Complete housewife		No		daily lab/f	6-10km	Didn't attend - primary	Didn't attend - Primary	No	No	No	No	1-2 pregnancies	didn't-junior school	didn't-junior school	Do not know	No	No problem reported		Daily labor/Farmer	Did not access	
246	28	Khagrachhari s	25-29	Chakma	Buddhist	No	No	Yes	No							Young (15-29 yrs)	became preg <19	Yes	10000-19000		Involve in income generation		No		daily lab/f	6-10km	Secondary school	Secondary school	No	No	No	No	3 & above pregnancies	Secondary & above	didn't-junior school	Private clinic	Yes	Severe (need medical attention)		Daily labor/Farmer	Did not access	
247	28	Khagrachhari s	=>30	Chakma	Buddhist	No	No	Yes	Yes	Yes	Yes	No	No	Bangla	Yes	Adult (30 & above v	became preg <19	Yes	20000 and above	Perceived appro. time	Involve in income generation	2nd trimesters	No	Community health w	Service hc	6-10km	Secondary school	Secondary school	No	No	Yes	No	3 & above pregnancies	didn't-junior school	secondary & above	Secondary hospitals	Yes	Mild	Relatives & other	Employed/Business	<4 ANC	Did not pay
248	29	Matiranga	16-24	Chakma	Buddhist	No	No	Yes	Yes	Don't kno	Yes	No	No	Bangla	No	Young (15-29 yrs)	became preg <19	Yes	4000-9000	Know foetus condition	Involve in income generation	1st trimesters	No	Private clinic/doctor	daily lab/f	6-10km	Didn't attend - primary	Didn't attend - Primary	No	Yes	No	No	1-2 pregnancies	didn't-junior school	didn't-junior school	Secondary hospitals	Yes	Severe (need medical attention)	Health complex & providers	Daily labor/Farmer	<4 ANC	Did not pay
249	29	Matiranga	16-24	Chakma	Buddhist	No	No	Yes	Yes	Don't kno	No					Young (15-29 yrs)	became preg <19	No	4000-9000		Involve in income generation		No		daily lab/f	6-10km	Didn't attend - primary	Secondary school	No	No	Yes	No	1-2 pregnancies	didn't-junior school	didn't-junior school	Do not know	No	Severe (need medical attention)	Relatives & other	Daily labor/Farmer	Did not access	
250	29	Matiranga	16-24	Chakma	Buddhist	No	No	No	No							Young (15-29 yrs)	became preg <19	No	10000-19000		Involve in income generation		No		daily lab/f	6-10km	Didn't attend - primary	Didn't attend - Primary	No	No	No	No	1-2 pregnancies	didn't-junior school	didn't-junior school	Do not know	No	No problem reported		Daily labor/Farmer	Did not access	
251	29	Matiranga	16-24	Chakma	Buddhist	No	No	No	No							Young (15-29 yrs)	became preg <19	No	4000-9000		Complete housewife		No		daily lab/f	6-10km	Didn't attend - primary	Didn't attend - Primary	No	No	No	No	1-2 pregnancies	didn't-junior school	didn't-junior school	Do not know	No	No problem reported		Daily labor/Farmer	Did not access	
252	29	Matiranga	=>30	Chakma	Buddhist	No	No	Yes	Yes	Yes	Yes	Yes	No	Bangla	No	Adult (30 & above v	became preg <19	Yes	20000 and above	Illness/complications	Involve in income generation	2nd trimesters	No	Private clinic/doctor	Service hc	6-10km	Didn't attend - primary	Secondary school	No	Yes	No	No	3 & above pregnancies	didn't-junior school	didn't-junior school	Community_based centres	Yes	Severe (need medical attention)	Health complex & providers	Employed/Business	=>4 ANC	<=300 tk
253	29	Matiranga	16-24	Chakma	Buddhist	Yes	No	Yes	Yes	Yes	Yes	Yes	No	Bangla	No	Young (15-29 yrs)	became pregnant 20+ yrs	Yes	4000-9000	Foetus sex	Involve in income generation	2nd trimesters	No	Private clinic/doctor	daily lab/f	6-10km	Didn't attend - primary	Secondary school	No	Yes	No	No	1-2 pregnancies	didn't-junior school	didn't-junior school	Community_based centres	Yes	Severe (need medical attention)	Health complex & providers	Daily labor/Farmer	<4 ANC	>300 tk
254	30	Matiranga	16-24	Marma	Buddhist	No	Yes	Yes	Yes	Yes	Yes	Yes	No	Bangla	Yes	Young (15-29 yrs)	became pregnant 20+ yrs	No	20000 and above	Foetus sex	Complete housewife	2nd trimesters	No	Private clinic/doctor	Service hc	6-10km	Higher secondary & above	Secondary school	No	No	Yes	No	1-2 pregnancies	Secondary & above	didn't-junior school	Do not know	No	Mild	Relatives & other	Employed/Business	<4 ANC	>300 tk
255	30	Matiranga	25-29	Marma	Buddhist	No	Yes	Yes	Yes	Yes	Yes	Yes	No	Bangla	Yes	Young (15-29 yrs)	became preg <19	Yes	20000 and above	other	Involve in income generation	1st trimesters	Yes	Private clinic/doctor	Business	6-10km	Higher secondary & above	Higher secondary & above	Yes	Yes	No	No	1-2 pregnancies	Secondary & above	secondary & above	Private clinic	Yes	Mild	Health complex & providers	Employed/Business	=>4 ANC	>300 tk
256	30	Matiranga	16-24	Marma	Buddhist	No	No	No	No							Young (15-29 yrs)	became preg <19	No	4000-9000		Complete housewife		No		daily lab/f	6-10km	Secondary school	Didn't attend - Primary	No	No	No	No	1-2 pregnancies	didn't-junior school	didn't-junior school	Do not know	No	No problem reported		Daily labor/Farmer	Did not access	
257	30	Matiranga	16-24	Marma	Buddhist	No	No	No	Yes	Don't kno	No					Young (15-29 yrs)	became preg <19	Yes	10000-19000		Complete housewife		Yes		daily lab/f	6-10km	Secondary school	Secondary school	Yes	Yes	No	No	1-2 pregnancies	didn't-junior school	secondary & above	Secondary hospitals	Yes	No problem reported	Health complex & providers	Daily labor/Farmer	Did not access	
258	30	Matiranga	16-24	Marma	Buddhist	No	No	No	Yes	Don't kno	No					Young (15-29 yrs)	became preg <19	No	4000-9000		Complete housewife		No		daily lab/f	6-10km	Didn't attend - primary	Didn't attend - Primary	No	Yes	No	No	1-2 pregnancies	didn't-junior school	didn't-junior school	Do not know	No	No problem reported	Health complex & providers	Daily labor/Farmer	Did not access	
259	30	Matiranga	16-24	Marma	Buddhist	No	No	No	Yes	Don't kno	No					Young (15-29 yrs)	became preg <19	Yes	4000-9000		Involve in income generation		No		daily lab/f	6-10km	Didn't attend - primary	Didn't attend - Primary	No	Yes	No	No	1-2 pregnancies	didn't-junior school	didn't-junior school	Secondary hospitals	Yes	No problem reported	Health complex & providers	Daily labor/Farmer	Did not access	
260	30	Matiranga	16-24	Marma	Buddhist	No	Yes	Yes	Yes	Don't kno	No					Young (15-29 yrs)	became preg <19	No	4000-9000		Complete housewife		No		daily lab/f	6-10km	Didn't attend - primary	Didn't attend - Primary	No	Yes	Yes	No	1-2 pregnancies	didn't-junior school	didn't-junior school	Do not know	No	Mild	Relatives & other	Daily labor/Farmer	Did not access	
261	30	Matiranga	25-29	Chakma	Buddhist	No	No	Yes	Yes	Yes	Yes	Yes	No	Bangla	Yes	Young (15-29 yrs)	became pregnant 20+ yrs	Yes	20000 and above	Confirm pregnancy	Complete housewife	1st trimesters	No	Community health w	Service hc	6-10km	Higher secondary & above	Higher secondary & above	No	No	Yes	No	1-2 pregnancies	Secondary & above	secondary & above	Community_based centres	Yes	Mild	Relatives & other	Employed/Business	<4 ANC	<=300 tk
262	31	Matiranga	25-29	Marma	Buddhist	No	Yes	Yes	Yes	Yes	Yes	No	No	Bangla	Yes	Young (15-29 yrs)	became preg <19	Yes	20000 and above	Perceived appro. time	Involve in income generation	2nd trimesters	No	Govt Health Comple	Business	<5km	Secondary school	Secondary school	No	Yes	No	No	3 & above pregnancies	didn't-junior school	didn't-junior school	Private clinic	Yes	Mild	Health complex & providers	Employed/Business	=>4 ANC	Did not pay
263	31	Matiranga	=>30	Marma	Buddhist	No	No	Yes	Yes	Yes	Yes	No	No	Bangla	Yes	Adult (30 & above v	became pregnant 20+ yrs	Yes	20000 and above	Confirm pregnancy	Complete housewife	2nd trimesters	Yes	UHC or other priman	Business	<5km	Secondary school	Secondary school	No	Yes	No	No	1-2 pregnancies	didn't-junior school	secondary & above	Secondary hospitals	Yes	Severe (need medical attention)	Relatives & other	Employed/Business	=>4 ANC	Did not pay
264	31	Matiranga	25-29	Marma	Buddhist	Yes	No	No	Yes	Don't kno	Yes	Yes	No	Bangla	No	Young (15-29 yrs)	became pregnant 20+ yrs	Yes	20000 and above	other	Involve in income generation	2nd trimesters	No	Private clinic/doctor	Business	<5km	Secondary school	Secondary school	No	No	Yes	No	1-2 pregnancies	didn't-junior school	didn't-junior school	Secondary hospitals	Yes	No problem reported	Relatives & other	Employed/Business	=>4 ANC	<=300 tk
265	31	Matiranga	16-24	Marma	Buddhist	No	Yes	Yes	Yes	Yes	Yes	No	No	Bangla	No	Young (15-29 yrs)	became preg <19	Yes	4000-9000	Know foetus condition	Complete housewife	3rd trimesters	No	Home visit by HW/w	daily lab/f	<5km	Secondary school	Didn't attend - Primary	No	Yes	No	No	1-2 pregnancies	Secondary & above	didn't-junior school	Secondary hospitals	Yes	Severe (need medical attention)	Health complex & providers	Daily labor/Farmer	<4 ANC	Did not pay
266	31	Matiranga	25-29	Marma	Buddhist	No	No	No	Yes	Yes	Yes	Yes	No	Bangla	Yes	Young (15-29 yrs)	became preg <19	Yes	10000-19000	Know foetus condition	Complete housewife	3rd trimesters	No	Home visit by HW/w	Business	<5km	Secondary school	Didn't attend - Primary	No	No	Yes	No	3 & above pregnancies	didn't-junior school	didn't-junior school	Secondary hospitals	Yes	No problem reported	Relatives & other	Employed/Business	<4 ANC	<=300 tk
267	31	Matiranga	=>30	Marma	Buddhist	No	Yes	Yes	Yes	Yes	Yes	No	No	Bangla	No	Adult (30 & above v	became pregnant 20+ yrs	Yes	20000 and above	Illness/complications	Complete housewife	2nd trimesters	No	Home visit by HW/w	Service hc	<5km	Didn't attend - primary	Secondary school	No	No	Yes	No	1-2 pregnancies	didn't-junior school	secondary & above	Secondary hospitals	Yes	Mild	Relatives & other	Employed/Business	<4 ANC	Did not pay
268	31	Matiranga	25-29	Marma	Buddhist	Yes	Yes	Yes	Yes	Yes	Yes	No	No	Bangla	No	Young (15-29 yrs)	became pregnant 20+ yrs	Yes	20000 and above	Illness/complications	Involve in income generation	2nd trimesters	No	Private clinic/doctor	Business	<5km	Secondary school	Higher secondary & above	No	No	Yes	No	1-2 pregnancies	Secondary & above	secondary & above	Private clinic	Yes	Mild	Relatives & other	Employed/Business	<4 ANC	Did not pay
269	32	Khagrachhari s	16-24	Marma	Buddhist	No	No	No	No							Young (15-29 yrs)	became preg <19	No	10000-19000		Complete housewife		No		daily lab/f	<5km	Didn't attend - primary	Secondary school	No	No	No	No	1-2 pregnancies	didn't-junior school	secondary & above	Do not know	No	No problem reported		Daily labor/Farmer	Did not access	
270	32	Khagrachhari s	25-29	Marma	Buddhist	No	No	Yes	Yes	Don't kno	No					Young (15-29 yrs)	became preg <19	Yes	10000-19000		Involve in income generation		No		daily lab/f	<5km	Didn't attend - primary	Didn't attend - Primary	No	Yes	No	No	3 & above pregnancies	didn't-junior school	didn't-junior school	Secondary hospitals	Yes	Mild	Health complex & providers	Daily labor/Farmer	Did not access	
271	32	Khagrachhari s	16-24	Marma	Buddhist	No	No	Yes	Yes	Don't kno	No					Young (15-29 yrs)	became preg <19	Yes	10000-19000		Complete housewife		No		daily lab/f	<5km	Secondary school	Didn't attend - Primary	No	Yes	No	No	1-2 pregnancies	Secondary & above	didn't-junior school	Community_based centres	Yes	Mild	Health complex & providers	Daily labor/Farmer	Did not access	
272	32	Khagrachhari s	16-24	Marma	Buddhist	No	No	Yes	Yes	Don't kno	No					Young (15-29 yrs)	became preg <19	Yes	10000-19000		Complete housewife		No		daily lab/f	<5km	Secondary school	Didn't attend - Primary	No	No	Yes	No	1-2 pregnancies	Secondary & above	didn't-junior school	Community_based centres	Yes	Severe (need medical attention)	Relatives & other	Daily labor/Farmer	Did not access	
273	32	Khagrachhari s	25-29	Marma	Buddhist	No	No	Yes	Yes	Don't kno	Yes	No	No	Bangla	Yes	Young (15-29 yrs)	became pregnant 20+ yrs	Yes	4000-9000	Illness/complications	Complete housewife	3rd trimesters	No	Community health w	daily lab/f	<5km	Secondary school	Secondary school	No	Yes	No	No	1-2 pregnancies	Secondary & above	didn't-junior school	Secondary hospitals	Yes	Moderate (debiliated daily life)	Health complex & providers	Daily labor/Farmer	<4 ANC	Did not pay
274	32	Khagrachhari s	25-29	Marma	Buddhist	No	No	Yes	Yes	Yes	Yes	Yes	No	Bangla		Young (15-29 yrs)	became pregnant 20+ yrs	Yes	20000 and above	Perceived appro. time	Involve in income generation	1st trimesters	No	Home visit by HW/w	Business	<5km	Secondary school	Secondary school	No	No	Yes	Yes	1-2 pregnancies	Secondary & above	secondary & above	Community_based centres	Yes	Severe (need medical attention)	Relatives & other	Employed/Business	<4 ANC	<=300 tk
275	32	Khagrachhari s	16-24	Marma	Buddhist	No	Yes	Yes	Yes	Yes	No					Young (15-29 yrs)	became preg <19	Yes	4000-9000		Complete housewife		No		daily lab/f	<5km	Secondary school	Secondary school	No	No	Yes	No	1-2 pregnancies	Secondary & above	didn't-junior school	Secondary hospitals	Yes	Mild	Relatives & other	Daily labor/Farmer	Did not access	
276	32	Khagrachhari s	16-24	Marma	Buddhist	No	No	No	Yes	Don't kno	Yes	Yes	Yes	Your own language		Young (15-29 yrs)	became preg <19	Yes	4000-9000	HW came home	Complete housewife	2nd trimesters	No	UHC or other priman	daily lab/f	<5km	Secondary school	Didn't attend - Primary	No	No	Yes	No	1-2 pregnancies	didn't-junior school	didn't-junior school	Secondary hospitals	Yes	No problem reported	Health complex & providers	Daily labor/Farmer	=>4 ANC	>300 tk
277	32	Khagrachhari s	=>30	Marma	Buddhist	No	No	Yes	No							Adult (30 & above v	became preg <19	No	4000-9000		Involve in income generation		No		daily lab/f	<5km	Didn't attend - primary	Didn't attend - Primary	No	No	No	No	3 & above pregnancies	didn't-junior school	didn't-junior school	Do not know	No	Mild		Daily labor/Farmer	Did not access	
278	32	Khagrachhari s	25-29	Marma	Buddhist	No	No	Yes	Yes	Don't kno	Yes	Yes	No	Bangla	No	Young (15-29 yrs)	became preg <19	Yes	20000 and above	Perceived appro. time	Involve in income generation	2nd trimesters	No	Private clinic/doctor	Business	<5km	Didn't attend - primary	Secondary school	No	Yes	No	No	1-2 pregnancies	didn't-junior school	secondary & above	Secondary hospitals	Yes	Mild	Health complex & providers	Employed/Business	=>4 ANC	<=300 tk
279	32	Khagrachhari s	=>30	Marma	Buddhist	No	No	Yes	Yes	Yes	Yes	Yes	Yes	Your own	No	Adult (30 & above v	became preg <19	Yes	10000-19000	Illness/complications	Involve in income generation	3rd trimesters	No	Private clinic/doctor	daily lab/f	<5km	Didn't attend - primary	Didn't attend - Primary	No	Yes	No	No	3 & above pregnancies	didn't-junior school	didn't-junior school	Secondary hospitals	Yes	Mild	Health complex & providers	Daily labor/Farmer	<4 ANC	<=300 tk
280	32	Khagrachhari s	=>30	Marma	Buddhist	No	No	Yes	No							Adult (30 & above v	became preg <19	No	4000-9000		Involve in income generation		No		daily lab/f	<5km	Didn't attend - primary	Didn't attend - Primary	No	No	No	No	3 & above pregnancies	didn't-junior school	didn't-junior school	Do not know	No	Mild		Daily labor/Farmer	Did not access	
281	32	Khagrachhari s	=>30	Marma	Buddhist	No	No	Yes	Yes	Don't kno	Yes	Yes	No	Bangla	No	Adult (30 & above v	became pregnant 20+ yrs	No	4000-9000	Know foetus condition	Complete housewife	3rd trimesters	No	Home visit by HW/w	daily lab/f	<5km	Didn't attend - primary	Didn't attend - Primary	No	No	Yes	No	1-2 pregnancies	didn't-junior school	didn't-junior school	Do not know	No	Mild	Relatives & other	Daily labor/Farmer	<4 ANC	<=300 tk
282	32	Khagrachhari s	=>30	Marma	Buddhist	No	No	Yes	Yes	Yes	Yes	Yes	No	Bangla	No	Adult (30 & above v	became pregnant 20+ yrs	Yes	4000-9000	Foetus sex	Complete housewife	3rd trimesters	No	Private clinic/doctor	Service hc	<5km	Didn't attend - primary	Secondary school	No	No	Yes	No	1-2 pregnancies	didn't-junior school	secondary & above	Private clinic	Yes	Mild	Relatives & other	Employed/Business	<4 ANC	>300 tk
283	32	Khagrachhari s	16-24	Marma	Buddhist	No	No	Yes	Yes	Yes	Yes	No	No	Bangla	Yes	Young (15-29 yrs)	became preg <19	Yes	10000-19000	Perceived appro. time	Complete housewife	2nd trimesters	No	Private clinic/doctor	Business	<5km	Secondary school	Didn't attend - Primary	No	Yes	Yes	No	1-2 pregnancies	Secondary & above	didn't-junior school	Secondary hospitals	Yes	Mild	Relatives & other	Employed/Business	<4 ANC	Did not pay
284	32	Khagrachhari s	16-24	Marma	Buddhist	No	Yes	Yes	Yes	Yes	Yes	No	No	Bangla	Yes	Young (15-29 yrs)	became preg <19	Yes	10000-19000	Illness/complications	Complete housewife	2nd trimesters	No	Private clinic/doctor	Business	<5km	Secondary school	Didn't attend - Primary	No	Yes	Yes	No	1-2 pregnancies	Secondary & above	didn't-junior school	Community_based centres	Yes	Mild	Relatives & other	Employed/Business	<4 ANC	Did not pay
285	33	Khagrachhari s	=>30	Chakma	Buddhist	No	No	Yes	Yes	Yes	Yes	Yes	No	Bangla	Yes	Adult (30 & above v	became pregnant 20+ yrs	Yes	10000-19000	Illness/complications	Involve in income generation	2nd trimesters	No	Private clinic/doctor	Service hc	6-10km	Secondary school	Secondary school	No	Yes	Yes	No	1-2 pregnancies	Secondary & above	secondary & above	Private clinic	Yes	Severe (need medical attention)	Relatives & other	Employed/Business	<4 ANC	>300 tk
286	33	Khagrachhari s	16-24	Marma	Buddhist	No	No	Yes	No							Young (15-29 yrs)	became pregnant 20+ yrs	Yes	10000-19000		Involve in income generation		No		Service hc	6-10km	Didn't attend - primary	Didn't attend - Primary	No	No	No	No	1-2 pregnancies	didn't-junior school	didn't-junior school	Secondary hospitals	Yes	Mild		Employed/Business	Did not access	
287	33	Khagrachhari s	16-24	Marma	Buddhist	No	No	Yes	No							Young (15-29 yrs)	became pregnant 20+ yrs	Yes	20000 and above		Involve in income generation		No		Service hc	6-10km	Didn't attend - primary	Secondary school	No	No	No	No	1-2 pregnancies	didn't-junior school	secondary & above	Secondary hospitals	Yes	Mild		Employed/Business	Did not access	
288	33	Khagrachhari s	25-29	Chakma	Buddhist	No	No	Yes	Yes	Don't kno	No					Young (15-29 yrs)	became preg <19	Yes	20000 and above		Involve in income generation		No		Service hc	6-10km	Secondary school	Secondary school	No	Yes	No	No	1-2 pregnancies	Secondary & above	secondary & above	Community_based centres	Yes	Mild	Health complex & providers	Employed/Business	Did not access	
289	33	Khagrachhari s	=>30	Chakma	Buddhist	Yes	No	Yes	Yes	Yes	Yes	No	No	Bangla	Yes	Adult (30 & above v	became pregnant 20+ yrs	Yes	20000 and above	Illness/complications	Involve in income generation	2nd trimesters	No	Private clinic/doctor	Service hc	6-10km	Secondary school	Secondary school	Yes	Yes	No	No	3 & above pregnancies	Secondary & above	didn't-junior school	Community_based centres	Yes	Severe (need medical attention)	Health complex & providers	Employed/Business	<4 ANC	Did not pay
290	33	Khagrachhari s	16-24	Chakma	Buddhist	No	No	Yes	No							Young (15-29 yrs)	became preg <19	Yes	4000-9000		Involve in income generation		No		daily lab/f	6-10km	Didn't attend - primary	Didn't attend - Primary	No	No	No	No	1-2 pregnancies	didn't-junior school	didn't-junior school	Secondary hospitals	Yes	Moderate (debiliated daily life)		Daily labor/Farmer	Did not access	
291	33	Khagrachhari s	16-24	Chakma	Buddhist	No	No	No	Yes	Don't kno	Yes	No	No	Bangla	Yes	Young (15-29 yrs)	became preg <19	Yes	20000 and above	HW came home	Complete housewife	2nd trimesters	No	UHC or other priman	Service hc	6-10km	Secondary school	Higher secondary & above	No	Yes	Yes	No	1-2 pregnancies	Secondary & above	secondary & above	Secondary hospitals	Yes	No problem reported	Relatives & other	Employed/Business	<4 ANC	Did not pay
292	33	Khagrachhari s	25-29	Chakma	Buddhist	No	No	Yes	No							Young (15-29 yrs)	became pregnant 20+ yrs	Yes	10000-19000		Involve in income generation		No		Service hc	6-10km	Didn't attend - primary	Secondary school	No	No	No	No	1-2 pregnancies	didn't-junior school	secondary & above	Secondary hospitals	Yes	Mild		Employed/Business	Did not access	
293	33	Khagrachhari s	16-24	Chakma	Buddhist	No	No	Yes	No							Young (15-29 yrs)	became preg <19	Yes	4000-9000		Involve in income generation		No		daily lab/f	6-10km	Didn't attend - primary	Secondary school	No	No	No	No	1-2 pregnancies	didn't-junior school	didn't-junior school	Secondary hospitals	Yes	Mild		Daily labor/Farmer	Did not access	
294	33	Khagrachhari s	16-24	Chakma	Buddhist	No	No	Yes	Yes	Don't kno	Yes	No	No	Bangla	No	Young (15-29 yrs)	became preg <19	Yes	4000-9000	Illness/complications	Involve in income generation	3rd trimesters	No	Home visit by HW/w	Service hc	6-10km	Didn't attend - primary	Secondary school	No	Yes	No	No	1-2 pregnancies	didn't-junior school	didn't-junior school	Secondary hospitals	Yes	Severe (need medical attention)	Health complex & providers	Employed/Business	<4 ANC	Did not pay
295	33	Khagrachhari s	16-24	Chakma	Buddhist	No	No	Yes	Yes	Don't kno	Yes	No	No	Bangla	No	Young (15-29 yrs)	became preg <19	Yes	10000-19000	Illness/complications	Involve in income generation	2nd trimesters	No	UHC or other priman	Service hc	6-10km	Secondary school	Secondary school	No	Yes	Yes	No	1-2 pregnancies	didn't-junior school	didn't-junior school	Secondary hospitals	Yes	Mild	Relatives & other	Employed/Business	<4 ANC	Did not pay
296	33	Khagrachhari s	25-29	Chakma	Buddhist	No	No	No	No							Young (15-29 yrs)	became preg <19	No	4000-9000		Involve in income generation		No		daily lab/f	6-10km	Didn't attend - primary	Didn't attend - Primary	No	No	No	No	3 & above pregnancies	didn't-junior school	didn't-junior school	Do not know	No	No problem reported		Daily labor/Farmer	Did not access	
297	34	Matiranga	=>30	Chakma	Buddhist	No	No	No	Yes	Don't kno	No					Adult (30 & above v	became pregnant 20+ yrs	No	4000-9000		Involve in income generation		No		daily lab/f	>10 km	Didn't attend - primary	Didn't attend - Primary	No	No	Yes	No	3 & above pregnancies	didn't-junior school	didn't-junior school	Do not know	No	No problem reported	Relatives & other	Daily labor/Farmer	Did not access	
298	34	Matiranga	25-29	Chakma	Buddhist	No	No	Yes	Yes	Yes	Yes	No	No	Bangla	Yes	Young (15-29 yrs)	became pregnant 20+ yrs	Yes	20000 and above	Confirm pregnancy	Complete housewife	1st trimesters	No	UHC or other priman	Service hc	>10 km	Secondary school	Secondary school	No	Yes	No	No	1-2 pregnancies	Secondary & above	secondary & above	Secondary hospitals	Yes	Mild	Health complex & providers	Employed/Business	<4 ANC	Did not pay
299	34	Matiranga	16-24	Chakma	Buddhist	No	No	Yes	No							Young (15-29 yrs)	became preg <19	Yes	4000-9000		Involve in income generation		No		daily lab/f	>10 km	Didn't attend - primary	Didn't attend - Primary	No	No	No	No	1-2 pregnancies	didn't-junior school	didn't-junior school	Secondary hospitals	Yes	No problem reported		Daily labor/Farmer	Did not access	
300	34	Matiranga	16-24	Chakma	Buddhist	No	No	No	Yes	Don't kno	No					Young (15-29 yrs)	became preg <19	Yes	4000-9000		Involve in income generation		No		daily lab/f	>10 km	Didn't attend - primary	Didn't attend - Primary	No	No	Yes	No	1-2 pregnancies	didn't-junior school	didn't-junior school	Secondary hospitals	Yes	No problem reported	Relatives & other	Daily labor/Farmer	Did not access	
301	34	Matiranga	=>30	Chakma	Buddhist	No	No	Yes	Yes	Yes	Yes	Yes	No	Bangla	Yes	Adult (30 & above v	became preg <19	Yes	20000 and above	Confirm pregnancy	Complete housewife	1st trimesters	Yes	Private clinic/doctor	Business	>10 km	Secondary school	Secondary school	No	Yes	No	No	1-2 pregnancies	Secondary & above	secondary & above	Community_based centres	Yes	Moderate (debiliated daily life)	Health complex & providers	Employed/Business	<4 ANC	>300 tk
302	34	Matiranga	=>30	Chakma	Buddhist	No	Yes	Yes	Yes	Yes	Yes	Yes	No	Bangla	Yes	Adult (30 & above v	became pregnant 20+ yrs	Yes	20000 and above	Perceived appro. time	Involve in income generation	2nd trimesters	No	Private clinic/doctor	Business	>10 km	Secondary school	Secondary school	Yes	Yes	No	No	1-2 pregnancies	Secondary & above	secondary & above	Secondary hospitals	Yes	Severe (need medical attention)	Health complex & providers	Employed/Business	=>4 ANC	>300 tk
303	34	Matiranga	16-24	Chakma	Buddhist	No	No	No	Yes	Yes	Yes	No	No	Bangla	No	Young (15-29 yrs)	became preg <19	Yes	4000-9000	Perceived appro. time	Involve in income generation	2nd trimesters	No	UHC or other priman	Service hc	>10 km	Didn't attend - primary	Secondary school	No	No	Yes	No	1-2 pregnancies	didn't-junior school	secondary & above	Secondary hospitals	Yes	No problem reported	Relatives & other	Employed/Business	<4 ANC	Did not pay
304	34	Matiranga	=>30	Chakma	Buddhist	Yes	Yes	Yes	Yes	Yes	No					Adult (30 & above v	became pregnant 20+ yrs	Yes	10000-19000		Involve in income generation		No		Service hc	>10 km	Secondary school	Secondary school	Yes	Yes	No	No	3 & above pregnancies	Secondary & above	secondary & above	Private clinic	Yes	Mild	Health complex & providers	Employed/Business	Did not access	
305	34	Matiranga	16-24	Chakma	Buddhist	No	No	No	No							Young (15-29 yrs)	became pregnant 20+ yrs	No	10000-19000		Involve in income generation		No		daily lab/f	>10 km	Secondary school	Didn't attend - Primary	No	No	No	No	1-2 pregnancies	didn't-junior school	didn't-junior school	Do not know	No	No problem reported		Daily labor/Farmer	Did not access	
306	34	Matiranga	16-24	Chakma	Buddhist	No	No	Yes	Yes	Don't kno	No					Young (15-29 yrs)	became preg <19	Yes	10000-19000		Involve in income generation		No		Service hc	>10 km	Secondary school	Secondary school	No	No	Yes	No	1-2 pregnancies	didn't-junior school	secondary & above	Community_based centres	Yes	Mild	Relatives & other	Employed/Business	Did not access	
307	34	Matiranga	16-24	Chakma	Buddhist	Yes	No	Yes	Yes	Yes	Yes	No	No	Bangla	No	Young (15-29 yrs)	became pregnant 20+ yrs	Yes	10000-19000	Know foetus condition	Involve in income generation	2nd trimesters	No	UHC or other priman	Service hc	>10 km	Secondary school	Secondary school	Yes	Yes	No	No	1-2 pregnancies	Secondary & above	secondary & above	Secondary hospitals	Yes	Severe (need medical attention)	Health complex & providers	Employed/Business	<4 ANC	Did not pay
308	34	Matiranga	=>30	Chakma	Buddhist	Yes	No	Yes	Yes	Yes	No					Adult (30 & above v	became pregnant 20+ yrs	Yes	10000-19000		Involve in income generation		No		Service hc	>10 km	Secondary school	Secondary school	No	No	Yes	No	3 & above pregnancies	didn't-junior school	didn't-junior school	Community_based centres	Yes	Mild	Relatives & other	Employed/Business	Did not access	
309	34	Matiranga	16-24	Chakma	Buddhist	No	No	Yes	No							Young (15-29 yrs)	became preg <19	Yes	10000-19000		Involve in income generation		No		Service hc	>10 km	Didn't attend - primary	Secondary school	No	No	No	No	1-2 pregnancies	didn't-junior school	didn't-junior school	Secondary hospitals	Yes	Mild		Employed/Business	Did not access	
310	35	Matiranga	16-24	Chakma	Buddhist	No	Yes	No	No							Young (15-29 yrs)	became preg <19	No	4000-9000		Complete housewife		No		daily lab/f	6-10km	Didn't attend - primary	Secondary school	No	No	No	No	1-2 pregnancies	didn't-junior school	didn't-junior school	Do not know	No	No problem reported		Daily labor/Farmer	Did not access	
311	35	Matiranga	25-29	Chakma	Buddhist	Yes	No	Yes	Yes	Don't kno	No					Young (15-29 yrs)	became preg <19	Yes	10000-19000		Involve in income generation		No		Service hc	6-10km	Didn't attend - primary	Secondary school	No	No	Yes	No	3 & above pregnancies	didn't-junior school	secondary & above	Secondary hospitals	Yes	Severe (need medical attention)	Relatives & other	Employed/Business	Did not access	
312	35	Matiranga	16-24	Chakma	Buddhist	Yes	No	No	Yes	Don't kno	No					Young (15-29 yrs)	became preg <19	Yes	4000-9000		Involve in income generation		No		daily lab/f	6-10km	Didn't attend - primary	Didn't attend - Primary	No	No	Yes	No	3 & above pregnancies	didn't-junior school	didn't-junior school	Community_based centres	Yes	No problem reported	Relatives & other	Daily labor/Farmer	Did not access	
313	35	Matiranga	=>30	Chakma	Buddhist	Yes	No	Yes	Yes	Don't kno	Yes	No	No	Bangla	No	Adult (30 & above v	became pregnant 20+ yrs	Yes	4000-9000	Illness/complications	Involve in income generation	2nd trimesters	No	Private clinic/doctor	Service hc	6-10km	Didn't attend - primary	Didn't attend - Primary	No	Yes	No	No	3 & above pregnancies	didn't-junior school	didn't-junior school	Secondary hospitals	Yes	Severe (need medical attention)	Health complex & providers	Employed/Business	<4 ANC	Did not pay
314	35	Matiranga	25-29	Chakma	Buddhist	No	No	No	No							Young (15-29 yrs)	became pregnant 20+ yrs	Yes	4000-9000		Involve in income generation		No		Service hc	6-10km	Didn't attend - primary	Secondary school	No	No	No	No	3 & above pregnancies	didn't-junior school	didn't-junior school	Secondary hospitals	Yes	No problem reported		Employed/Business	Did not access	
315	35	Matiranga	25-29	Chakma	Buddhist	No	No	Yes	No							Young (15-29 yrs)	became pregnant 20+ yrs	Yes	10000-19000		Involve in income generation		No		Service hc	6-10km	Secondary school	Didn't attend - Primary	No	No	No	No	1-2 pregnancies	didn't-junior school	didn't-junior school	Community_based centres	Yes	Severe (need medical attention)		Employed/Business	Did not access	
316	35	Matiranga	16-24	Chakma	Buddhist	Yes	No	Yes	No							Young (15-29 yrs)	became preg <19	Yes	4000-9000		Involve in income generation		No		Service hc	6-10km	Didn't attend - primary	Secondary school	No	No	No	No	1-2 pregnancies	didn't-junior school	secondary & above	Secondary hospitals	Yes	Severe (need medical attention)		Employed/Business	Did not access	
317	35	Matiranga	16-24	Chakma	Buddhist	No	Yes	Yes	Yes	Yes	Yes	Yes	No	Bangla	No	Young (15-29 yrs)	became pregnant 20+ yrs	Yes	10000-19000	Foetus sex	Complete housewife	2nd trimesters	No	Private clinic/doctor	Service hc	6-10km	Didn't attend - primary	Higher secondary & above	No	Yes	Yes	Yes	1-2 pregnancies	didn't-junior school	secondary & above	Private clinic	Yes	Severe (need medical attention)	Relatives & other	Employed/Business	<4 ANC	>300 tk
318	35	Matiranga	25-29	Chakma	Buddhist	No	Yes	Yes	Yes	Yes	Yes	Yes	No	Bangla	No	Young (15-29 yrs)	became preg <19	Yes	10000-19000	Confirm pregnancy	Involve in income generation	1st trimesters	No	Private clinic/doctor	Service hc	6-10km	Didn't attend - primary	Didn't attend - Primary	No	Yes	No	No	3 & above pregnancies	didn't-junior school	didn't-junior school	Secondary hospitals	Yes	Severe (need medical attention)	Health complex & providers	Employed/Business	<4 ANC	<=300 tk
319	36	Matiranga	25-29	Chakma	Buddhist	Yes	No	Yes	Yes	Yes	Yes	No	No	Bangla	Yes	Young (15-29 yrs)	became pregnant 20+ yrs	Yes	10000-19000	Know foetus condition	Involve in income generation	2nd trimesters	No	UHC or other priman	Service hc	>10 km	Higher secondary & above	Secondary school	No	No	Yes	No	1-2 pregnancies	Secondary & above	secondary & above	Private clinic	Yes	Mild	Relatives & other	Employed/Business	=>4 ANC	Did not pay
320	36	Matiranga	16-24	Chakma	Buddhist	Yes	No	Yes	Yes	Yes	Yes	Yes	No	Bangla	Yes	Young (15-29 yrs)	became preg <19	Yes	20000 and above	Confirm pregnancy	Complete housewife	1st trimesters	No	Private clinic/doctor	Service hc	>10 km	Higher secondary & above	Higher secondary & above	No	No	Yes	No	1-2 pregnancies	Secondary & above	secondary & above	Private clinic	Yes	Severe (need medical attention)	Relatives & other	Employed/Business	=>4 ANC	>300 tk
321	36	Matiranga	16-24	Chakma	Buddhist	No	No	No	Yes	Yes	Yes	No	No	Bangla	No	Young (15-29 yrs)	became preg <19	Yes	10000-19000	Perceived appro. time	Involve in income generation	2nd trimesters	No	UHC or other priman	Service hc	>10 km	Secondary school	Secondary school	No	No	Yes	No	1-2 pregnancies	didn't-junior school	didn't-junior school	Private clinic	Yes	No problem reported	Relatives & other	Employed/Business	=>4 ANC	Did not pay
322	36	Matiranga	16-24	Chakma	Buddhist	No	No	Yes	Yes	Yes	Yes	No	No	Bangla	No	Young (15-29 yrs)	became pregnant 20+ yrs	Yes	4000-9000	Illness/complications	Involve in income generation	2nd trimesters	No	Private clinic/doctor	daily lab/f	>10 km	Didn't attend - primary	Didn't attend - Primary	No	No	Yes	No	1-2 pregnancies	didn't-junior school	didn't-junior school	Secondary hospitals	Yes	Mild	Relatives & other	Daily labor/Farmer	<4 ANC	Did not pay
323	37	Matiranga	16-24	Chakma	Buddhist	No	No	Yes	No							Young (15-29 yrs)	became preg <19	Yes	4000-9000		Involve in income generation		No		Business	>10 km	Didn't attend - primary	Didn't attend - Primary	No	No	No	No	1-2 pregnancies	didn't-junior school	didn't-junior school	Secondary hospitals	Yes	Severe (need medical attention)		Employed/Business	Did not access	
324	37	Matiranga	16-24	Chakma	Buddhist	Yes	No	Yes	No							Young (15-29 yrs)	became preg <19	Yes	4000-9000		Involve in income generation		No		Service hc	>10 km	Didn't attend - primary	Didn't attend - Primary	No	No	No	No	3 & above pregnancies	didn't-junior school	didn't-junior school	Secondary hospitals	Yes	Mild		Employed/Business	Did not access	
325	37	Matiranga	16-24	Chakma	Buddhist	Yes	No	Yes	Yes	Yes	No					Young (15-29 yrs)	became pregnant 20+ yrs	Yes	20000 and above		Complete housewife		No		Service hc	>10 km	Secondary school	Secondary school	No	No	Yes	No	1-2 pregnancies	didn't-junior school	secondary & above	Secondary hospitals	Yes	Mild	Relatives & other	Employed/Business	Did not access	
326	37	Matiranga	25-29	Chakma	Buddhist	No	No	Yes	Yes	Don't kno	Yes	No	No	Bangla	No	Young (15-29 yrs)	became preg <19	No	4000-9000	Perceived appro. time	Involve in income generation	2nd trimesters	No	Private clinic/doctor	daily lab/f	>10 km	Didn't attend - primary	Secondary school	No	Yes	Yes	No	1-2 pregnancies	didn't-junior school	didn't-junior school	Do not know	No	Mild	Relatives & other	Daily labor/Farmer	<4 ANC	Did not pay
327	38	Matiranga	16-24	Chakma	Buddhist	No	No	Yes	No							Young (15-29 yrs)	became preg <19	Yes	10000-19000		Involve in income generation		No		Service hc	>10 km	Secondary school	Didn't attend - Primary	No	No	No	No	1-2 pregnancies	didn't-junior school	didn't-junior school	Secondary hospitals	Yes	Mild		Employed/Business	Did not access	
328	38	Matiranga	16-24	Chakma	Buddhist	No	No	Yes	Yes	Don't kno	No					Young (15-29 yrs)	became preg <19	Yes	4000-9000		Involve in income generation		No		daily lab/f	>10 km	Didn't attend - primary	Didn't attend - Primary	No	Yes	No	No	1-2 pregnancies	didn't-junior school	didn't-junior school	Community_based centres	Yes	Mild	Health complex & providers	Daily labor/Farmer	Did not access	
329	38	Matiranga	25-29	Chakma	Buddhist	Yes	Yes	No	Yes	Yes	No					Young (15-29 yrs)	became preg <19	Yes	10000-19000		Involve in income generation		No		daily lab/f	>10 km	Secondary school	Didn't attend - Primary	No	Yes	No	No	3 & above pregnancies	didn't-junior school	didn't-junior school	Community_based centres	Yes	No problem reported	Health complex & providers	Daily labor/Farmer	Did not access	
330	38	Matiranga	=>30	Chakma	Buddhist	Yes	No	Yes	No							Adult (30 & above v	became preg <19	Yes	4000-9000		Involve in income generation		No		daily lab/f	>10 km	Didn't attend - primary	Didn't attend - Primary	No	No	No	No	3 & above pregnancies	didn't-junior school	didn't-junior school	Community_based centres	Yes	Severe (need medical attention)		Daily labor/Farmer	Did not access	
331	38	Matiranga	16-24	Chakma	Buddhist	No	No	Yes	Yes	Yes	Yes	No	No	Bangla	No	Young (15-29 yrs)	became preg <19	Yes	20000 and above	Perceived appro. time	Complete housewife	2nd trimesters	No	Home visit by HW/w	Business	>10 km	Secondary school	Secondary school	No	No	Yes	No	1-2 pregnancies	Secondary & above	secondary & above	Private clinic	Yes	Severe (need medical attention)	Relatives & other	Employed/Business	<4 ANC	Did not pay
332	38	Matiranga	=>30	Chakma	Buddhist	No	No	Yes	Yes	Yes	Yes	Yes	No	Bangla	No	Adult (30 & above v	became preg <19	Yes	20000 and above	Confirm pregnancy	Involve in income generation	2nd trimesters	No	Community health w	Service hc	>10 km	Didn't attend - primary	Didn't attend - Primary	Yes	No	Yes	No	3 & above pregnancies	didn't-junior school	didn't-junior school	Community_based centres	Yes	Mild	Relatives & other	Employed/Business	<4 ANC	>300 tk
333	38	Matiranga	16-24	Chakma	Buddhist	No	No	Yes	Yes	Yes	No					Young (15-29 yrs)	became preg <19	Yes	10000-19000		Involve in income generation		No		daily lab/f	>10 km	Secondary school	Higher secondary & above	No	Yes	No	No	1-2 pregnancies	Secondary & above	secondary & above	Community_based centres	Yes	Mild	Health complex & providers	Daily labor/Farmer	Did not access	
334	38	Matiranga	=>30	Chakma	Buddhist	Yes	Yes	Yes	Yes	Yes	Yes	No	Yes	Your own language		Adult (30 & above v	became preg <19	Yes	20000 and above	Perceived appro. time	Involve in income generation	2nd trimesters	No	Home visit by HW/w	Service hc	>10 km	Secondary school	Secondary school	No	Yes	No	No	3 & above pregnancies	Secondary & above	secondary & above	Secondary hospitals	Yes	Severe (need medical attention)	Health complex & providers	Employed/Business	=>4 ANC	Did not pay
335	38	Matiranga	25-29	Chakma	Buddhist	No	No	No	Yes	Yes	Yes	No	No	Bangla	Yes	Young (15-29 yrs)	became pregnant 20+ yrs	Yes	20000 and above	Perceived appro. time	Involve in income generation	2nd trimesters	No	Home visit by HW/w	Business	>10 km	Secondary school	Secondary school	No	Yes	No	No	1-2 pregnancies	didn't-junior school	secondary & above	Community_based centres	Yes	No problem reported	Health complex & providers	Employed/Business	<4 ANC	Did not pay
336	38	Matiranga	16-24	Chakma	Buddhist	No	No	No	No							Young (15-29 yrs)	became preg <19	No	4000-9000		Involve in income generation		No		daily lab/f	>10 km	Didn't attend - primary	Didn't attend - Primary	No	No	No	No	1-2 pregnancies	didn't-junior school	didn't-junior school	Do not know	No	No problem reported		Daily labor/Farmer	Did not access	
337	38	Matiranga	25-29	Chakma	Buddhist	No	No	Yes	Yes	Don't kno	No					Young (15-29 yrs)	became preg <19	No	4000-9000		Involve in income generation		No		daily lab/f	>10 km	Didn't attend - primary	Secondary school	No	No	Yes	No	3 & above pregnancies	didn't-junior school	didn't-junior school	Do not know	No	Severe (need medical attention)	Relatives & other	Daily labor/Farmer	Did not access	
338	38	Matiranga	16-24	Chakma	Buddhist	No	No	Yes	No							Young (15-29 yrs)	became preg <19	No	4000-9000		Involve in income generation		No		daily lab/f	>10 km	Didn't attend - primary	Didn't attend - Primary	No	No	No	No	1-2 pregnancies	didn't-junior school	didn't-junior school	Do not know	No	Mild		Daily labor/Farmer	Did not access	
339	38	Matiranga	16-24	Chakma	Buddhist	Yes	No	Yes	Yes	Don't kno	No					Young (15-29 yrs)	became preg <19	Yes	4000-9000		Involve in income generation		No		daily lab/f	>10 km	Didn't attend - primary	Secondary school	No	No	Yes	No	3 & above pregnancies	didn't-junior school	didn't-junior school	Community_based centres	Yes	Mild	Relatives & other	Daily labor/Farmer	Did not access	
340	38	Matiranga	16-24	Chakma	Buddhist	No	No	Yes	No							Young (15-29 yrs)	became preg <19	No	4000-9000		Involve in income generation		No		daily lab/f	>10 km	Didn't attend - primary	Secondary school	No	No	No	No	1-2 pregnancies	didn't-junior school	secondary & above	Do not know	No	Severe (need medical attention)		Daily labor/Farmer	Did not access	
341	38	Matiranga	16-24	Chakma	Buddhist	Yes	No	Yes	No							Young (15-29 yrs)	became preg <19	No	4000-9000		Involve in income generation		No		daily lab/f	>10 km	Didn't attend - primary	Didn't attend - Primary	No	No	No	No	1-2 pregnancies	didn't-junior school	didn't-junior school	Do not know	No	Mild		Daily labor/Farmer	Did not access	
342	38	Matiranga	=>30	Chakma	Buddhist	Yes	No	Yes	No							Adult (30 & above v	became preg <19	No	4000-9000		Involve in income generation		No		daily lab/f	>10 km	Didn't attend - primary	Didn't attend - Primary	No	No	No	No	3 & above pregnancies	didn't-junior school	didn't-junior school	Do not know	No	Severe (need medical attention)		Daily labor/Farmer	Did not access	
343	38	Matiranga	25-29	Chakma	Buddhist	Yes	No	Yes	No							Young (15-29 yrs)	became preg <19	No	20000 and above		Involve in income generation		No		Service hc	>10 km	Didn't attend - primary	Secondary school	No	No	No	No	3 & above pregnancies	didn't-junior school	secondary & above	Do not know	No	Mild		Employed/Business	Did not access	
344	38	Matiranga	16-24	Chakma	Buddhist	Yes	Yes	Yes	Yes	Yes	Yes	Yes	No	Bangla	No	Young (15-29 yrs)	became preg <19	Yes	20000 and above	Perceived appro. time	Complete housewife	2nd trimesters	No	Private clinic/doctor	daily lab/f	>10 km	Secondary school	Higher secondary & above	No	Yes	No	No	1-2 pregnancies	Secondary & above	secondary & above	Private clinic	Yes	Mild	Health complex & providers	Daily labor/Farmer	=>4 ANC	>300 tk
345	38	Matiranga	25-29	Chakma	Buddhist	Yes	No	Yes	No							Young (15-29 yrs)	became preg <19	No	4000-9000		Involve in income generation		No		daily lab/f	>10 km	Didn't attend - primary	Secondary school	No	No	No	No	3 & above pregnancies	didn't-junior school	didn't-junior school	Do not know	No	Moderate (debiliated daily life)		Daily labor/Farmer	Did not access	
346	38	Matiranga	16-24	Chakma	Buddhist	No	No	Yes	Yes	Don't kno	Yes	Yes	No	Bangla	No	Young (15-29 yrs)	became preg <19	No	20000 and above	Illness/complications	Involve in income generation	3rd trimesters	No	Private clinic/doctor	Service hc	>10 km	Secondary school	Secondary school	No	No	Yes	No	1-2 pregnancies	Secondary & above	secondary & above	Do not know	No	Severe (need medical attention)	Relatives & other	Employed/Business	<4 ANC	>300 tk
347	38	Matiranga	=>30	Chakma	Buddhist	No	No	No	No							Adult (30 & above v	became preg <19	Yes	4000-9000		Involve in income generation		No		daily lab/f	>10 km	Didn't attend - primary	Didn't attend - Primary	No	No	No	No	3 & above pregnancies	didn't-junior school	didn't-junior school	Community_based centres	Yes	No problem reported		Daily labor/Farmer	Did not access	
348	38	Matiranga	16-24	Chakma	Buddhist	No	No	Yes	No							Young (15-29 yrs)	became preg <19	No	4000-9000		Involve in income generation		No		daily lab/f	>10 km	Didn't attend - primary	Didn't attend - Primary	No	No	No	No	1-2 pregnancies	didn't-junior school	didn't-junior school	Do not know	No	Mild		Daily labor/Farmer	Did not access	
349	39	Matiranga	=>30	Marma	Buddhist	Yes	Yes	Yes	Yes	Yes	Yes	No	No	Bangla	Yes	Adult (30 & above v	became pregnant 20+ yrs	Yes	20000 and above	Perceived appro. time	Involve in income generation	2nd trimesters	Yes	Home visit by HW/w	Business	<5km	Higher secondary & above	Secondary school	Yes	Yes	Yes	Yes	3 & above pregnancies	Secondary & above	didn't-junior school	Secondary hospitals	Yes	Severe (need medical attention)	Relatives & other	Employed/Business	=>4 ANC	Did not pay
350	39	Matiranga	25-29	Marma	Buddhist	No	No	Yes	Yes	Yes	Yes	No	No	Bangla	Yes	Young (15-29 yrs)	became pregnant 20+ yrs	Yes	20000 and above	Illness/complications	Complete housewife	2nd trimesters	Yes	Home visit by HW/w	Business	<5km	Higher secondary & above	Secondary school	No	Yes	No	No	1-2 pregnancies	Secondary & above	secondary & above	Secondary hospitals	Yes	Severe (need medical attention)	Health complex & providers	Employed/Business	<4 ANC	Did not pay
351	39	Matiranga	=>30	Marma	Buddhist	No	No	Yes	Yes	Yes	Yes	No	No	Bangla	No	Adult (30 & above v	became pregnant 20+ yrs	Yes	20000 and above	Perceived appro. time	Complete housewife	2nd trimesters	Yes	Home visit by HW/w	Business	<5km	Secondary school	Secondary school	No	Yes	No	No	1-2 pregnancies	Secondary & above	secondary & above	Secondary hospitals	Yes	Mild	Health complex & providers	Employed/Business	=>4 ANC	Did not pay

352	39	Matiranga	25-29	Marma	Buddhist	No	No	Yes	Yes	Yes	Yes	No	Yes	Bangla	Yes	Young (15-29 yrs)	became pregnant 20+ yrs	Yes	20000 and above	Confirm pregnancy	Complete housewife	1st trimesters	Yes	Govt Health Comple	daily lab/f	<5km	Higher secondary & above	Secondary school	No	No	Yes	No	1-2 pregnancies	Secondary & above	secondary & above	Private clinic	Yes	Mild	Relatives & other	Daily labor/Farmer	=>4 ANC	Did not pay
353	39	Matiranga	=>30	Marma	Buddhist	No	No	No	No							Adult (30 & above v	became pregnant 20+ yrs	Yes	4000-9000		Complete housewife		No		daily lab/f	<5km	Didn't attend - primary	Didn't attend - Primary	No	No	No	No	1-2 pregnancies	didn't-junior school	didn't-junior school	Secondary hospitals	Yes	No problem reported		Daily labor/Farmer	Did not access	
354	39	Matiranga	16-24	Marma	Buddhist	No	No	Yes	Yes	Yes	Yes	Yes	No	Bangla	Yes	Young (15-29 yrs)	became pregnant 20+ yrs	Yes	20000 and above	Perceived appro. time	Complete housewife	2nd trimesters	No	Govt Health Comple	Service hc	<5km	Higher secondary & above	Higher secondary & above	No	Yes	Yes	No	1-2 pregnancies	Secondary & above	secondary & above	Private clinic	Yes	Mild	Relatives & other	Employed/Business	=>4 ANC	>300 tk
355	39	Matiranga	16-24	Marma	Buddhist	Yes	No	Yes	Yes	Don't kno	No					Young (15-29 yrs)	became preg <19	Yes	20000 and above		Involve in income generation		No		Service hc	<5km	Didn't attend - primary	Secondary school	No	No	Yes	No	3 & above pregnancies	didn't-junior school	secondary & above	Community_based centres	Yes	Severe (need medical attention)	Relatives & other	Employed/Business	Did not access	
356	39	Matiranga	16-24	Marma	Buddhist	Yes	No	Yes	No							Young (15-29 yrs)	became preg <19	Yes	10000-19000		Complete housewife		No		daily lab/f	<5km	Didn't attend - primary	Didn't attend - Primary	No	No	No	No	1-2 pregnancies	didn't-junior school	didn't-junior school	Secondary hospitals	Yes	Mild		Daily labor/Farmer	Did not access	
357	39	Matiranga	16-24	Marma	Buddhist	No	Yes	Yes	Yes	Yes	Yes	No	No	Bangla	Yes	Young (15-29 yrs)	became pregnant 20+ yrs	Yes	20000 and above	Illness/complications	Involve in income generation	1st trimesters	No	Govt Health Comple	Service hc	<5km	Higher secondary & above	Secondary school	No	Yes	Yes	No	1-2 pregnancies	Secondary & above	secondary & above	Community_based centres	Yes	Moderate (debiliated daily life)	Relatives & other	Employed/Business	=>4 ANC	Did not pay
358	40	Matiranga	16-24	Chakma	Buddhist	No	Yes	Yes	Yes	Yes	Yes	No	No	Bangla	No	Young (15-29 yrs)	became preg <19	Yes	10000-19000	HW came home	Involve in income generation	2nd trimesters	No	Community health w	Service hc	>10 km	Secondary school	Didn't attend - Primary	No	Yes	No	No	1-2 pregnancies	Secondary & above	didn't-junior school	Community_based centres	Yes	Severe (need medical attention)	Health complex & providers	Employed/Business	<4 ANC	Did not pay
359	40	Matiranga	=>30	Chakma	Buddhist	Yes	No	Yes	Yes	Yes	Yes	No	Yes	Your own language		Adult (30 & above v	became preg <19	Yes	4000-9000	Illness/complications	Involve in income generation	2nd trimesters	No	Community health w	Service hc	>10 km	Didn't attend - primary	Didn't attend - Primary	No	Yes	No	No	3 & above pregnancies	didn't-junior school	didn't-junior school	Community_based centres	Yes	Mild	Health complex & providers	Employed/Business	<4 ANC	Did not pay
360	40	Matiranga	16-24	Chakma	Buddhist	No	No	Yes	Yes	Don't kno	Yes	No	No	Bangla	No	Young (15-29 yrs)	became preg <19	No	4000-9000	Illness/complications	Involve in income generation	3rd trimesters	No	Community health w	daily lab/f	>10 km	Didn't attend - primary	Didn't attend - Primary	No	No	Yes	No	1-2 pregnancies	didn't-junior school	didn't-junior school	Do not know	No	Mild	Relatives & other	Daily labor/Farmer	<4 ANC	Did not pay
361	40	Matiranga	25-29	Chakma	Buddhist	Yes	No	Yes	Yes	Don't kno	Yes	No	No	Bangla	No	Young (15-29 yrs)	became pregnant 20+ yrs	No	10000-19000	HW came home	Complete housewife	2nd trimesters	No	Community health w	Service hc	>10 km	Secondary school	Secondary school	No	Yes	No	No	3 & above pregnancies	didn't-junior school	didn't-junior school	Do not know	No	Mild	Health complex & providers	Employed/Business	<4 ANC	Did not pay
362	40	Matiranga	16-24	Chakma	Buddhist	No	No	Yes	No							Young (15-29 yrs)	became pregnant 20+ yrs	No	10000-19000		Involve in income generation		No		Service hc	>10 km	Didn't attend - primary	Didn't attend - Primary	No	No	No	No	1-2 pregnancies	didn't-junior school	didn't-junior school	Do not know	No	Severe (need medical attention)		Employed/Business	Did not access	
363	40	Matiranga	16-24	Chakma	Buddhist	No	No	No	Yes	Don't kno	Yes	No	No	Bangla	No	Young (15-29 yrs)	became pregnant 20+ yrs	Yes	4000-9000	HW came home	Involve in income generation	2nd trimesters	No	Community health w	daily lab/f	>10 km	Didn't attend - primary	Didn't attend - Primary	No	Yes	No	No	1-2 pregnancies	didn't-junior school	didn't-junior school	Secondary hospitals	Yes	No problem reported	Health complex & providers	Daily labor/Farmer	<4 ANC	Did not pay
364	40	Matiranga	25-29	Chakma	Buddhist	Yes	No	Yes	Yes	Yes	Yes	No	No	Bangla	No	Young (15-29 yrs)	became preg <19	Yes	10000-19000	Know foetus condition	Involve in income generation	3rd trimesters	No	UHC or other priman	Service hc	>10 km	Didn't attend - primary	Didn't attend - Primary	No	Yes	No	No	3 & above pregnancies	didn't-junior school	didn't-junior school	Community_based centres	Yes	Severe (need medical attention)	Health complex & providers	Employed/Business	<4 ANC	Did not pay
365	40	Matiranga	25-29	Chakma	Buddhist	Yes	No	Yes	Yes	Don't kno	No					Young (15-29 yrs)	became preg <19	Yes	4000-9000		Involve in income generation		No		daily lab/f	>10 km	Didn't attend - primary	Didn't attend - Primary	No	No	Yes	No	1-2 pregnancies	didn't-junior school	didn't-junior school	Community_based centres	Yes	Severe (need medical attention)	Relatives & other	Daily labor/Farmer	Did not access	
366	40	Matiranga	=>30	Chakma	Buddhist	Yes	No	No	Yes	Don't kno	No					Adult (30 & above v	became preg <19	Yes	10000-19000		Involve in income generation		No		daily lab/f	>10 km	Didn't attend - primary	Didn't attend - Primary	No	No	Yes	No	3 & above pregnancies	didn't-junior school	didn't-junior school	Secondary hospitals	Yes	No problem reported	Relatives & other	Daily labor/Farmer	Did not access	
367	40	Matiranga	16-24	Chakma	Buddhist	No	No	No	Yes	Yes	Yes	No	No	Bangla	Yes	Young (15-29 yrs)	became preg <19	Yes	10000-19000	Know foetus condition	Involve in income generation	2nd trimesters	No	Private clinic/doctor	Service hc	>10 km	Didn't attend - primary	Didn't attend - Primary	No	Yes	No	No	1-2 pregnancies	didn't-junior school	didn't-junior school	Community_based centres	Yes	No problem reported	Health complex & providers	Employed/Business	<4 ANC	Did not pay
368	40	Matiranga	16-24	Chakma	Buddhist	No	No	Yes	Yes	Yes	No					Young (15-29 yrs)	became preg <19	Yes	4000-9000		Involve in income generation		No		daily lab/f	>10 km	Didn't attend - primary	Didn't attend - Primary	No	Yes	No	No	1-2 pregnancies	didn't-junior school	didn't-junior school	Secondary hospitals	Yes	Mild	Health complex & providers	Daily labor/Farmer	Did not access	
369	40	Matiranga	16-24	Chakma	Buddhist	No	No	Yes	Yes	Yes	Yes	No	No	Bangla	No	Young (15-29 yrs)	became preg <19	No	4000-9000	Perceived appro. time	Involve in income generation	2nd trimesters	No	Private clinic/doctor	daily lab/f	>10 km	Didn't attend - primary	Didn't attend - Primary	No	Yes	No	No	1-2 pregnancies	didn't-junior school	didn't-junior school	Do not know	No	Mild	Health complex & providers	Daily labor/Farmer	<4 ANC	Did not pay
370	41	Matiranga	16-24	Chakma	Buddhist	No	No	Yes	Yes	Don't kno	No					Young (15-29 yrs)	became preg <19	Yes	10000-19000		Involve in income generation		No		Service hc	6-10km	Didn't attend - primary	Didn't attend - Primary	No	No	Yes	No	1-2 pregnancies	didn't-junior school	didn't-junior school	Secondary hospitals	Yes	Severe (need medical attention)	Relatives & other	Employed/Business	Did not access	
371	41	Matiranga	16-24	Chakma	Buddhist	No	No	Yes	No							Young (15-29 yrs)	became preg <19	Yes	4000-9000		Complete housewife		No		Service hc	6-10km	Didn't attend - primary	Didn't attend - Primary	No	No	No	No	1-2 pregnancies	didn't-junior school	didn't-junior school	Secondary hospitals	Yes	Mild		Employed/Business	Did not access	
372	41	Matiranga	16-24	Chakma	Buddhist	No	No	No	No							Young (15-29 yrs)	became preg <19	No	4000-9000		Involve in income generation		No		daily lab/f	6-10km	Didn't attend - primary	Secondary school	No	No	No	No	1-2 pregnancies	didn't-junior school	didn't-junior school	Do not know	No	No problem reported		Daily labor/Farmer	Did not access	
373	41	Matiranga	25-29	Chakma	Buddhist	Yes	No	Yes	Yes	Yes	Yes	No	No	Bangla	No	Young (15-29 yrs)	became preg <19	Yes	4000-9000	HW came home	Involve in income generation	2nd trimesters	No	Community health w	Service hc	6-10km	Didn't attend - primary	Didn't attend - Primary	No	Yes	No	No	3 & above pregnancies	didn't-junior school	didn't-junior school	Secondary hospitals	Yes	Mild	Health complex & providers	Employed/Business	=>4 ANC	Did not pay
374	41	Matiranga	16-24	Chakma	Buddhist	No	No	No	Yes	Don't kno	Yes	No	No	Bangla	No	Young (15-29 yrs)	became preg <19	No	20000 and above	HW came home	Involve in income generation	2nd trimesters	No	Private clinic/doctor	Service hc	6-10km	Didn't attend - primary	Secondary school	No	Yes	No	No	1-2 pregnancies	didn't-junior school	didn't-junior school	Do not know	No	No problem reported	Health complex & providers	Employed/Business	<4 ANC	Did not pay
375	41	Matiranga	=>30	Chakma	Buddhist	No	Yes	Yes	Yes	Yes	Yes	Yes	No	Bangla	No	Adult (30 & above v	became pregnant 20+ yrs	No	20000 and above	HW came home	Complete housewife	2nd trimesters	No	Community health w	Service hc	6-10km	Secondary school	Higher secondary & above	Yes	Yes	No	No	3 & above pregnancies	didn't-junior school	secondary & above	Do not know	No	Severe (need medical attention)	Relatives & other	Employed/Business	<4 ANC	<=300 tk
376	41	Matiranga	=>30	Chakma	Buddhist	Yes	Yes	Yes	Yes	Yes	Yes	No	No	Bangla	Yes	Adult (30 & above v	became pregnant 20+ yrs	Yes	10000-19000	HW came home	Involve in income generation	2nd trimesters	No	UHC or other priman	Service hc	6-10km	Secondary school	Secondary school	No	Yes	No	No	3 & above pregnancies	Secondary & above	secondary & above	Community_based centres	Yes	Mild	Health complex & providers	Employed/Business	=>4 ANC	Did not pay
377	41	Matiranga	25-29	Chakma	Buddhist	No	Yes	Yes	No							Young (15-29 yrs)	became preg <19	Yes	20000 and above		Involve in income generation		No		Service hc	6-10km	Secondary school	Secondary school	No	No	No	No	3 & above pregnancies	didn't-junior school	secondary & above	Secondary hospitals	Yes	Mild		Employed/Business	Did not access	
378	41	Matiranga	=>30	Chakma	Buddhist	No	No	Yes	No							Adult (30 & above v	became pregnant 20+ yrs	No	4000-9000		Involve in income generation		No		daily lab/f	6-10km	Didn't attend - primary	Didn't attend - Primary	No	No	No	No	1-2 pregnancies	didn't-junior school	didn't-junior school	Do not know	No	Moderate (debiliated daily life)		Daily labor/Farmer	Did not access	
379	42	Matiranga	=>30	Chakma	Buddhist	No	No	Yes	Yes	Don't kno	No					Adult (30 & above v	became preg <19	Yes	20000 and above		Involve in income generation		No		Service hc	6-10km	Didn't attend - primary	Secondary school	Yes	Yes	Yes	No	3 & above pregnancies	didn't-junior school	didn't-junior school	Secondary hospitals	Yes	Severe (need medical attention)	Relatives & other	Employed/Business	Did not access	
380	42	Matiranga	16-24	Chakma	Buddhist	No	No	Yes	No							Young (15-29 yrs)	became preg <19	Yes	4000-9000		Involve in income generation		No		daily lab/f	6-10km	Didn't attend - primary	Secondary school	No	No	No	No	1-2 pregnancies	didn't-junior school	didn't-junior school	Secondary hospitals	Yes	Severe (need medical attention)		Daily labor/Farmer	Did not access	
381	42	Matiranga	25-29	Chakma	Buddhist	No	No	No	No							Young (15-29 yrs)	became preg <19	No	4000-9000		Complete housewife		No		daily lab/f	6-10km	Didn't attend - primary	Didn't attend - Primary	No	No	No	No	3 & above pregnancies	didn't-junior school	didn't-junior school	Do not know	No	No problem reported		Daily labor/Farmer	Did not access	
382	42	Matiranga	=>30	Chakma	Buddhist	Yes	No	No	No							Adult (30 & above v	became preg <19	No	4000-9000		Involve in income generation		No		daily lab/f	6-10km	Didn't attend - primary	Didn't attend - Primary	No	No	No	No	3 & above pregnancies	didn't-junior school	didn't-junior school	Do not know	No	No problem reported		Daily labor/Farmer	Did not access	
383	42	Matiranga	25-29	Chakma	Buddhist	Yes	No	No	No							Young (15-29 yrs)	became preg <19	Yes	4000-9000		Involve in income generation		No		Service hc	6-10km	Didn't attend - primary	Didn't attend - Primary	No	No	No	No	3 & above pregnancies	didn't-junior school	didn't-junior school	Secondary hospitals	Yes	No problem reported		Employed/Business	Did not access	
384	43	Matiranga	16-24	Chakma	Buddhist	No	No	Yes	Yes	Yes	No					Young (15-29 yrs)	became preg <19	Yes	4000-9000		Involve in income generation		No		Service hc	6-10km	Secondary school	Secondary school	No	No	Yes	No	1-2 pregnancies	Secondary & above	didn't-junior school	Secondary hospitals	Yes	Mild	Relatives & other	Employed/Business	Did not access	
385	43	Matiranga	16-24	Chakma	Buddhist	No	No	Yes	Yes	Yes	No					Young (15-29 yrs)	became pregnant 20+ yrs	Yes	20000 and above		Complete housewife		No		Service hc	6-10km	Secondary school	Higher secondary & above	No	Yes	No	No	1-2 pregnancies	Secondary & above	secondary & above	Private clinic	Yes	Moderate (debiliated daily life)	Health complex & providers	Employed/Business	Did not access	
386	43	Matiranga	25-29	Chakma	Buddhist	No	No	Yes	No							Young (15-29 yrs)	became pregnant 20+ yrs	No	10000-19000		Involve in income generation		No		Service hc	6-10km	Didn't attend - primary	Didn't attend - Primary	No	No	No	No	1-2 pregnancies	didn't-junior school	didn't-junior school	Do not know	No	Mild		Employed/Business	Did not access	
387	43	Matiranga	25-29	Chakma	Buddhist	Yes	No	Yes	Yes	Yes	Yes	Yes	No	Bangla	No	Adult (30 & above v	became preg <19	Yes	10000-19000	Foetus sex	Complete housewife	3rd trimesters	No	UHC or other priman	daily lab/f	6-10km	Didn't attend - primary	Secondary school	No	Yes	No	No	3 & above pregnancies	didn't-junior school	didn't-junior school	Community_based centres	Yes	Mild	Health complex & providers	Daily labor/Farmer	<4 ANC	>300 tk
388	43	Matiranga	16-24	Chakma	Buddhist	No	No	Yes	No							Young (15-29 yrs)	became preg <19	Yes	4000-9000		Complete housewife		No		daily lab/f	6-10km	Secondary school	Didn't attend - Primary	No	No	No	No	1-2 pregnancies	Secondary & above	didn't-junior school	Secondary hospitals	Yes	Severe (need medical attention)		Daily labor/Farmer	Did not access	
389	43	Matiranga	16-24	Chakma	Buddhist	No	No	Yes	No							Young (15-29 yrs)	became preg <19	No	4000-9000		Involve in income generation		No		daily lab/f	6-10km	Secondary school	Didn't attend - Primary	No	No	No	No	1-2 pregnancies	didn't-junior school	didn't-junior school	Do not know	No	Severe (need medical attention)		Daily labor/Farmer	Did not access	
390	43	Matiranga	16-24	Chakma	Buddhist	No	No	Yes	No							Young (15-29 yrs)	became preg <19	Yes	4000-9000		Involve in income generation		No		daily lab/f	6-10km	Secondary school	Didn't attend - Primary	No	No	No	No	1-2 pregnancies	didn't-junior school	didn't-junior school	Community_based centres	Yes	Mild		Daily labor/Farmer	Did not access	
391	44	Matiranga	25-29	Chakma	Buddhist	No	Yes	Yes	Yes	Yes	Yes	No	No	Bangla	Yes	Young (15-29 yrs)	became pregnant 20+ yrs	Yes	20000 and above	Confirm pregnancy	Complete housewife	1st trimesters	No	Private clinic/doctor	daily lab/f	>10 km	Higher secondary & above	Secondary school	No	No	Yes	Yes	1-2 pregnancies	Secondary & above	secondary & above	Private clinic	Yes	Severe (need medical attention)	Relatives & other	Daily labor/Farmer	=>4 ANC	Did not pay
392	44	Matiranga	25-29	Chakma	Buddhist	No	No	Yes	Yes	Yes	Yes	Yes	No	Bangla	No	Young (15-29 yrs)	became pregnant 20+ yrs	Yes	10000-19000	Confirm pregnancy	Involve in income generation	1st trimesters	No	Private clinic/doctor	Service hc	>10 km	Didn't attend - primary	Secondary school	No	No	Yes	No	1-2 pregnancies	didn't-junior school	secondary & above	Private clinic	Yes	Severe (need medical attention)	Relatives & other	Employed/Business	=>4 ANC	<=300 tk
393	44	Matiranga	16-24	Chakma	Buddhist	No	No	Yes	No							Young (15-29 yrs)	became preg <19	No	10000-19000		Involve in income generation		No		Business	>10 km	Didn't attend - primary	Secondary school	No	No	No	No	1-2 pregnancies	didn't-junior school	secondary & above	Do not know	No	Mild		Employed/Business	Did not access	
394	44	Matiranga	16-24	Chakma	Buddhist	No	No	Yes	No							Young (15-29 yrs)	became preg <19	No	4000-9000		Involve in income generation		No		daily lab/f	>10 km	Didn't attend - primary	Didn't attend - Primary	No	No	No	No	1-2 pregnancies	didn't-junior school	didn't-junior school	Do not know	No	Severe (need medical attention)		Daily labor/Farmer	Did not access	
395	44	Matiranga	25-29	Chakma	Buddhist	No	Yes	Yes	Yes	Yes	Yes	No	No	Bangla	No	Young (15-29 yrs)	became pregnant 20+ yrs	Yes	20000 and above	Perceived appro. time	Complete housewife	2nd trimesters	No	Govt Health Comple	daily lab/f	>10 km	Secondary school	Secondary school	Yes	Yes	No	No	1-2 pregnancies	Secondary & above	secondary & above	Private clinic	Yes	Severe (need medical attention)	Health complex & providers	Daily labor/Farmer	<4 ANC	Did not pay
396	44	Matiranga	16-24	Chakma	Buddhist	No	No	No	Yes	Yes	Yes	Yes	Yes	Bangla	Yes	Young (15-29 yrs)	became pregnant 20+ yrs	Yes	20000 and above	Know foetus condition	Complete housewife	2nd trimesters	No	UHC or other priman	Service hc	>10 km	Secondary school	Secondary school	No	Yes	No	No	1-2 pregnancies	didn't-junior school	secondary & above	Community_based centres	Yes	No problem reported	Health complex & providers	Employed/Business	<4 ANC	>300 tk
397	44	Matiranga	25-29	Chakma	Buddhist	Yes	No	No	No							Young (15-29 yrs)	became preg <19	No	20000 and above		Complete housewife		No		Service hc	>10 km	Secondary school	Secondary school	No	No	No	No	3 & above pregnancies	didn't-junior school	secondary & above	Do not know	No	No problem reported		Employed/Business	Did not access	
398	45	Matiranga	25-29	Chakma	Buddhist	No	No	Yes	No							Young (15-29 yrs)	became preg <19	Yes	4000-9000		Involve in income generation		No		daily lab/f	>10 km	Didn't attend - primary	Didn't attend - Primary	No	No	No	No	1-2 pregnancies	didn't-junior school	didn't-junior school	Community_based centres	Yes	Severe (need medical attention)		Daily labor/Farmer	Did not access	
399	45	Matiranga	25-29	Chakma	Buddhist	No	No	Yes	Yes	Yes	Yes	No	No	Bangla	No	Young (15-29 yrs)	became preg <19	Yes	4000-9000	Foetus sex	Involve in income generation	2nd trimesters	No	Private clinic/doctor	daily lab/f	>10 km	Didn't attend - primary	Didn't attend - Primary	No	No	Yes	No	1-2 pregnancies	didn't-junior school	didn't-junior school	Private clinic	Yes	Severe (need medical attention)	Relatives & other	Daily labor/Farmer	<4 ANC	Did not pay
400	45	Matiranga	25-29	Chakma	Buddhist	No	No	No	Yes	Yes	No					Young (15-29 yrs)	became pregnant 20+ yrs	Yes	4000-9000		Involve in income generation		No		Service hc	>10 km	Secondary school	Didn't attend - Primary	No	Yes	No	No	1-2 pregnancies	didn't-junior school	didn't-junior school	Private clinic	Yes	No problem reported	Health complex & providers	Employed/Business	Did not access	
401	45	Matiranga	25-29	Chakma	Buddhist	No	No	Yes	Yes	Don't kno	No					Young (15-29 yrs)	became preg <19	No	4000-9000		Involve in income generation		No		Service hc	>10 km	Didn't attend - primary	Didn't attend - Primary	No	No	Yes	No	1-2 pregnancies	didn't-junior school	didn't-junior school	Do not know	No	Mild	Relatives & other	Employed/Business	Did not access	
402	45	Matiranga	16-24	Chakma	Buddhist	No	No	Yes	No							Young (15-29 yrs)	became preg <19	Yes	4000-9000		Involve in income generation		No		daily lab/f	>10 km	Didn't attend - primary	Didn't attend - Primary	No	No	No	No	1-2 pregnancies	didn't-junior school	didn't-junior school	Community_based centres	Yes	Mild		Daily labor/Farmer	Did not access	
403	45	Matiranga	16-24	Chakma	Buddhist	No	No	Yes	No							Young (15-29 yrs)	became preg <19	No	4000-9000		Involve in income generation		No		daily lab/f	>10 km	Didn't attend - primary	Didn't attend - Primary	No	No	No	No	1-2 pregnancies	didn't-junior school	didn't-junior school	Do not know	No	Severe (need medical attention)		Daily labor/Farmer	Did not access	
404	45	Matiranga	25-29	Chakma	Buddhist	Yes	No	Yes	Yes	Yes	No					Young (15-29 yrs)	became preg <19	Yes	4000-9000		Involve in income generation		No		daily lab/f	>10 km	Didn't attend - primary	Didn't attend - Primary	No	No	Yes	No	3 & above pregnancies	didn't-junior school	didn't-junior school	Community_based centres	Yes	Mild	Relatives & other	Daily labor/Farmer	Did not access	
405	46	Matiranga	=>30	Chakma	Buddhist	No	Yes	Yes	Yes	Yes	Yes	No	No	Bangla	No	Adult (30 & above v	became preg <19	Yes	20000 and above	Know foetus condition	Involve in income generation	3rd trimesters	No	UHC or other priman	Business	>10 km	Secondary school	Secondary school	No	No	Yes	No	1-2 pregnancies	didn't-junior school	didn't-junior school	Secondary hospitals	Yes	Severe (need medical attention)	Relatives & other	Employed/Business	<4 ANC	Did not pay
406	46	Matiranga	16-24	Chakma	Buddhist	No	No	No	Yes	Don't kno	Yes	Yes	No	Bangla	No	Young (15-29 yrs)	became preg <19	Yes	20000 and above	Foetus sex	Involve in income generation	3rd trimesters	No	Private clinic/doctor	Service hc	>10 km	Secondary school	Secondary school	No	Yes	Yes	No	1-2 pregnancies	Secondary & above	didn't-junior school	Private clinic	Yes	No problem reported	Relatives & other	Employed/Business	<4 ANC	>300 tk
407	46	Matiranga	25-29	Chakma	Buddhist	Yes	No	Yes	No							Young (15-29 yrs)	became preg <19	Yes	10000-19000		Involve in income generation		No		Business	>10 km	Didn't attend - primary	Secondary school	No	No	No	No	3 & above pregnancies	didn't-junior school	didn't-junior school	Secondary hospitals	Yes	Severe (need medical attention)		Employed/Business	Did not access	
408	46	Matiranga	=>30	Chakma	Buddhist	No	No	Yes	Yes	Yes	No					Adult (30 & above v	became preg <19	Yes	10000-19000		Involve in income generation		No		Service hc	>10 km	Secondary school	Secondary school	No	No	Yes	No	1-2 pregnancies	didn't-junior school	didn't-junior school	Secondary hospitals	Yes	Mild	Relatives & other	Employed/Business	Did not access	
409	46	Matiranga	25-29	Chakma	Buddhist	Yes	No	Yes	Yes	Yes	Yes	No	No	Bangla	No	Young (15-29 yrs)	became preg <19	Yes	10000-19000	Illness/complications	Involve in income generation	2nd trimesters	No	Community health w	Service hc	>10 km	Didn't attend - primary	Didn't attend - Primary	No	Yes	No	No	3 & above pregnancies	didn't-junior school	didn't-junior school	Secondary hospitals	Yes	Mild	Health complex & providers	Employed/Business	<4 ANC	Did not pay
410	46	Matiranga	16-24	Chakma	Buddhist	No	No	Yes	No							Young (15-29 yrs)	became preg <19	No	10000-19000		Involve in income generation		No		Service hc	>10 km	Secondary school	Secondary school	No	No	No	No	1-2 pregnancies	Secondary & above	didn't-junior school	Do not know	No	Moderate (debiliated daily life)		Employed/Business	Did not access	
411	46	Matiranga	=>30	Chakma	Buddhist	No	No	Yes	Yes	Don't kno	Yes	No	No	Bangla	No	Adult (30 & above v	became preg <19	No	4000-9000	Perceived appro. time	Involve in income generation	2nd trimesters	No	Private clinic/doctor	daily lab/f	>10 km	Didn't attend - primary	Didn't attend - Primary	No	Yes	No	No	1-2 pregnancies	didn't-junior school	didn't-junior school	Do not know	No	Mild	Health complex & providers	Daily labor/Farmer	=>4 ANC	Did not pay
412	46	Matiranga	=>30	Chakma	Buddhist	No	No	Yes	Yes	Yes	Yes	No	Yes	Your own language		Adult (30 & above v	became pregnant 20+ yrs	Yes	20000 and above	Illness/complications	Complete housewife	2nd trimesters	Yes	Community health w	Service hc	>10 km	Higher secondary & above	Secondary school	No	Yes	Yes	No	1-2 pregnancies	Secondary & above	secondary & above	Private clinic	Yes	Moderate (debiliated daily life)	Relatives & other	Employed/Business	=>4 ANC	Did not pay
413	46	Matiranga	=>30	Chakma	Buddhist	Yes	No	Yes	Yes	Yes	Yes	No	No	Bangla	No	Adult (30 & above v	became preg <19	No	10000-19000	Perceived appro. time	Involve in income generation	2nd trimesters	No	Private clinic/doctor	daily lab/f	>10 km	Didn't attend - primary	Didn't attend - Primary	No	Yes	No	No	3 & above pregnancies	didn't-junior school	didn't-junior school	Do not know	No	Mild	Health complex & providers	Daily labor/Farmer	<4 ANC	Did not pay
414	47	Matiranga	=>30	Chakma	Buddhist	No	No	Yes	Yes	Yes	Yes	No	No	Bangla	No	Adult (30 & above v	became preg <19	Yes	4000-9000	other	Involve in income generation	1st trimesters	No	Private clinic/doctor	daily lab/f	>10 km	Didn't attend - primary	Didn't attend - Primary	No	Yes	Yes	No	1-2 pregnancies	didn't-junior school	didn't-junior school	Secondary hospitals	Yes	Severe (need medical attention)	Relatives & other	Daily labor/Farmer	=>4 ANC	Did not pay
415	44	Matiranga	16-24	Chakma	Buddhist	No	No	Yes	No							Young (15-29 yrs)	became preg <19	No	4000-9000		Involve in income generation		No		daily lab/f	>10 km	Didn't attend - primary	Didn't attend - Primary	No	No	No	No	1-2 pregnancies	didn't-junior school	didn't-junior school	Do not know	No	Mild		Daily labor/Farmer	Did not access	
416	44	Matiranga	16-24	Chakma	Buddhist	Yes	No	Yes	No							Young (15-29 yrs)	became preg <19	No	4000-9000		Involve in income generation		No		daily lab/f	>10 km	Didn't attend - primary	Didn't attend - Primary	No	No	No	No	1-2 pregnancies	didn't-junior school	didn't-junior school	Do not know	No	Severe (need medical attention)		Daily labor/Farmer	Did not access	
417	44	Matiranga	25-29	Chakma	Buddhist	Yes	No	Yes	Yes	Yes	Yes	Yes	No	Bangla	No	Young (15-29 yrs)	became preg <19	Yes	10000-19000	Foetus sex	Complete housewife	2nd trimesters	No	Private clinic/doctor	Service hc	>10 km	Didn't attend - primary	Secondary school	Yes	Yes	Yes	No	3 & above pregnancies	didn't-junior school	didn't-junior school	Community_based centres	Yes	Severe (need medical attention)	Relatives & other	Employed/Business	<4 ANC	>300 tk
418	29	Matiranga	16-24	Chakma	Buddhist	No	No	Yes	Yes	Yes	No					Young (15-29 yrs)	became preg <19	Yes	10000-19000		Involve in income generation		No		Service hc	6-10km	Secondary school	Didn't attend - Primary	No	Yes	Yes	No	1-2 pregnancies	didn't-junior school	didn't-junior school	Secondary hospitals	Yes	Mild	Relatives & other	Employed/Business	Did not access	
419	47	Matiranga	25-29	Chakma	Buddhist	No	No	No	Yes	Yes	No					Young (15-29 yrs)	became pregnant 20+ yrs	No	20000 and above		Complete housewife		No		Service hc	>10 km	Secondary school	Secondary school	Yes	Yes	No	No	1-2 pregnancies	Secondary & above	secondary & above	Do not know	No	No problem reported	Health complex & providers	Employed/Business	Did not access	
420	47	Matiranga	=>30	Chakma	Buddhist	No	No	Yes	Yes	Yes	No					Adult (30 & above v	became preg <19	No	10000-19000		Involve in income generation		No		Service hc	>10 km	Secondary school	Didn't attend - Primary	No	No	Yes	No	3 & above pregnancies	didn't-junior school	didn't-junior school	Do not know	No	Severe (need medical attention)	Relatives & other	Employed/Business	Did not access	
421	47	Matiranga	25-29	Chakma	Buddhist	Yes	Yes	Yes	Yes	Yes	No					Young (15-29 yrs)	became preg <19	No	4000-9000		Involve in income generation		No		daily lab/f	>10 km	Didn't attend - primary	Didn't attend - Primary	No	Yes	Yes	No	3 & above pregnancies	didn't-junior school	didn't-junior school	Do not know	No	Severe (need medical attention)	Relatives & other	Daily labor/Farmer	Did not access	
422	46	Matiranga	25-29	Chakma	Buddhist	Yes	No	Yes	Yes	Yes	No					Young (15-29 yrs)	became preg <19	Yes	10000-19000		Involve in income generation		No		Service hc	>10 km	Secondary school	Secondary school	Yes	Yes	Yes	No	3 & above pregnancies	didn't-junior school	didn't-junior school	Secondary hospitals	Yes	Severe (need medical attention)	Relatives & other	Employed/Business	Did not access	
423	46	Matiranga	25-29	Chakma	Buddhist	Yes	Yes	Yes	No							Young (15-29 yrs)	became preg <19	Yes	4000-9000		Involve in income generation		No		daily lab/f	>10 km	Didn't attend - primary	Secondary school	No	No	No	No	3 & above pregnancies	didn't-junior school	didn't-junior school	Secondary hospitals	Yes	Severe (need medical attention)		Daily labor/Farmer	Did not access	
424	34	Matiranga	25-29	Chakma	Buddhist	Yes	No	Yes	No							Young (15-29 yrs)	became preg <19	Yes	10000-19000		Involve in income generation		No		Service hc	>10 km	Secondary school	Didn't attend - Primary	No	No	No	No	3 & above pregnancies	didn't-junior school	didn't-junior school	Community_based centres	Yes	Mild		Employed/Business	Did not access	
425	34	Matiranga	16-24	Chakma	Buddhist	Yes	No	Yes	No							Young (15-29 yrs)	became preg <19	No	10000-19000		Complete housewife		No		Service hc	>10 km	Secondary school	Secondary school	No	No	No	No	1-2 pregnancies	didn't-junior school	secondary & above	Do not know	No	Mild		Employed/Business	Did not access	
426	34	Matiranga	16-24	Chakma	Buddhist	Yes	No	Yes	No							Young (15-29 yrs)	became preg <19	Yes	4000-9000		Involve in income generation		No		daily lab/f	>10 km	Didn't attend - primary	Didn't attend - Primary	No	No	No	No	1-2 pregnancies	didn't-junior school	didn't-junior school	Secondary hospitals	Yes	Severe (need medical attention)		Daily labor/Farmer	Did not access	
427	29	Matiranga	=>30	Chakma	Buddhist	Yes	Yes	Yes	Yes	Yes	No					Adult (30 & above v	became preg <19	Yes	4000-9000		Involve in income generation		No		daily lab/f	6-10km	Secondary school	Didn't attend - Primary	No	Yes	Yes	No	3 & above pregnancies	didn't-junior school	didn't-junior school	Community_based centres	Yes	Severe (need medical attention)	Relatives & other	Daily labor/Farmer	Did not access	
428	17	Matiranga	25-29	Tripura	Sonatoni	No	No	Yes	Yes	Yes	Yes	No	No	Bangla	No	Young (15-29 yrs)	became preg <19	Yes	20000 and above	Illness/complications	Complete housewife	2nd trimesters	Yes	Private clinic/doctor	Service hc	6-10km	Secondary school	Secondary school	No	No	Yes	No	1-2 pregnancies	Secondary & above	secondary & above	Private clinic	Yes	Mild	Relatives & other	Employed/Business	=>4 ANC	Did not pay
429	2	Matiranga	16-24	Tripura	Sonatoni	Yes	No	Yes	Yes	Yes	Yes	No	No	Bangla	No	Young (15-29 yrs)	became preg <19	Yes	4000-9000	Confirm pregnancy	Involve in income generation	1st trimesters	No	Home visit by HW/w	daily lab/f	6-10km	Didn't attend - primary	Didn't attend - Primary	No	Yes	Yes	No	1-2 pregnancies	didn't-junior school	didn't-junior school	Community_based centres	Yes	Severe (need medical attention)	Relatives & other	Daily labor/Farmer	=>4 ANC	Did not pay
430	2	Matiranga	25-29	Tripura	Sonatoni	Yes	No	Yes	Yes	Yes	Yes	No	No	Bangla	No	Young (15-29 yrs)	became preg <19	No	4000-9000	Illness/complications	Involve in income generation	2nd trimesters	No	Community health w	daily lab/f	6-10km	Didn't attend - primary	Didn't attend - Primary	No	Yes	No	No	3 & above pregnancies	didn't-junior school	didn't-junior school	Do not know	No	Severe (need medical attention)	Health complex & providers	Daily labor/Farmer	<4 ANC	Did not pay
431	5	Matiranga	16-24	Tripura	Sonatoni	Yes	Yes	Yes	Yes	Yes	Yes	Yes	No	Bangla	No	Adult (30 & above v	became preg <19	Yes	4000-9000	Foetus sex	Involve in income generation	2nd trimesters	No	Private clinic/doctor	daily lab/f	>10 km	Didn't attend - primary	Didn't attend - Primary	No	Yes	No	No	3 & above pregnancies	didn't-junior school	didn't-junior school	Community_based centres	Yes	Severe (need medical attention)	Health complex & providers	Daily labor/Farmer	=>4 ANC	>300 tk
432	20	Matiranga	16-24	Tripura	Sonatoni	No	Yes	Yes	Yes	Yes	No					Young (15-29 yrs)	became preg <19	Yes	4000-9000		Involve in income generation		No		daily lab/f	6-10km	Secondary school	Didn't attend - Primary	No	Yes	Yes	No	1-2 pregnancies	didn't-junior school	didn't-junior school	Community_based centres	Yes	Mild	Relatives & other	Daily labor/Farmer	Did not access	
433	3	Matiranga	16-24	Tripura	Sonatoni	Yes	No	Yes	No							Young (15-29 yrs)	became preg <19	Yes	4000-9000		Involve in income generation		No		daily lab/f	>10 km	Didn't attend - primary	Didn't attend - Primary	No	No	No	No	3 & above pregnancies	didn't-junior school	didn't-junior school	Community_based centres	Yes	Severe (need medical attention)		Daily labor/Farmer	Did not access	
434	5	Matiranga	25-29	Tripura	Sonatoni	Yes	No	Yes	Yes	Yes	No					Young (15-29 yrs)	became preg <19	Yes	4000-9000		Involve in income generation		No		daily lab/f	>10 km	Didn't attend - primary	Didn't attend - Primary	No	Yes	No	No	3 & above pregnancies	didn't-junior school	didn't-junior school	Community_based centres	Yes	Mild	Health complex & providers	Daily labor/Farmer	Did not access	
435	5	Matiranga	25-29	Tripura	Sonatoni	Yes	No	Yes	No							Young (15-29 yrs)	became pregnant 20+ yrs	Yes	4000-9000		Involve in income generation		No		daily lab/f	>10 km	Didn't attend - primary	Didn't attend - Primary	No	No	No	No	3 & above pregnancies	didn't-junior school	didn't-junior school	Community_based centres	Yes	Moderate (debiliated daily life)		Daily labor/Farmer	Did not access	
436	5	Matiranga	25-29	Tripura	Sonatoni	Yes	No	Yes	Yes	Yes	Yes	No	No	Bangla	No	Young (15-29 yrs)	became preg <19	Yes	4000-9000	Illness/complications	Involve in income generation	3rd trimesters	No	Community health w	daily lab/f	>10 km	Secondary school	Didn't attend - Primary	No	Yes	No	No	3 & above pregnancies	didn't-junior school	didn't-junior school	Community_based centres	Yes	Mild	Health complex & providers	Daily labor/Farmer	<4 ANC	Did not pay
437	20	Matiranga	16-24	Tripura	Sonatoni	Yes	No	Yes	Yes	Yes	Yes	No	No	Bangla	Yes	Young (15-29 yrs)	became preg <19	Yes	4000-9000	Illness/complications	Involve in income generation	3rd trimesters	No	Private clinic/doctor	daily lab/f	6-10km	Secondary school	Didn't attend - Primary	No	Yes	Yes	No	3 & above pregnancies	didn't-junior school	didn't-junior school	Secondary hospitals	Yes	Severe (need medical attention)	Relatives & other	Daily labor/Farmer	<4 ANC	Did not pay
438	20	Matiranga	=>30	Tripura	Sonatoni	No	No	Yes	Yes	Yes	Yes	No	No	Bangla	No	Adult (30 & above v	became preg <19	Yes	4000-9000	Perceived appro. time	Involve in income generation	2nd trimesters	No	Private clinic/doctor	Service hc	6-10km	Secondary school	Didn't attend - Primary	No	Yes	Yes	No	3 & above pregnancies	didn't-junior school	didn't-junior school	Community_based centres	Yes	Severe (need medical attention)	Relatives & other	Employed/Business	<4 ANC	Did not pay
