# Supplementary material for: Prevalence and factors associated with antenatal care service access among Indigenous women in the Chittagong Hill Tracts, Bangladesh: A cross-sectional study
Source: PLoS One. 2020 Dec 29;15(12):e0244640. doi: 10.1371/journal.pone.0244640 (PMC7771700; doi:10.1371/journal.pone.0244640)
Supplement: S2 Table — (PDF) [file pone.0244640.s002.pdf]

Supplementary table 2: Variable dictionary for “**Prevalence and factors associated with antenatal care service access among Indigenous women in the Chittagong Hill Tracts, Bangladesh: A cross-sectional study” (Akter et al.)**

Variable name	Description
record_id	Unique ID for each participants
village_clus	Villages in Matiranga and Khagrachhari Sadar subdistrict
Subdistric	Subdistrict of Khagrachaari hill district
ethnic_identity	Ethnicity of the participant
religion	Religion
age_cat_new	Participants age (in years) during survey
pregnancy_outcome	any pregnancy outcome resulted in live/ stillbirth/ abortion/ miscarriage
health_problem_pregnancy	Knew about health problems related to pregnancy
problem_lastpregnancy	If the participant had any health related problems during her last pregnancy
anc_knowledge	If the participant had knowledge about ANC.
anc_benefit	If the participant had knowledge about ANC. benefit
last_anc_attndnc	If the participant attended ANC visit during last pregnancy
payment_for_anc	If the participant had to pay for ANC visit
anc_info_preg_cmplctn	If the participant was told about the signs of pregnancy complications during ANC check
language_mode_anc	Mode of language during ANC consultation
bangla_anc_info	As the providers used Bangla language, did the participant understand the information given?
age_categorise	Participants’ Age in categories (years)
age_pregnancy_new	age at first pregnancy (below 20 and above 20 yrs old)
know_anyNearest_facility	If participant knew any nearest facility
HH_income_Status	Monthly household income in Bangladeshi taka
ANC_knowledge_info	Source of information for ANC check
last_anc_visit_decision_full	Reasons for ANC check during last pregnancy
new_occupation_participant	Occupation of the participants
trimesters_anc_check_last	Which trimester of pregnancy the participant visited ANC for her last ANC check
any_media_forMHS	If the participant access any media for maternal health care information
anc_service_use_new	Facility where the participant access for ANC check during last pregnancy
partner_occup_new_3cat	Partner’s occupation
distance_known_facility_cat	Distance to facility from participants residence
school_attend_new3cat	Participants’ school attendance
school_attnd_partner_new3cat	Partner of the participant’s school attendance
anc_info_source_healthComplx	Health complex as a source of info about ANC from
anc_info_source_HCP	Health care providers as a source of info about ANC from
anc_info_source_F&F&Nei	Family, friend and neighbours as a source of info about ANC from

anc_info_source_other	“Other” as a source of info about ANC from
total_preg_2cat	Total number of pregnancies the participant had till survey date
school_attend_new2cat	participant's school attendance
school_attend_partner_new2cat	School attendance of partners into 2 categories
know_nearest_facility_merged4cat	Participant’s knowledge about nearest facilities in 3 cat
know_anyNearest_facility	If the participant knew the nearest health facilities to her home
last_preg_prob_severity	Severity of pregnancy problems that participants had in her last pregnancy
ANC_source_info	Source of information for ANC check
occupation_partner_2cat	Partner’s occupation in two categories
ANC_4visit_3cat	Number of ANC visit (based on 4-ANC visits)
anc_pay_3catNew	Amount of money the participant pay for ANC check
