# Supplementary material for: Prevalence and factors associated with antenatal care service access among Indigenous women in the Chittagong Hill Tracts, Bangladesh: A cross-sectional study
Source: PLoS One. 2020 Dec 29;15(12):e0244640. doi: 10.1371/journal.pone.0244640 (PMC7771700; doi:10.1371/journal.pone.0244640)
Supplement: Attachment — Submitted filename: Response to Reviewer.docx [file pone.0244640.s003.docx]

**Akter et al.: Prevalence and factors associated with antenatal care service access among Indigenous women in the Chittagong Hill Tracts, Bangladesh: A cross-sectional study**

The authors thank the Editor and Reviewers for their valuable comments to improve the quality of this manuscript. We have addressed the recommended revisions in the manuscript using track changes and provide a detailed response to each comment below.

| **Additional editor#1** | **Authors’ response** |
| --- | --- |
| Please address comments from the reviewer and those following. Would advise you consult the Strobe checklist in shaping up your manuscript:  Rephrase line 61, it is not that clear. Also be consistent in your use of percentages or absolute numbers when presenting the results. | Thank you the STROBE checklist for cross sectional studies has now been used to guide the reporting in this manuscript.  Line 61 has been revised: [*Of 494 Indigenous women in two subdistricts, 438 participated (89% response rate) in the study, 75% of these women were aged 15-29 years.]* [Please, see line 61-62 in the revised document]  The reporting of percentages and absolute numbers have been revised and standardised and reported consistently throughout the manuscript. |
| Lines 96, 116, 162, etc.: New sentences are usually not started with abbreviations like ANC- you start them with the full expression | Thank you, this has been addressed throughout the manuscript. |
| Line 96 - 98: The sentence is not clear – revise | This sentence has been revised: *Antenatal care provides opportunities to educate women about reproductive health including about other MHC services including safe delivery and child vaccination*. [please, see line 98-100 in the revised document] |
| Line 114: Last sentence needs revision - possibly "after twenty years of signing the peace accord" | This has been revised as suggested. [Please, see line 116 in the revised document] |
| Lines 130 - 131: The part of the sentence beyond the "comma" is not clear - revise | This sentence has been revised:  *The percentage of low-birth-weight newborn babies was found highest in the CHT Indigenous women compared to non-Indigenous* women *in a report published by UNICEF 2015 (United Nations Children’s Funds)* [please, see line 132-138] |
| Lines 136 - 137: The expression "the top down nature of health intervention programs" could mean different things to different readers, so please be more specific | This sentence has been revised and the term top-down removed: *A recent systematic review indicated that health intervention programs for Indigenous women in LMIC where community-engagement was not prioritised resulted in culturally unfriendly MHC services^[1]^. This may impact access to ANC*. [please, see line 143-146 in the revised document] |
| Lines 146 - 149: Under "methods", provide information on the study setting including all the communities you mention in the results section that were not earlier mentioned - that could include info on health facilities, etc. Also, how did you arrive at the sample size??? Could you provide reasons for participants having to be "within 36 months of delivery"? | Thank you the methods have been revised to include all communities mentioned in the results section. Please, seel line 164-168.  An explanation regarding the sample size has been added [please see line 215-220 in the revised document].  *“Sample size was estimated using a formula for a single population proportion with the assumptions of 95% confidence level (CI), 5% margin of error and an expected 35.2% prevalence of women giving birth in a health institution in the Chittagong Division, Bangladesh ^[2]^. Based on a non-response rate of 20% and the distribution of the three ethnic populations, the survey required 421 Indigenous women: 216 Chakma, 90 Marma, and 115 Tripura women”*  Justification for 36 months of delivery is stated in the Participants section; see line 173-174. |
| Lines 156 - 160: Would it not be better describing the data collection tool fully first, before describing the data collection approach? | Thank you. The data collection tool is described under Study setting; see line 164-167. |
| Lines 163 - 168: The sentence is exceedingly long and quite difficult following... please revise | Thank you, the long sentence has been split into three sentences: *Antenatal care service access data was self-reported and analysed based on the WHO’s recommendation of a minimum of four ANC-visits during pregnancy ^[3]^. Indigenous women who attended ANC services at any point during their last pregnancy were considered as having attended an ANC service. Attendance could be at a community-based health facility (community clinic, satellite clinic, school-based clinic, mother or child welfare clinic), a government hospital (district hospital or upazila health complex) or at a private facility. Attendance could also be from a skilled community health worker in their home including from a family welfa*re visitor, a community skilled birth attendant, or a health worker*. [*Please, see line 205-213] |
| Line 177: The expression "prevalence of having knowledge" - though seen it in a write-up, sounds interesting, why not simply "knowledge of ...."??? | Thank you, this sentence has been amended as suggested. |
| Lines 179 - 183: Long sentence, revise to make it easy appreciating | This sentence was amended: *Knowledge of ANC was estimated with 95% confidence intervals (CIs). The numerator was the number of women within 36 months of delivery who reported knowledge of ANC during their last pregnancy. The denominator was the total number of women within 36 months post-delivery in the two sub-districts.* [please, see line 203-208] |
| Lines 191 - 192: I do not sure of the reason behind the use chi-squares and then odd-ratios when the latter could have equally addressed the relationships of interest | The chi-squared statement has been removed. [Please, see line 279-287] |
| Lines 194 - 196: could you please provide the reasons for the varying "p-value" thresh-holds mentioned in this section. | This section has been revised as follows:  *Secondary outcomes were factors associated with knowledge of ANC services in participating women and factors associated with attendance at ANC services in the sample of women who attended at least one ANC service, Accessed using logistic regression techniques. To identify all potentially important factors those variables with a p-value ≤0.250 were purposively selected on univariate analysis to be included in a multivariable model using a backward stepwise approach ^[4]^. The final model adjusted for clustering by para (village) reported all variables with a p-value <0.100, to avoid excluding potentially important results ^[4]^. Results are reported as odds ratios (OR), adjusted odds ratios (AOR) and 95% CIs.* [see line 278-288] |
| Lines 199: "factors of interest" to what? Seems to be hanging... Or they are the "independent variables" for which the associations will be generated? | Thank you, this has been simplified and clarified, now under Variables. |
| Line 207: "Given small cell sizes, categories were collapsed to..." - Is there not the possibility that not everyone will understand the expressions 'small cell sizes" and "categories collapsed" used in this sentence? So please rephrase. | Thank you. This has been revised; see line 298-300 in the revised document. |
| Line 216 and beyond: could be "ethical considerations" instead? Provide "ethical approval number". Describe how privacy and confidentiality was maintained. | Ethics sub-heading changed to ethical considerations and ethical approval number provided.  A description of how privacy and confidentiality were maintained has been added. [please, see line: 308-318] |
| Line 222 and beyond: The results section requires some reshaping a. Would recommend you start with a brief introduction describing the response rate and some other relevant information. b. Then provide detailed descriptive information on socio-demographic characteristics and any other relevant information in a table. c. You could present information on prevalence - those could be in the same table as the socio-demographics and you could provide narratives to them. d. You then work on the associations, with their tables for knowledge and access i.e. unadjusted and adjusted OR. I do not see the importance of Chi square here as the odds ratios much more address your objectives than the former. e. Also with results of logistic regression, would it not be good to mention those that were significant in univariate analysis that were then moved into multivariate for analysis before reporting the outcome of the latter.  f. Information in figures 1 & 2 could be captured either in the tables on socio-demographics or prevalence - that should tell the story better than solitary figures... | The authors thanks the Reviewer for this comment.   a. The response rate is stated following a brief introduction. b. A summary of socio-demographic characteristics has been added. c. A brief clear statement summarises prevalence d. Chi-square statistics were used to summarise characteristics and identify associations. e. A statement has been added clarifying that variables significant on univariate analyses were included in the multivariate analyses. f. Figures 1 has been removed and reported in the text; and Figure 2 has been re-numbered as Figure 1. |
| Lines 224 - 227: Not clear on the role of this information - what is the role of CHW and Kabari??? this was not mentioned in the methods section. Also were these part of your sample??? You have also introduced new terms here Matiranga, etc. which are not mentioned anywhere else... | CHW and Karbari are identified in the methods, see line 166-167  Mataringa is also now identified in the methods, see line 159 |
| Lines 280, etc.: Ages would better be categorized as "25 - 29" and "30 and above", unless there are other reasons why that would not be possible | The categorisation have been revised accordingly. Please, see Table 1 and 3. |
| Lines 355 - 359: As you mentioned earlier, you "purposively" selected communities where data was available to address the study objectives. Could it also not be that other improvements in care delivery were in place in comparison to other places you did not select? | Thank you, this has been acknowledged, see line 478-479. |

| **Reviewer#1** | **Authors’ response** |
| --- | --- |
| General Comment: This is a well written paper which sought to assess the prevalence and factors associated with knowledge and access of Antenatal Care Services among the indigenous women in Bangladesh. I think that appropriate study methods were used in the conduct of this study. However, the authors need to beef up the method section and verify some few portions of the results sections. These are minor revisions that the authors can easily address. | Thank you for this feedback. |
| Study design and Participants Though reference has been made to a previously published paper which has full details of the methodology, I think the manuscript should be self-explanatory. Thus, it would be helpful to provide a little more information on the methods. Particularly, there should be some information about the study setting, eligibility criteria, selection of participants/sampling and sample size estimation. I think authors should briefly explain these under appropriate headings under methods as this will help to situate the study results in context. It is also not clear whether this was a facility or community-based survey? | Thank you. Study setting, eligibility criteria, selection of participants/sampling and sample size estimation are now more clearly addressed and detailed in the methods. |
| Line 202 under the data analysis, expected number of children was stated as a variable of interest but this variable was neither described nor reported on. Why was it not reported? | Thank you, this is an error and has been removed. |
| Ethics: Did participants give written/verbal consent? Also, considering the age range of participants, can you explain how you handled the ethical issues of women who were under 18 years? | This section has been revised: *Verbal informed consent was collected prior to survey interview for each participant except for those aged under 18 years. For participants aged less than 18 written consent from the participant’s guardian were collected (husband/ parents or parents-in law) and then informed verbal consent of the participant was secured.* [Please, see line 311-316] |
| Results Table 1: It appears outputs for the variables “Experienced pregnancy complications during last pregnancy” (yes//no) and “Pregnancy complications experienced according to severity” have been interchanged. This is because 99 participants responded yes to the first question and thus the various forms of severity should add up to 99, however, it is the reverse. Kindly check again. | Thanks for highlighting this error. The variable ‘Experienced pregnancy complications during last pregnancy “(yes/no)’ has been deleted from Table 1 and Table 3 as it is obsolete. |
| Table 4 Lines 211-213: How was knowledge on pregnancy-related complications categorized into mild, moderate and severe? It appears this should rather be the experience of pregnancy-related complications and not the knowledge of it. Kindly check on this. | This error has been corrected in the Tables. |
| Line 256: I am not too sure why you would cite this reference while you are presenting the study results. I think it should be taken off else it would appear as if you are already discussing the results. | Thank you. This reference has been deleted. [please, see line 372] |
| Line 328-330: Please check the odds of indigenous women who reside more than 10 kilometers from the nearest facility again. It differs from what is reported in Table 4, (the AOR from Table 4 is 0.32 (95%CI 0.09-1.1). | Thank you, this error has been corrected in the body of the manuscript; the Table result was correct. |
| Lines 414-416: Please clarify this sentence. With regards to the use of modifiable factors, I will suggest that you do away with it as captured in lines 346 and 348 to ensure consistency because right from the background no mention was made of key modifiable factors and besides, your focus is on factors, whether modifiable or not so why do you want to qualify it now. | “Modifiable” was removed from the sentence**.** |
| Figure 1 Finally, I suggest the title of Figure 1 (i.e Distribution of Indigenous women by type of ANC service received) is changed to “Distribution of Indigenous women by facility attended. I see the private facilities, community-based facilities and secondary hospital shown in the figure to be types of health facilities and not ANC services provided. The ANC services have to do with the checking of the vitals (weight, BP, etc), the urine and HIV test and detection of pregnancy-related complications among others. | Thank you. Figure 1 has been removed as it was suggested by the other reviewer. |
| Again, the figure is confusing, why is the sum of lines 3-5 in Figure 1 more (317) than the total number of women who accessed ANC at least once (232)? I think lines 3-7 in Figure 1, should add up to the total number of indigenous women who participated in the study (i.e. 438) but that is not it. | Thank you. Figure 1 has been removed as it was suggested by the other reviewer |

**In Response to Plos One comments sent via email on 03 June 2020**

| **PLos one comments** | **Response to comments** |
| --- | --- |
| 1. Please amend your current ethics statement to address the following concerns: Please explain why was written consent was not obtained, how you recorded/documented participant consent, and if the ethics committees/IRBs approved this consent procedure. | The current ethics statement has been revised – see lines 311-329 – as follows:  Although the Indigenous population in Khagrachhari district could speak and understand mainstream Bengali language, the target population consisted of many people who were illiterate or had low-literacy levels ^[5]^]. Therefore, the participant information statement was read out and explained by the study team in Bengali and all aspects of the study procedures were explained to the participants to obtain verbal informed consent. When needed, the information was explained in the participant’s local dialect. A literate impartial witness was present during consent and helped to explain the study objectives to the study participants. Participants were given ample time to consult their family members and were able to ask questions before they provided consent for participation. Verbal informed consent was collected prior to survey interview for each participant except for those aged under 18 years. For participants aged less than 18 written or verbal consent from the participant’s guardian was collected (husband/ parents or parents-in law) and then informed verbal consent of the participant was secured. Ethical approval, including permission to obtain verbal consent, was obtained from the Human Research Ethics Committee of the University of Newcastle, Australia (H-2017-0204) and the Ethics Committee of the Department of Anthropology at Jagannath University. Participation was voluntary, and privacy and confidentiality were maintained. Data were collected and reported anonymously to ensure privacy and confidentiality. |
| 2. During our internal checks, the in-house editorial staff noted that you conducted research in another country. Please check the relevant national regulations and laws applying to foreign researchers and state whether you obtained the required approvals. Please address this in your ethics statement in both the manuscript and submission information. In particular, please state whether the study was approved by a Bangladeshi ethics committee.   You indicated that you had ethical approval for your study. In your Methods section, please ensure you have also stated whether you obtained consent from parents or guardians of the minors included in the study or whether the research ethics committee or IRB specifically waived the need for their consent. | Human Research Ethical approvals were obtained in both Australia and Bangladesh as reported in the manuscript under ethical considerations – see line 323-326. The first author has an appointment as Lecturer at Jagannath University from where ethical approval was also sought. This information has also been addressed in the submission information.  A statement regarding consent from minors has been added in the Ethical considerations section – see lines 319-323.  In addition, the following statement was added to the Methods section, lines 200-206:  The first author along with two Indigenous field assistants administered the survey. The first author was a female non-Indigenous Bangladeshi researcher with an academic degree in Anthropology. The first author grew up in the region of the study site and has significant work and research experience with Indigenous women of the CHT, and with Indigenous populations globally. Interviews were conducted in the national language, Bangla. |
| 3. Please include a legend for figure 1 | A legend is provided for Figure 1. |
| 3. 1.) Please confirm whether there are legal or ethical restrictions on sharing your data.  2.) If there are ethical or legal restrictions on sharing your data, please explain them in detail (e.g., data contain potentially sensitive information, data are owned by a third-party organization, etc.) and provide the full name of the institutional body responsible for restricting the data.  3.) If there are ethical or legal restrictions on sharing the data, please confirm whether data access requests may be sent directly to [Nova@newcastle.edu.au](mailto:Nova@newcastle.edu.au)   Note that it is not acceptable for an author to be the named individual responsible for ensuring data access. 4.) If there are no restrictions, please upload the minimal anonymized data set necessary to replicate your study findings as either Supporting Information files, or to a stable public repository. Please also provide the relevant URLs, DOIs, or accession numbers for any data uploaded to a repository. For a list of acceptable repositories, please see [http://journals.plos.org/plosone/s/data-availability#loc-recommended-repositories](https://protect-au.mimecast.com/s/SQhvCL7EGotkVZJVUg3dBK?domain=journals.plos.org) | 1) There are no legal or ethical restrictions on sharing the data. Ethical approval provided by the Human Research Ethics Committee of the University of Newcastle, Australia (H-2017-0204) allows non-identifiable data to be shared with other parties to encourage scientific scrutiny and to contribute to further research and public knowledge. 2) Not applicable 3) Data requests can be sent directly to NOVA. 4) The minimal anonymized data set necessary to replicate the study findings are available from NOVA |

1.	Akter S, Davies K, Rich JL, Inder KJ. Indigenous women’s access to maternal healthcare services in lower-and middle-income countries: a systematic integrative review. International Journal of Public Health. 2018:1-11. doi: 10.1007/s00038-018-1177-4

2.	NIPORT, Mitra and Associates, ICF International. Bangladesh Demographic and Health Survey. Dhaka, Bangladesh, and Rockville, Maryland, USA: National Institute of Population Research and Training (NIPORT), Mitra and Associates and ICF International; 2014.

3.	World Health Organization. WHO recommendations on antenatal care for a positive pregnancy experience: World Health Organization; 2016.

4.	Bursac Z, Gauss CH, Williams DK, Hosmer D, editors. A purposeful selection of variables macro for logistic regression: Citeseer.

5.	Barkat A, Halim S, Poddar A, Badiuzzaman M, Osman A, Khan M, et al. Socio-economic baseline survey of Chittagong Hill Tracts. Dhaka; 2009.
