# Supplementary material for: Prevalence and factors associated with antenatal care service access among Indigenous women in the Chittagong Hill Tracts, Bangladesh: A cross-sectional study
Source: PLoS One. 2020 Dec 29;15(12):e0244640. doi: 10.1371/journal.pone.0244640 (PMC7771700; doi:10.1371/journal.pone.0244640)
Supplement: Attachment — Submitted filename: Response to Reviewer PONE-D-19-33547.docx [file pone.0244640.s004.docx]

**Akter et al.: Prevalence and factors associated with antenatal care service access among Indigenous women in the Chittagong Hill Tracts, Bangladesh: A cross-sectional study**

The authors thank the Editor and Reviewers for their valuable comments to improve the quality of this manuscript. We have addressed the recommended revisions in the manuscript using track changes and provide a detailed response to each comment below.

| **Reviewer#1** | **Authors’ response** |
| --- | --- |
| The authors have done well by revising the manuscript. Some few concerns have been noted in the revised version, and would have to be addressed before publishing this manuscript. | The authors thank the reviewer for this comment. The authors have carefully revisited the manuscript and addressed the comments to resolve the concerns. |
| **Abstract** 1. Line 61: Since the 494 women here refers to only women who met the inclusion criteria and not the total number of women in the two sub districts, I suggest you qualify it as such. Thus, the sentence could read “Of 494 indigenous women who met the inclusion criteria …… 438 participated (89% response rate) in the study”. | The sentence has been revised as suggested. Please see,  Line # 61 |
| 2. Lines 70 and 395 are not consistent: While line 70 states that “Indigenous women residing in Matiranga district (OR 6.5, 95%CI 1.7-25)”, line 395 on the other hand has it that “Indigenous women who resided at Khagrachhari Sadar sub district had 6.5 times the odds of attending ANC services”. | The authors thank the reviewer. Correction has been made in  Line#71 |
| **Introduction** 1. Line 85: I suggest you rephrase the sentence as “A significant increase in the use of maternal health care (MHC) services …….”, for a better flow. | The sentence has been revised. Please, see  Line# 85-86 |
| 2. Line 97-99: This portion of the sentence “… including about other MHC services including safe delivery ….” needs to be revised | The sentence has been revised. Please’ see  Line# 97-99 |
| 3. Line 140-142: There seem to be some omission in the sentence “…. where community- engagement was not prioritised resulted in culturally unfriendly for MHC services.” | Thank you. This sentence has been revised as follows:  ‘A recent systematic review indicated that health intervention programs for Indigenous women in LMICs where community-engagement was not prioritised as part of the development and implementation of the intervention resulted in culturally unfriendly MHC services [28]. See Line #140-143. |
| **Study Setting** 1. Line 161-165: I think this will better fit under the section on data sources in page 8, and please indicate if the data collection tool was pre-tested and the people among whom it was done. | Line#161-165 have moved under “Data sources and Measures” and suggested changes have been made. Please see Line# 222-229. |
| 2. Again under the study setting, I wish you provide some information on maternal health care services available in the area. | A new section has been added addressing maternal health care services available in Khagrachhari district. Please, see  Line# 168-182 |
| 3. Line 162: Since the individual women who participated in the study did not represent their households, I suggest the sentence is revised to read “The survey was conducted in three communities” to do away with the households. | The sentence has been revised. Please, see  Line#225-227 (as the sentence has been moved to “Data sources and Measures” section) |
| **Variables** 1. Lines 175-181: Under this section, the authors just stated the variables without indicating the outcome and independent variables. I suggest this section is reorganized to incorporate the outcome variables and lines 261-269 (under the statistical methods), under a common heading. | Independent variables and primary and secondary outcome variables are now identified, see Lines # 191-208 |
| 2. Line 261-263: “Where variable categories were less than 5 for participants’ and their partners’ education status, and for participants’ and partners’ occupation status responses were combined to allow statistical regression”. This sentence has to be revised to clarify and correct the typographical errors. • Do you mean to say that variables for education and occupation of participants and their partners had more than 5 categories and were thus categorized or the variables rather had less categories?  • How was the categorization done or which variables were combined? | Typographical errors corrected with thanks.  This sentence has been revised, see Line # 210-219 |
| 3. Line 351: Please move the “yes/no” to the variables section or an appropriate section where the various groupings of each variable is described. | ‘yes/no’ was removed from the results. |
| **Results** 1. Is line 353 “These variables, significant on univariate analysis were included in the multivariable logistic” implying that the variables stated in lines 346-354 were significant in the univariate analysis? • Why was the univariate result not presented instead of the chi-square test outputs? | The statistical methods section has been revised to clarify this; see Line # 295-299. The Chi-squared test was used to identify potentially important variables for the logistic regression analysis as shown in Table 2. Of those variables included in the logistic regression model both univariate and multivariate results are shown, See Table 3. |
| 2. Line 387: Same concern as above; why not present the univariate outputs as the multivariate analysis was dependent on results from the univariate analysis and not the chi-square test? | The statistical methods section has been revised to clarify this; see Line # 295-299. The Chi-squared test was used to identify potentially important variables for the logistic regression analysis as shown in Table 4. Of those variables included in the logistic regression model both univariate and multivariate results are shown, See Table 5. |
| 3. Please, be consistent in your report of the categories of the age (years) variable as the categories presented in the tables (16-24 years) is different from what is reported in lines 62 and 301-302 (15-29 years). | This has been corrected. All participants were 16 years and older. Women aged 15 were eligible to participate however no participants were aged 15. This has been corrected in the abstract and results. |
| 4. Lines 373-374 and Lines 439-440 seem to be contradictory. While lines 373-374 reports that “… experiencing pregnancy-related complications was not independently associated with having prior ANC knowledge”, lines 439-440 reports that “…. experiencing pregnancy-related complications …. was an important contributing factor to having knowledge about ANC…”, please reconcile these. | The authors made the correction and deleted “experiencing pregnancy-related complications”. Please, see new  Line# 478 |
